# ITDetect: a method to detect internal tandem duplication of FMS-like tyrosine kinase (FLT3) from next-generation sequencing data with high sensitivity and clinical application

**DOI:** 10.1186/s12859-023-05173-8

**Published:** 2023-02-23

**Authors:** Sungyoung Lee, Choong-Hyun Sun, Heejun Jang, Daeyoon Kim, Sung-Soo Yoon, Youngil Koh, Seung Chan Na, Sung Im Cho, Man Jin Kim, Moon-Woo Seong, Ja Min Byun, Hongseok Yun

**Affiliations:** 1 Department of Genomic Medicine, Seoul National University Hospital, Seoul, Republic of Korea; 2 Center for Precision Medicine, Seoul National University Hospital, Seoul, Republic of Korea; 3 GenomeOpinion Inc., 117-3 Hoegiro, Dongdaemoon-gu, Seoul, Republic of Korea; 4 Cancer Research Institute, Seoul National University College of Medicine, Seoul, Republic of Korea; 5 Department of Internal Medicine, Seoul National University Hospital, Seoul, Republic of Korea; 6 Department of Laboratory Medicine, Seoul National University Hospital, Seoul, Republic of Korea

**Keywords:** Internal tandem duplication, Next-generation sequencing, Acute myeloid leukemia, fragment analysis

## Abstract

Internal tandem duplication (ITD) of the FMS-like tyrosine kinase (FLT3) gene is associated with poor clinical outcomes in patients with acute myeloid leukemia. Although recent methods for detecting FLT3-ITD from next-generation sequencing (NGS) data have replaced traditional ITD detection approaches such as conventional PCR or fragment analysis, their use in the clinical field is still limited and requires further information. Here, we introduce ITDetect, an efficient FLT3-ITD detection approach that uses NGS data. Our proposed method allows for more precise detection and provides more detailed information than existing in silico methods. Further, it enables FLT3-ITD detection from exome sequencing or targeted panel sequencing data, thereby improving its clinical application. We validated the performance of ITDetect using NGS-based and experimental ITD detection methods and successfully demonstrated that ITDetect provides the highest concordance with the experimental methods. The program and data underlying this study are available in a public repository.

**Supplementary Information:**

The online version contains supplementary material available at 10.1186/s12859-023-05173-8.

## Introduction

Acute myeloid leukemia (AML) refers to a set of hematologic diseases that are characterized by acute clonal expansion of myeloid stem cells [1], mainly genetic aberrations, such as chromosomal rearrangements or oncogenic gene mutations [2]. Owing to recent advances in biological techniques, multiple genes associated with the oncogenesis, development, and prognosis of AML have been identified [3].

Among these genes, the FMS-like tyrosine kinase (FLT3) gene, a proto-oncogene that plays a crucial role in proliferation and survival, has been recognized as an important biomarker in AML. In particular, many studies have pointed out that internal tandem duplication (ITD) of the FLT3 gene (FLT3-ITD) is a poor prognostic factor [4–6]. Therefore, the detection of FLT3-ITD in clinical settings is becoming more important owing to the emergence of active FLT3 inhibitors [7].

FLT3-ITD is observed when the genomic sequences of two domains (juxtamembrane domain and tyrosine kinase domain-1) of the FLT3 gene are duplicated, with the duplication length ranging from 3 to rarely more than 200 bp [8, 9]. In addition to the duplication status and length, allelic ratio (AR) and insertion type of FLT3-ITD have been reported as important factors in predicting the prognosis of patients with AML [10–12], which also emphasizes the importance of FLT3-ITD in clinical fields [13, 14].

Conventional PCR and fragment analysis have been frequently used as experimental detection methods for the detection of FLT3-ITD, and their performance has been successfully demonstrated in extreme cases, such as those with a high mutation burden or long duplication length [12]. However, with recent advances in sequencing technology, studies have revealed the possibility of detecting FLT3-ITD using next-generation sequencing (NGS) data. Using the tremendous number of short reads that originated from the ITD region of the FLT3 gene, NGS approaches have detected FLT3-ITD either based on the structural variant (e.g., Pindel, MuTect2) or specialized (e.g., Genomon-ITDetector) algorithms [15–19], and their performance has been successfully demonstrated.

However, these methods mainly focused on the sensitive detection of ITD, and their results were often insufficient to provide clinically relevant information, such as the AR and insertion-or-duplication status. Recent studies comparing NGS-based and fragment analysis-based ARs were published [9, 16]; however, the detection tool used was not disclosed, or the insertion-or-duplication detection was not supported.

Despite the importance of AR information for clinical applications, their calculation using the existing program is only indirectly possible and remains uncertain on the correlation between the estimation and de facto standard of AR calculation, which can be provided using fragment analysis. In addition, the existence of multiple ITDs has often been discussed as one of the difficulties that existing in silico methods do not address in detail, which also reflects the AR.

To overcome the limitations of current FLT3-ITD detection methods, we introduce ITDetect, which enables accurate detection of complex FLT3-ITD and provides information that can be applied to the analysis of clinical samples. The proposed method only requires an aligned BAM file, regardless of whole-genome or exome sequencing, even that of the targeted sequencing panel, as long as the FLT3 gene is sequenced. Therefore, here, we report the comprehensive evaluation of the proposed method from various perspectives. Finally, to demonstrate the accuracy of the proposed method, we report a comparison between ITDetect and existing NGS-based and experimental methods (Additional file 1).

## Results

**Fig. 1:**
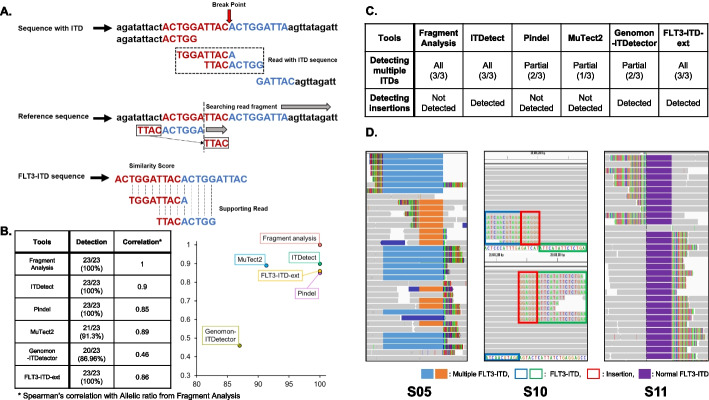
**A** The overall scheme of ITDetect Algorithm. **B** Correlation between allelic ratio from fragment analysis and allelic ratio from ITDetect. **C** Integrative genomics viewer (IGV) snapshots of our panel sequencing samples (S05, S07, S19) with multiple FLT3-ITD regions. In the validation process using panel sequencing data, we were able to identify several samples showing unusual patterns. **D** IGV screenshots of the uncommonly observed FLT3-ITDs

In the NGS data, FLT3-ITD manifests as sequence duplications; therefore, the ITD reads are mapped as soft clips to the reference sequence [20]. In this respect, we designed ITDetect to focus on soft-clipped reads (see Methods and Materials, Fig. 1A, Additional file 2: Fig. S1), which is not limited by the read length because it discovers ITDs by stitching the soft-clipped bases onto the other part of the duplicated site.

### Comparative validation of FLT3-ITD detection in targeted panel sequencing

In the panel sequencing dataset analysis, ITDetect, Pindel and FLT3-ITD-ext identified all FLT3-ITDs (23/23, 100%), whereas Genomon-ITDetector (GenomonITD) and MuTect2 detected less in two to three patients (Fig. 1B, Additional file 2: Tables S1 and S2, Fig. S2). Note that two toolsets (Breakpnt and getITD) showed substantially less detection rate (6/23 (26%) and 2/23 (9%), respectively) and were excluded from the subsequent analyses. Here, we refer to samples with FLT3-ITD detected by ITDetect as S01–S23.

Using the GRCh37 reference genome, all detected FLT3-ITDs were located to chr13:28608210-chr13:28608280, which corresponds to exon 14 in 21 samples (21/23, 96%) and chr13:28608100, which corresponds to exon 15 in one sample (1/23, 4%). Our results are supported by a previous study on FLT3-ITD ([9], exon 14 (14/243, 85%) and exon 15 (8/243, 3%)).

Using ITDetect, we also discovered three samples from the panel sequencing (S05, S07, S19; 3/23; 13%) with multiple ITD regions, and the manual curation of those multiple ITDs suggested two discoveries that strengthen the utility of ITDetect. First, the types of multiple ITDs can be classified into two categories: inclusive and overlapping. For instance, the panel sequencing sample S05 contained two ITD regions (84 and 210 bp, Fig. S7), in which one ITD region was completely included in the other ITD region. However, the multiple ITDs of samples S07 and S19 were identified in the form of partial overlap, not in which one ITD was included in the other ITD. Second, fragment analysis was not sufficient to identify multiple ITD status. In the case of S07, where two ITDs overlapped by less than 10 bp, the fragment analysis successfully confirmed multiple ITDs (two peaks of S07 in Fig. S7C). However, the multiple ITD statuses of samples with larger overlaps (84 and 64 bp for S05 and S19, respectively) were not identified using fragment analysis (one peak of S05 and S19 in Additional file 2: Fig. S7C), possibly owing to the large overlap.

In a recent study, the largest size of detected ITD was 231 bp, and ITDs above 200 bp were rarely observed [9]. Moreover, it was confirmed that the prognosis of patients with a relatively longer ITD was significantly poorer [21, 22]. In this study, an ITD over 200 bp was detected only in the S05 sample out of a total of 23 patient samples.

### Comparison of the allelic ratio (AR)

For the comparison of ARs, fragment analysis was used as the gold standard. From the estimated ARs of the 23 AML samples using fragment analysis, we divided the samples into the expected (S1 to S20, AR = 0.015–0.787, Additional file 2: Table S1) and outliers (S21–S23, AR = 1.653–16.44, Additional file 2: Table S1). To consider an effect of the outlier group, we analyzed samples without outliers (N group) and all samples (A group) separately.

We demonstrated the performance of ITDetect in two aspects: concordance and accuracy. First, the proposed method showed the highest concordance with the fragment analysis (Additional file 2: Fig. S2, Spearman’s correlation = 0.90 and 0.93 for N and A groups, respectively), compared to the other toolsets (Spearman’s correlation for N group = 0.85, 0.89, 0.46, and 0.86 and A group = 0.79, 0.88, 0.58, and 0.91 for Pindel, MuTect2, GenomonITD, and FLT3-ITD-ext, respectively) with substantial significance (*p* = 1.31 × 10^–8^, Additional file 2: Fig. S2A). Second, ITDetect showed most accurate AR estimation based on the smallest mean absolute percentage error (MAPE) that measures accuracy of estimation (MAPEs = 0.51 and 0.52 for N and A groups, respectively), compared to the other toolsets (MAPEs of N group = 0.66, 0.53, 0.83, and 1, A group = 0.7, 0.58, 0.85, and 0.92 for Pindel, MuTect2, GenomonITD, and FLT3-ITD-ext, respectively), as depicted in Additional file 2: Fig. S3).

In addition, ITDetect showed better performance when taking multiple ITDs into account. In this study, the AR of a sample with multiple ITDs was defined as the sum of the allelic ratios of individual ITDs. Except for S05 sample in all toolsets derived far different estimations than the fragment analysis, ITDetect showed MAPEs of ARs with multiple ITDs (MAPE = 0.093), compared to the other toolsets (MAPEs = 0.356, 0.513, 0.63, and 0.767 for Pindel, MuTect2, GenomonITD, and FLT3-ITD-ext), since the combined ARs were estimated more accurately by ITDetect than the compared approaches. For instance, in the case of the S19 sample with two ITDs, ITDetect estimated the AR of this sample as 0.014 (0.006 and 0.008 for each ITD, respectively; see Additional file 2: Table S1), which was closest to the estimation from the fragment analysis (0.015) and was partially or not detected in the other toolsets (Pindel AR = 0.008, FLT3-ITD-ext AR = 0.021).

### Comparative validation of FLT3-ITD detection in whole exome sequencing (WES) and conventional PCR

From the analysis of 77 WES samples, ITDetect identified eight samples with FLT3-ITD (8/77, 10%). Here, we refer to these samples as P01–P08, respectively. Similar to the analysis using panel sequencing, Pindel showed the same detection performance (8/77, 10%) as ITDetect, but showed limitations in detecting various types of ITDs that occurred in the same region. FLT3-ITD-ext showed comparable performance to ITDetect (8/77, 10%) including the multiple ITD detection, but insertion sequences were not correctly recognized in all samples with insertion. On the other hand, GenomonITD showed slightly better performance in recognizing the insertion sequence, but showed a relatively limited detection performance (5/77, 6%). Finally, MuTect2 showed the same results as GenomonITD in terms of detection performance (5/77, 6%) but provided limited information on ITD size and allele frequency.

We identified several important criteria for ITD detection using NGS data from the comparison between conventional PCR and the detection tools (Additional file 2: Table S2). First, similar to the analysis using the panel sequencing dataset, ITDetect and FLT3-ITD-ext successfully identified samples with multiple ITDs (Additional file 2: Figs. S4A, S5A, and S5H), whereas the others did not (Additional file 2: Fig. S4B), or partially detected (Pindel, Additional file 2: Fig. S5H). Second, the separation performance of ITD and additional insertion showed substantial differences, and the results showed that only ITDetect correctly separated ITD and insertion. In the samples with multiple ITDs, the two ITDs of the P08 sample actually consisted of duplication (11 and 52 bp) and insertion sequences (13 and 5 bp), as identified by ITDetect (Additional file 2: Fig. S5H). In the case of Pindel and FLT3-ITD-ext, while those toolsets correctly identified multiple ITDs, the insertion sequences were merged into the duplication estimation; therefore, the sizes of duplication became the sum of both duplication and insertion (11 + 13 and 52 + 5). In addition, MuTect2 failed to identify multiple ITDs and incorrectly estimated the ITD size (Additional file 2: Table S3).

On the other hand, for the samples with a single ITD, only ITDetect correctly separated both the ITD and insertion, whereas GenomonITD showed partial performance. For the P03 and P04 samples with large ITD and small insertions (Additional file 2: Figs. S4B, S5C, and S5D), only ITDetect and GenomonITD separated these sequences. However, GenomonITD failed to identify any ITD in P08 sample (Additional file 2: Fig. S5H).

Third, there were cases in which there were relatively few reads that presented the existence of ITD. For P06 (Additional file 2: Fig. S5F), the extremely low supporting reads limit ITD detection. Only three toolsets (ITDetect, Pindel and FLT3-ITD-ext) identified the ITD sequence.

By comparing these tools, we were able to demonstrate the sensitive detection performance of ITDetect. Pindel and FLT3-ITD-ext showed the same detection performance (8/77, 10%) as ITDetect. However, Pindel had limitations in detecting various types of ITDs that occurred in the same region. While FLT3-ITD-ext showed strong performance on detecting multiple ITDs, it had limitations in recognizing insertion sequence. On the other hand, GenomonITD showed slightly better performance in recognizing the insertion sequence, but showed a relatively limited detection performance (5/77, 6%). Finally, MuTect2 showed the same results as GenomonITD in terms of detection performance (5/77, 6%) but provided limited information on ITD size and allele frequency.

## Discussion

In this report, we introduce a novel and accurate NGS-based FLT3-ITD detection tool that provides clinical-grade information, including the insertion or duplication size and AR. To demonstrate the performance of ITDetect, two cohorts with different sequencing techniques were used in this study. During toolset development, WES data from 77 AML samples and panel sequencing data from 23 ITD-positive AML samples were used for optimization and validation. While the sequencing depth of the 77 AML WES dataset was relatively low compared to the 23 ITD-positive AML targeted sequencing datasets, the results suggest that ITDetect shows a significant correlation with the experimentally validated methods and detects FLT3-ITD with high sensitivity even when using low-depth NGS data. In this respect, ITDetect can be used for secondary analysis of pre-generated large cohorts of AML samples using the WES strategy, which will be useful for identifying the additional role of FLT3-ITD in AML.

By conducting various comparisons to the existing toolsets for detecting ITD, we suggested several considerations to detect and optimize ITDs for improving clinical applications. First, patterns of ITDetect-specific ITDs suggest the importance of accurate ITD detection, such as multiple ITDs in a single patient or insertion between repeated ITD sequences (Fig. 1 and Additional file 2: Fig. S7). Moreover, our comparison of samples with multiple ITDs shows the possibility of inaccurate estimation of allelic ratios, which is crucial to the clinical field. Second, our in-depth analysis showed the superiority of ITDetect over the compared in silico toolsets. In the case of GenomonITD, despite its utility in distinguishing ITD from insertion sequences, its low detection rate and imprecise AR estimation are problematic. While Pindel overcomes these limitations with improved detection rates and higher AR concordance than GenomonITD, incorrect ITD sequence estimates and failures in multiple ITD discoveries are not suitable for clinical purposes. MuTect2 showed intermediate performance in both discovery and AR estimation; therefore, its balanced performance might be useful for the analysis of research-purpose datasets but not for clinical-grade analysis. Finally, although FLT3-ITD-ext showed comparable performance to ITDetect and showed better AR estimation in outlier samples (S21-23), the toolset showed lower concordance with AR from the fragment analysis and lacked insertion sequence inference, which is crucial for clinical application.

Despite the usefulness of ITDetect, our study requires further investigation. First, owing to the heterogeneous treatment conditions of the target patients and insufficient follow-up periods of those samples, our preliminary findings would require subsequent analysis, which evaluates their prognostic impact in detail, with larger and homogenous cohorts. Second, compared to FLT3-ITD-ext, ITDetect showed relatively lower performance of estimating ARs of the outlier samples, which remains further study on statistical inference of ARs on those samples. Finally, the diverse ITD patterns identified by ITDetect in the panel sequencing give rise to questions regarding the role of multiple ITD in AML or predicting the response to FLT3 inhibitors and patient prognosis. Recent studies have suggested that owing to these characteristics, ITD can be a putative biomarker for predicting the prognosis of AML [13]; therefore, we believe that the application of ITDetect to large-scale WES or panel sequencing dataset of AML patients can help to answer these unsolved questions.

## Materials and methods

### Case selection

For the development of ITDectect, bone marrow samples from 77 patients with newly diagnosed, relapsed, or refractory AML were collected at the Seoul National University Hospital (SNUH) from June 2000 to December 2014. AML was diagnosed according to the WHO Classification of Hematopoietic Neoplasms, which requires the identification of 20% or more leukemic blasts in the bone marrow.

For clinical verification of ITDectect, fragment analysis of 789 AML patients was performed by the Department of Diagnostic Laboratory Medicine from May 2017 to May 2020, and the results of the targeted FiRST Hemic Panel based on targeted NGS were reviewed retrospectively. Both sets of results were available for 143 patients, and 23 patients (23/143, 16%) whose fragment analysis result was positive for FLT3-ITD were collected. This study was conducted in accordance with the Declaration of Helsinki and approved by the Institutional Review Board of SNUH (IRB No. 1611-020-805). All patients provided written informed consent at the time of sample collection.

### Whole Exome Sequencing (WES)

WES data were generated from samples collected from 77 AML patients. For exome sequencing, we captured 50 Mb of targeted exons using the SureSelect V5 and V3 Human All Exons capture kits (Agilent, Santa Clara, CA, USA). We generated 100 bp paired-end sequence reads of the captured exons using the HiSeq2000 sequencing platform (Illumina, San Diego, CA, USA) following the manufacturer’s instructions.

### Targeted gene panel sequencing

The FiRST Hemic Panel is an NGS-based customized targeted gene panel comprising 76 genes (Additional file 2: Table S3) that are recurrently mutated in myeloid neoplasms. The sequencing dataset was generated using 50 ng of DNA from bone marrow or peripheral blood samples from patients with hematologic malignancies. Library preparation was performed according to the Agilent’s SureSelect QXT Target Enrichment protocol (Agilent, Santa Clara, CA, USA). Paired-end 150-bp sequencing was performed using the NextSeq 550Dx platform (Illumina, San Diego, CA, USA).

### Bioinformatics analysis

Both the WES and FiRST Hemic Panel data were analyzed using the SNUH FiRST panel analysis pipeline. In brief, paired-end alignment to the hg19 reference genome was performed using BWA-mem (v0.7.17) [23] and GATK Best Practice [24]. After alignment, an "analysis-ready BAM" was produced and FLT3-ITD was identified (see *Cross-comparison of ITD detection tools*). The detected ITD was annotated using SnpEff (v4.3) in RefSeq.

### Conventional PCR

We reviewed the electronic medical records of SNUH for the results of conventional PCR testing for FLT3-ITD among the study participants, which was routinely performed to detect FLT3-ITD. PCR was performed using the following primer pair designed to target the FLT3 juxtamembrane domain in exon 14: forward, 3′-CCCTTCCCTTTCATCCAAGA-5′ and reverse, 3′-AACTGTGCCTCCCATTTTTG-5′. The composition of the PCR mixture is 37 μl DW, 5 μl 10X buffer, 1 μl 10 mM dNTPs, 2 μl forward primer (10 pmol/μl), 2 μl reverse primer (10 pmol/μl), 0.25 μl Taq polymerase (5 U/μl), and 3 μl DNA (100 ng/μl). The total volume of the PCR mixture was 50 μl. The PCR conditions were 30 cycles of DNA denaturation for 30 s at 94 °C, primer annealing for 30 s at 60 °C, and primer extension for 1 min at 72 °C. The PCR product was 574 bp.

### ITDetect algorithm

Some of the reads that aligned as soft-clipped to the reference sequence [23] were aligned to the ITD positions because of sequence duplication (Fig. 1A). ITDetect uses the following algorithm to identify FLT3-ITD (Additional file 2: Fig. S1):

***Step 1.*** Scan the positions where reads are aligned as soft-clipped in the FLT3 gene region (e.g., chr13:28577000-28676800 in GRCh37). These positions were considered possible ITD breakpoints. To avoid false alignments, reads with a soft-clipped alignment size less than 10bp were not used for ITD detection.

***Step 2.*** For each position, determine whether the soft-clipped reads align with a position that supports an ITD breakpoint. For example, if the 3′ end of a read is properly aligned and the 5′ end is soft-clipped, ITDetect searches for the soft-clipped sequence in the reference genome within 1000 bp from the end of the read alignment. As the size of the ITD decreases, the position where the soft-clipped sequence aligns becomes closer to the end position of the read alignment. ITD may not be duplicated exactly because of PCR artifacts or biologically pathogenic variants. A similarity search using dynamic programming considers mismatched bases in the reference sequence and supports robust duplication detection. Similarly, if the 3′ end of a sequence is soft-clipped, ITDetect searches for the soft-clipped sequence in the reference genome within 1000 bp before the start of the read alignment. If a soft-clipped sequence is detected in the reference sequence, the sequence is considered to be a potential ITD. If the 5’ end of the read is soft-clipped, then the portion of the reference sequence from the alignment start position to the end position of the soft-clipped sequence is considered a duplicated sequence (i.e., an ITD). ITDs often involve insertions between duplications. A search for the entire soft-clipped sequence in the reference genome does not detect such variants. To address this issue, ITDetect iteratively searches for soft-clipped sequences while decreasing the size of the sequence to 10 bp (Additional file 2: Fig. S1).

***Step 3.*** For each candidate ITD, ITDetect counted the number of aligned reads. First, it generates a predicted ITD sequence based on the results of step two. Next, it counts the number of reads that align with the breakpoint in the predicted sequence. A minimum of three aligned reads were required to designate an ITD.

***Step 4.*** For the filtered ITD candidates with high-frequency AR (estimated AR > 1), ITDetect applies an additional AR estimation step. To correctly estimate AR, all reads within the ITD region were aligned to the ITD sequence using Smith-Waterman algorithm and included AR estimated under an optimized thresholds (alignment score difference < 15 and non-overlapping duplication sequence at both end < 16).

***Step 5***. Additional filtering algorithms were applied after ITD calling. When multiple ITD detection results appear at the same position, the highest alt read count is determined as true, and it is selected as the final result.

In addition, ITDetect requires a BAM file generated by an aligner that supports soft clipping because it uses soft-clipped reads for initial detection.

### Cross-comparison of ITD detection tools

To evaluate the accuracy and sensitivity of ITDetect, we compared its performance with those of six other tools developed to detect SVs: Pindel [18], Genomon-ITDetector [19], MuTect2 [17], FLT3-ITD-ext [16], getITD [25], and Breakpnt [9]. All the toolsets were acquired by either downloading from the official repository or requesting from the authors. Genomic regions around FLT3 were extracted from the final BAM files. The resultant BAM file was assigned as an input, including other inputs that were adjusted as equally as possible (Additional file 2: Table S4). When using Pindel, short insertions were defined as duplicates if they were located at the FLT3 locus. All identified ITDs were manually curated by clinical experts in laboratory medicine using integrative Genomics Viewer (IGV) [26].

### Fragment analysis

Quantification of the FLT3-ITD allelic ratio was performed using fragment analysis. Fluorescently labeled PCR primers used were as follows: F-5′-GCAATTTAGGTATGAAAGCCAGC-3′ and R-5′-CTTTCAGCATTTTGACGGCAACC-3′. Amplicon electrophoresis was run on an ABI PRISM 3130 Genetic Analyzer (Applied Biosystems, Foster City, CA, USA) and analyzed using GeneMapper v.3.7 (Applied Biosystems, Foster City, CA, USA). The FLT3-ITD allelic ratio was calculated by dividing the peak height of the ITD product by that of the wild-type allele.

## Supplementary Information

**Additional file 1.** Supplmentary texts and Legends for Supplementary Tables and Figures.

**Additional file 2.** Supplementary Tables and Figures.

## Data Availability

The dataset we used in the analysis is available on the public repository (https://github.com/cpmsnuh/itdetect).
