# Supplementary material for: ITDetect: a method to detect internal tandem duplication of FMS-like tyrosine kinase (FLT3) from next-generation sequencing data with high sensitivity and clinical application
Source: BMC Bioinformatics. 2023 Feb 23;24:62. doi: 10.1186/s12859-023-05173-8 (PMC9951415; doi:10.1186/s12859-023-05173-8)
Supplement: Supplementary file 1 — Additional file 1. Supplmentary texts and Legends for Supplementary Tables and Figures. [file 12859_2023_5173_MOESM1_ESM.docx]

**ITDetect: A method to detect internal tandem duplication of FMS-like tyrosine kinase (FLT3) from next-generation sequencing data with high sensitivity and clinical application**

**Supplementary Materials**

**Text S1. ITDetect Material and Methods**

***Case Seletion***

For the development of ITDectect, bone marrow samples from 77 patients with newly diagnosed, relapsed, or refractory AML diagnosis were collected at Seoul National University Hospital (SNUH) from June 2000 to December 2014. The diagnosis of AML was made according to the WHO Classification of Hematopoietic Neoplasms, which requires identification of 20% or more leukemic blasts in the bone marrow.

For the clinical verification of ITDectect, fragment analysis of 789 AML patients performed by the Department of Diagnostic Laboratory Medicine from May 2017 to May 2020, and the results of the targeted FiRST Hemic Panel based on targeted NGS was reviewed retrospectively. There were 143 patients in whom both sets of results were available, and 23 patients (23/143, 16%) whose fragment analysis result was positive for FLT3-ITD were collected. This study was conducted according to the Declaration of Helsinki and was approved by the institutional review board of SNUH (IRB No. 1201-099-396). All patients gave informed consent at the time of sample collection.

***Whole Exome Sequencing (WES)***

WES data were generated from samples collected from 77 patients with AML. For exome sequencing, we captured 50Mb targeted exons using the SureSelect V5 and V3 Human All Exons capture kits (Agilent, Santa Clara, CA, USA). We generated 100bp paired-end sequence reads of the captured exons using the HiSeq2000 sequencing platform (Illumina, San Diego, CA, USA) following the manufacturer’s instructions.

***Targeted gene panel sequencing***

FiRST Hemic Panel is an NGS-based customized, targeted gene panel consisting of 76 genes (**Table S3**) that are recurrently mutated in myeloid neoplasms. Dataet was generated using 50 ng of DNA from bone marrow or peripheral blood samples from patients with hematologic malignancy. Library preparation was performed according to Agilent’s SureSelectQXT Target Enrichment protocol (Agilent, Santa Clara, CA, USA). Paired-end 150-bp sequencing was using NextSeq 550Dx platform (Illumina, San Diego, CA, USA).

***Bioinformatics analysis***

Both the WES and FiRST Hemic Panel data were analyzed using the SNUH FiRST Panel Analysis Pipeline. In brief, paired-end alignment to hg19 reference genome was performed using BWA-mem (v0.7.17) (Li *et al*., 2009) and the GATK Best Practice (McKenna *et al*., 2010). After the alignment, an "analysis-ready BAM" was produced and variants such as SNV, InDel, CNV, and translocation were identified using the following tools: GATK UnifiedGenotyper (v4.1.6) (McKenna *et al*., 2010), SNVer (v0.5.3) (Wei *et al*., 2011) and LoFreq (v2.1.2) (Wilm *et al*., 2012) for SNV/InDel detection; Delly (v0.8.1) (Rausch *et al*., 2012) and Manta (v1.5.0) (Chen *et al*., 2016) for translocation discovery; and CNVKit (v0.9.5) (Talevich *et al*., 2016) for purity estimation and CNV calling. Detected variants were annotated by SnpEff (v4.3) with various databases such as RefSeq, COSMIC, dbSNP, ClinVar, and gnomAD.

***Conventional PCR***

We reviewed the electronic medical records of SNUH for the results of conventional PCR testing for FLT3-ITD among the study participants, which were routinely performed to detect FLT3-ITD. The PCR is performed with the following primer pair designed to target the FLT3 juxtamembrane domain in exon 14: forward, 3’-CCCTTCCCTTTCATCCAAGA-5’; reverse, 3’-AACTGTGCCTCCCATTTTTG-5’. The composition of the PCR mixture is 37μl DW, 5μl 10X buffer, 1μl 10mM dNTPs, 2μl forward primer (10pmol/μl), 2μl reverse primer (10pmol/μl), 0.25μl Taq polymerase (5U/μl), and 3μl DNA (100ng/μl). The total volume of the PCR mixture was 50μl. The PCR cycle conditions were 30 cycles of DNA denaturation for the 30s at 94°C, primer annealing for 30s at 60°C, and primer extension for 1 min at 72°C. The length of the PCR product was 574bp.

***ITDetect algorithm***

Some of the reads that align as soft-clipped to the reference sequence (Li *et al*., 2009) are aligned to ITD positions because of sequence duplication (**Fig. 1A**). ITDetect uses the following algorithm to identify FLT3-ITD (see also **Fig. S1**):

*Step 1*. Scan the positions where reads are aligned as soft-clipped in the FLT3 gene region (e.g., chr13:28577000-28676800 in GRCh37). Those positions were considered possible ITD breakpoints. To avoid false alignments, reads with a soft-clipped alignment size less than 10bp were not used for ITD detection.

*Step 2.* For each position, determine whether the soft-clipped reads align to a position that supports an ITD breakpoint. For example, if the 3’ end of a read is properly aligned and the 5’ end is soft-clipped, ITDetect searches for the soft-clipped sequence in the reference genome within 1,000bp from the end of the read alignment. As the size of the ITD decreases, the position where the soft-clipped sequence aligns gets closer to the end position of the read alignment. ITD may not be duplicated exactly due to PCR artifacts or biological pathogenic variants. A similarity search using a dynamic programming considers mismatched bases to reference sequence and supports robust duplication detection. Similarly, if the 3’ end of a sequence is soft-clipped, ITDetect searches for the soft-clipped sequence in the reference genome within the 1,000bp before the start of the read alignment.

If the soft-clipped sequence is detected in the reference sequence, that sequence is considered to be a potential ITD. If the 5’ end of the read is soft-clipped, then the portion of the reference sequence from the alignment start position to the end position of the soft-clipped sequence is considered to be a duplicated sequence (i.e., an ITD).

ITDs often include an insertion between duplications. A search for the entire soft-clipped sequence in the reference genome will not detect such variants. To address that issue, ITDetect iteratively searches for the soft-clipped sequences while decreasing the size of the sequence down to 10bp (**Fig. S1**).

*Step 3*. For each candidate ITD, ITDetect counts the number of aligned reads. It first generates a predicted ITD sequence based on the results of step two. Then, it counts the number of reads that align with the breakpoint in the predicted sequence. A minimum of three aligned reads is required to designate an ITD.

*Step 4*. For the filtered ITD candidates with high-frequency AR (AR > 1), ITDetect applies an additional AR estimation step. To correctly estimate AR, all reads within the ITD region were aligned to the ITD sequence using Smith-Waterman algorithm and included AR estimated under an optimized thresholds (alignment score difference < 15 and non-overlapping duplication sequence at both end < 16).

*Step 5*. Additional filtering algorithms are applied after ITD calling. When multiple ITD detecting results appearing in the same position, the highest alt read count is determined as true, and it is selected as the final result.

In addition, ITDetect requires a BAM file generated by an aligner that supports soft-clipping, since it uses soft-clipped reads for initial detection.

***Cross-comparison of ITD detection tools***

To evaluate the accuracy and sensitivity of ITDetect, we compared its performance to the existing tools for detecting SVs. We considered the following tools: Pindel (Ye *et al*., 2009), Genomon-ITDetector (Chiba *et al*., 2015), MuTect2 (Benjamin *et al*., 2019), Breakpnt (He *et al.*, 2020), FLT3-ITD-ext (Tsai *et al.*, 2020), and getITD (Blätte *et al.*, 2019). All the toolsets were downloaded from their public repository except Breakpnt which was acquired by contacting the author. For the comparison, the BAM files were first subsetted to FLT3-region, and the subsetted file was assigned as an input, including the other inputs which were adjusted as equally as possible (**Table S4**). When using Pindel, short insertions were defined as duplicates if they were located in the FLT3 gene locus. Integrative Genomics Viewer (IGV) (Robinson *et al*., 2017) was used to validate detection results by manually confirming soft-clipped reads align at the left and right breakpoints of the FLT3-ITD region.

***Fragment Analysis***

Quantification of the FLT3-ITD allelic ratio was performed by fragment analysis. Fluorescently labeled PCR primers were used as follows: F-5’-GCAATTTAGGTATGAAAGCCAGC-3’ and R-5'-CTTTCAGCATTTTGACGGCAACC-3'. Electrophoresis of amplicons was run on the ABI PRISM 3130 Genetic Analyzer (Applied Biosystems, Foster City, CA, USA) and analyzed using GeneMapper v.3.7 (Applied Biosystems, Foster City, CA, USA). FLT3-ITD allelic ratio was calculated by dividing the peak height of the ITD product by that of the normal wild type allele.

**Text S2. Details of ITDetect results**

***Comparative validation of FLT3-ITD detection in targeted panel sequencing***

Using GRCh37 reference genome, all the detected FLT3-ITDs were located to chr13:28608210-chr13:28608280 that corresponds to exon 14 in 21 samples (21/23, 96%) and to chr:28608100 that corresponds to exon 15 in 1 sample (1/23, 4%), respectively. Our results were supported by a previous study on the FLT3-ITD (Rong *et al.*, exon 14 (14/243, 85%) and exon 15 (8/243, 3%)).

Multie ITDs were found on the panel sequencing (S05, S07, S19; 3/23; 13%). However, it was confirmed that the longer ITD was not only in the form containing the shorter ITD (S05; **Fig. S6E**), but only a part of the two different ITDs was matched. Fragment analysis showed that there were two peaks in the resulting picture in the S07 and S19 samples, which confirmed that there are two ITDs (S07, S19; **Figs. S6G** and **S6S**).

The duplication sizes of FLT3-ITD were 8bp to 210bp. In a recent study, the maximum value of ITD detected was 231 bp, and it was reported that ITDs above 200 bp were observed very rarely (He *et al*., 2020). Also, it was confirmed that the prognosis of patients with relatively longer ITD was significantly poor (Fathi et al., 2011; Liu et al., 2019). In this study, ITD over 200bp was detected only in S05 sample out of a total of 23 patient samples.

There was a result of analyzing the possibility of clinical use by using the size of ITD, and we referenced this to determine whether it has clinical significance by dividing it into two groups based on the median value (62bp) of all detected ITDs. Of the 23 samples, 10 samples (10/23, 43%) had ITDs longer than 62bp and the remaining 10 samples had ITDs with a length of 62bp or less.

***Comparison of the allelic ratio***

When calculating the allelic ratio in ITDetect, if multiple ITDs were detected in one sample, the sum of the allelic ratios of each of the detected ITDs was determined as the total allelic ratio (AR) of the sample. In the case of the S19 sample, the AR value in the fragment analysis was 0.015, and the ARs ​​of the two ITDs detected in ITDetect were 0.006 and 0.008, respectively, and when these were added together, the result was 0.014, which was close to the value in the fragment analysis.

Generally, fragment analysis showed a higher allelic ratio in most samples (17/20, 85%). It was confirmed that the result of ITDetect was higher in 3 of 20 samples, and those were 45bp, 168bp, 210bp (S11, S03, S05), respectively. In the case of S11, there was a difference of 0.01. In the case of S03 and S05, the difference in ITDetect results was 0.05 or more, showing a higher allelic ratio. This is a case in which the size of the duplication of ITD is relatively larger than that of other ITDs, and in the case of fragment analysis, it is considered to be due to the limitation of the detection efficiency of the long ITD.

***Comparative validation of FLT3-ITD detection in WES and conventional PCR***

In the process of detecting FLT3-ITD using ITDetect, several results that can be an important criterion for ITD detection using NGS data were confirmed, from the comparison between the conventional PCR and the detection tools (**Table S4**). First, two types of ITD with different lengths starting from the same position were detected. In the case of the P01 sample, two types of ITDs with duplication sizes of 39bp and 75bp, respectively, were detected in the ITDetect results, and these were also confirmed on IGV (**Fig. S2**).

In the case of another patient, the P08 sample, it was confirmed that two types of ITDs were detected, 11bp and 52bp starting at different locations. In the results of Pindel and MuTect2, it was confirmed that different lengths were presented in the detected results, which is the difference depending on whether each tool recognizes the insertion separately. In the case of the P08 specimen, ITDetect recognized the insertion of 'CATATTCATATTC' and 'TCGAA' for the two detected ITDs and classified them separately. In the case of Pindel and MuTect2, the insertion was considered to be combined with the ITD and the length of the structural variant was determined. When the ITD size detected by ITDetect and the length of the insertion were summed, it was confirmed that the length was the same as that of Pindel, MuTect2 and FLT3-ITD-ext (**Figs. S4** and **S6; Table S2**). And, when ITDs of multiple lengths were detected in one sample, it was confirmed that the longer ITD contained the shorter ITD. Only ITDetect detected ITDs of all lengths in both samples.

Second, there were cases where there were separate insertions before or after the ITD. In the detection result of the P03 sample, a short insertion was confirmed, and the insertion and ITD were also confirmed in IGV (**Figs. S4B** and **S5C**). In addition to the P03 sample, the detection results were also confirmed by ITDetect for the samples P04 and P08 with confirmed insertions (**Figs. S5D** and **S5H**). Most of the tools could detect the ITD, but there was a difference in the printed results. In the case of ITDetect and Genomon-ITDetector, in the output result of ITD including separate insertion, the sequence of insertion together with the ITD sequence is separately marked and output. On the other hand, in the case of Pindel and Mutect2, it was confirmed that the corresponding size was added to the duplication size of ITD and printed in the result without indicating separate insertion.

Third, there were cases where there were relatively few alternative reads. In the case of the P06 sample, the number of alt reads was 4 in the detection result of ITDetect, and a relatively small number of alt read counts were confirmed even on IGV (**Figs. S4B** and **S5F**). Only ITDetect, Pindel, and FLT3-ITD-ext detected ITD in the sample.

Through the comparison between tools, we were able to prove the sensitive detection performance of ITDetect. In the case of Pindel, it showed the same detection performance (8/77, 10%) as ITDetect, but it showed limitations in detecting various types of ITDs that occurred in the same region. On the other hand, Genomon-ITDetector showed a little more strength in recognizing the insertion sequence but showed a relatively limited detection performance (5/77, 6%). Finally, MuTect2 showed the same results as Genomon-ITDetector in terms of detection performance (5/77, 6%), but provided limited information on ITD size and allele frequency.

**Supplementary Figure Legends**

**Fig. S1. Schematic overview of ITDetect workflow.** In the development step, we validated our tool comparing with conventional PCR and other NGS-based FLT3-ITD detecting tools using WES data from 77 AML patients. And we used another 23 ITD-positive AML samples for clinical validation. We discovered the correlation of the allelic ratio between fragment analysis and ITDetect.

**Fig. S2. Comparison of allelic ratios (ARs) between the fragment analysis and *in silico* ITD detection tool** (A) ITDetect, (B) Pindel, (C) Mutect2, (D) Genomon-ITDetector, and (E) FLT3-ITD-ext.

**Fig. S3. Accuracies of FLT3-ITD detection algorithms.** Vertical axis represents mean absolute percentage errors (MAPEs) which measures estimation errors of the AR estimation, therefore the lower MAPE indicates better accuracy. MAPEs of two groups (N group with 20 samples without 3 outlier samples, and A group with all of 23 samples) were drawn separately, and the MAPEs of five algorithms were depicted.

**Fig. S4. FLT3-ITD features from WES data and conventional PCR. (**A) FLT3-ITD region from IGV capture and Conventional PCR result (P01, P03, P06). (B) Detailed information of FLT3-ITD from NGS based detecting Tools. Three cases considered to be important for ITD detection, as shown in figure 3. The first case is when various sizes of ITD exist. As in the case of P01, the detection results from IGV and ITDetect were able to identify two ITDs with different sizes. NGS-based tools except ITDetect detected only one type of ITD. The second case is when the insertion is inserted between ITDs. The third case is when the ITD has a small Alt read count. In the case of P06 in Figure 3, For ITD in the P06 sample observed in IGV Capture, ITDetect and Pindel detected the ITD.

**Fig. S5. Detailed information and the captured images of the FLT3-ITD region from the WES data.** (P01 – P08)

**Fig. S6. Detailed information and the captured images of the FLT3-ITD region from the panel sequencing data.** (S01 – S23)

**Fig S7. Multiple FLT3-ITD samples from panel sequencing data and fragment analysis.** (A) Multiple FLT3-ITD regions from IGV capture (S05, S07, S19). (B) Detailed information of FLT3-ITD from NGS based detecting Tools. (C) Detected multiple FLT3-ITD from fragment analysis. We compared the results of fragment analysis and other NGS-based tools for samples in which multiple FLT3-ITDs were detected in our tool.

**Supplementary Table Legends**

**Table S1.** The overall result of internal tandem duplication (ITD)-positive acute myeloid leukemia (AML) patients from fragment analysis and NGS-based tools by using panel sequencing data.

**Table S2.** The overall result of internal tandem duplication (ITD)-positive acute myeloid leukemia (AML) patients from conventional PCR and NGS-based tools by using whole exome sequencing (WES) data.

**Table S3.** Target gene list of FiRST-Hemic Panel (76 genes).

**Table S4.** Parameters of internal tandem duplication (ITD) detection tools used in the analyses.

**Reference**

Li, H. and R. Durbin (2009). "Fast and accurate short read alignment with Burrows-Wheeler transform." Bioinformatics **25**(14): 1754-1760.

McKenna, A., et al. (2010). "The Genome Analysis Toolkit: a MapReduce framework for analyzing next-generation DNA sequencing data." Genome Res **20**(9): 1297-1303.

Wei, Z., et al. (2011). "SNVer: a statistical tool for variant calling in analysis of pooled or individual next-generation sequencing data." Nucleic Acids Res **39**(19): e132.

Wilm, A., et al. (2012). "LoFreq: a sequence-quality aware, ultra-sensitive variant caller for uncovering cell-population heterogeneity from high-throughput sequencing datasets." Nucleic Acids Res **40**(22): 11189-11201.

Rausch, T., et al. (2012). "DELLY: structural variant discovery by integrated paired-end and split-read analysis." Bioinformatics **28**(18): i333-i339.

Chen, X., et al. (2016). "Manta: rapid detection of structural variants and indels for germline and cancer sequencing applications." Bioinformatics **32**(8): 1220-1222.

Talevich, E., et al. (2016). "CNVkit: Genome-Wide Copy Number Detection and Visualization from Targeted DNA Sequencing." PLoS Comput Biol **12**(4): e1004873.

Benjamin, D., et al. (2019) “Calling somatic SNVs and indels with mutect2”. BioRxiv 861054., <https://doi.org/10.1101/861054>.

Ye, K., et al. (2009). "Pindel: a pattern growth approach to detect break points of large deletions and medium sized insertions from paired-end short reads." Bioinformatics **25**(21): 2865-2871.

Chiba, K., et al. (2015). "Genomon ITDetector: a tool for somatic internal tandem duplication detection from cancer genome sequencing data." Bioinformatics **31**(1): 116-118.

Li, H., et al. (2009). "The Sequence Alignment/Map format and SAMtools." Bioinformatics **25**(16): 2078-2079.

He, R., et al. (2020). "Hybridization capture-based next generation sequencing reliably detects FLT3 mutations and classifies FLT3-internal tandem duplication allelic ratio in acute myeloid leukemia: a comparative study to standard fragment analysis." Modern Pathology **33**(3): 334-343.

Fathi, A. T. and Y. B. Chen (2011). "Treatment of FLT3-ITD acute myeloid leukemia." Am J Blood Res **1**(2): 175-189.

Robinson, J. T., et al. (2017). "Variant Review with the Integrative Genomics Viewer." Cancer Research **77**(21): e31-e34.

Liu, S. B., et al. (2019). "Impact of FLT3-ITD length on prognosis of acute myeloid leukemia." Haematologica **104**(1): e9-e12.
