# Supplementary material for: ITDetect: a method to detect internal tandem duplication of FMS-like tyrosine kinase (FLT3) from next-generation sequencing data with high sensitivity and clinical application
Source: BMC Bioinformatics. 2023 Feb 23;24:62. doi: 10.1186/s12859-023-05173-8 (PMC9951415; doi:10.1186/s12859-023-05173-8)
Supplement: Supplementary file 2 — Additional file 2. Supplementary Tables and Figures. [file 12859_2023_5173_MOESM2_ESM.pptx]

## Slide 1
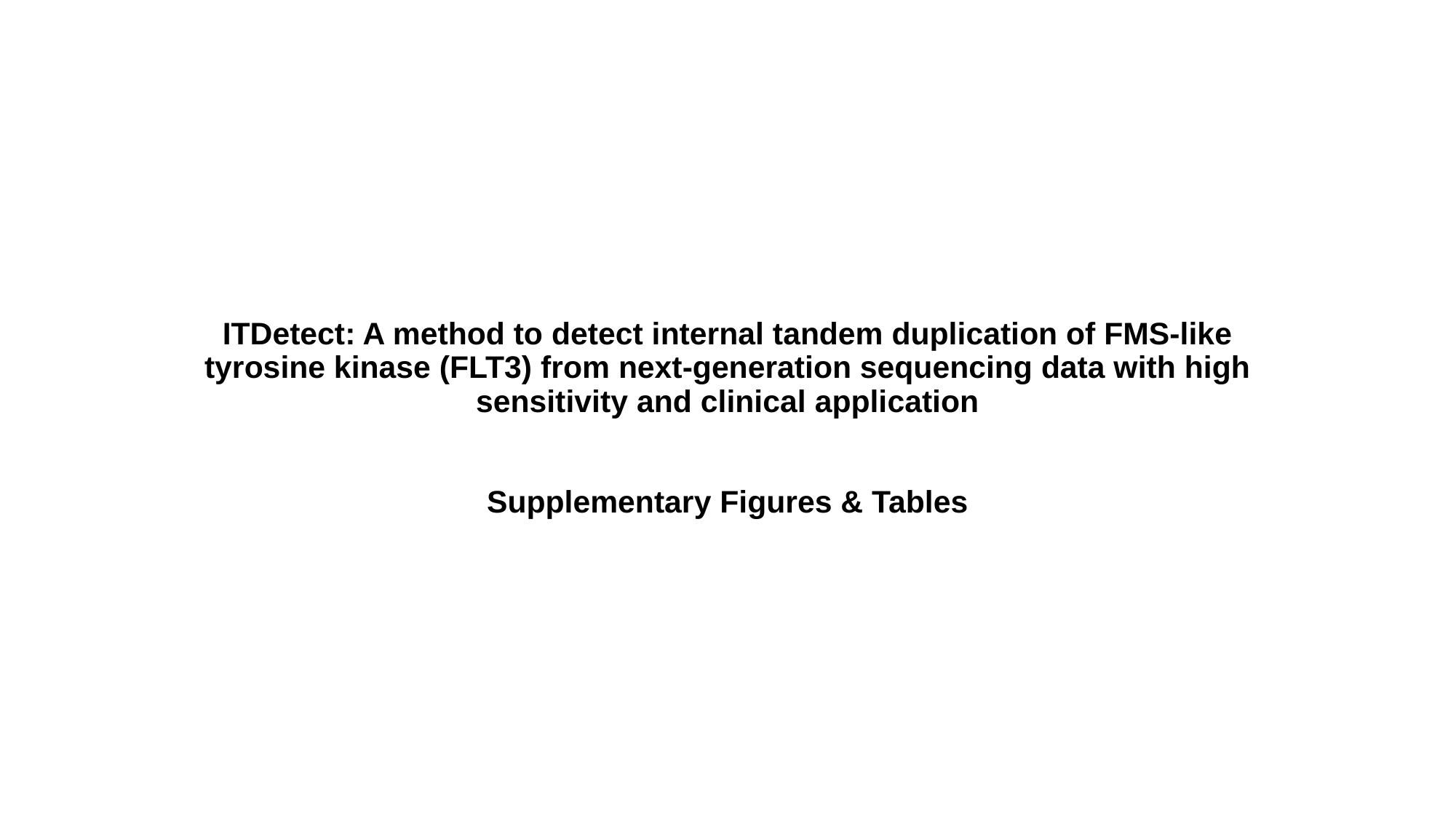

# ITDetect: A method to detect internal tandem duplication of FMS-like tyrosine kinase (FLT3) from next-generation sequencing data with high sensitivity and clinical application Supplementary Figures & Tables

## Slide 2
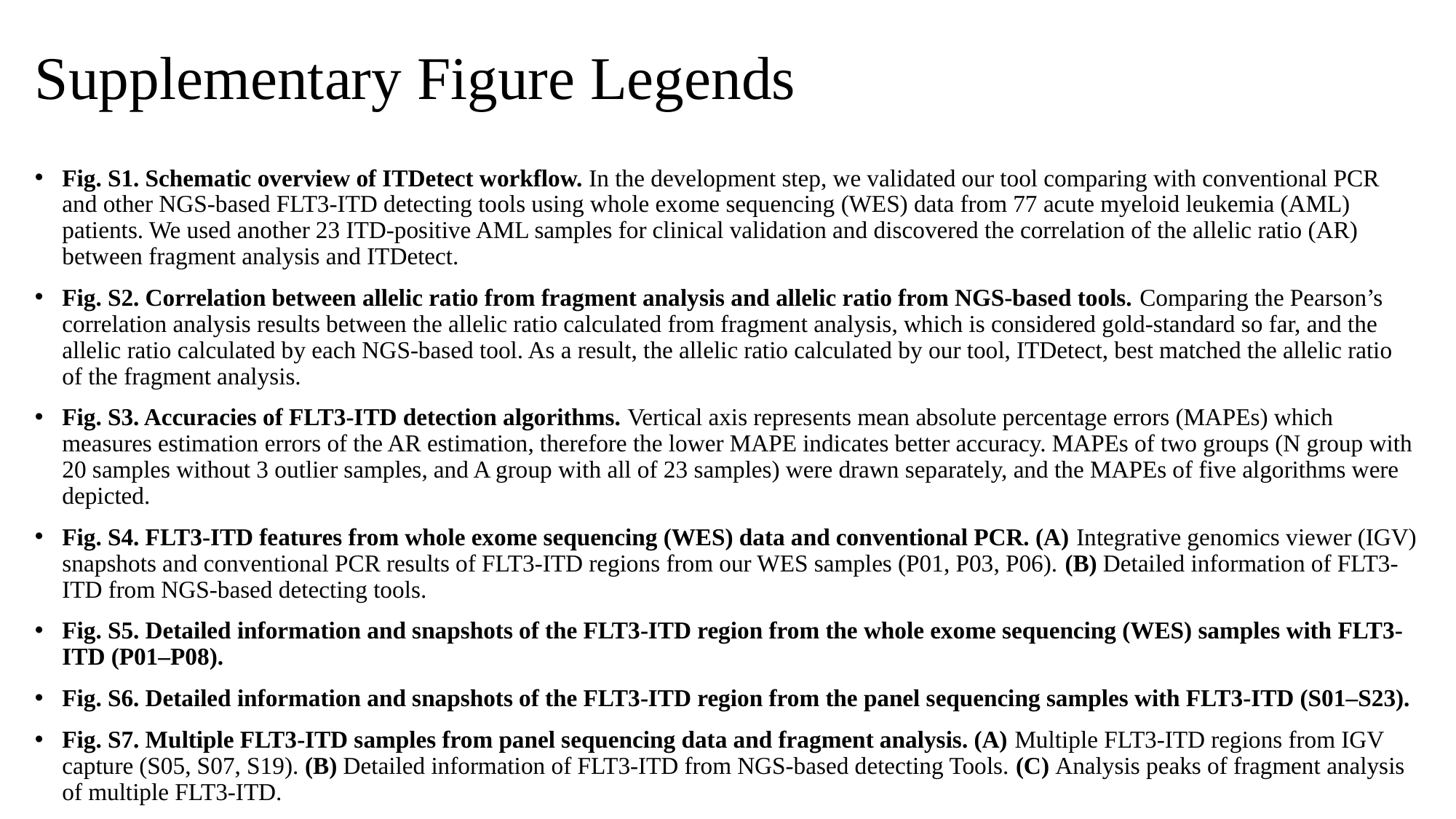

# Supplementary Figure Legends
Fig. S1. Schematic overview of ITDetect workflow. In the development step, we validated our tool comparing with conventional PCR and other NGS-based FLT3-ITD detecting tools using whole exome sequencing (WES) data from 77 acute myeloid leukemia (AML) patients. We used another 23 ITD-positive AML samples for clinical validation and discovered the correlation of the allelic ratio (AR) between fragment analysis and ITDetect.
Fig. S2. Correlation between allelic ratio from fragment analysis and allelic ratio from NGS-based tools. Comparing the Pearson’s correlation analysis results between the allelic ratio calculated from fragment analysis, which is considered gold-standard so far, and the allelic ratio calculated by each NGS-based tool. As a result, the allelic ratio calculated by our tool, ITDetect, best matched the allelic ratio of the fragment analysis.
Fig. S3. Accuracies of FLT3-ITD detection algorithms. Vertical axis represents mean absolute percentage errors (MAPEs) which measures estimation errors of the AR estimation, therefore the lower MAPE indicates better accuracy. MAPEs of two groups (N group with 20 samples without 3 outlier samples, and A group with all of 23 samples) were drawn separately, and the MAPEs of five algorithms were depicted.
Fig. S4. FLT3-ITD features from whole exome sequencing (WES) data and conventional PCR. (A) Integrative genomics viewer (IGV) snapshots and conventional PCR results of FLT3-ITD regions from our WES samples (P01, P03, P06). (B) Detailed information of FLT3-ITD from NGS-based detecting tools.
Fig. S5. Detailed information and snapshots of the FLT3-ITD region from the whole exome sequencing (WES) samples with FLT3-ITD (P01–P08).
Fig. S6. Detailed information and snapshots of the FLT3-ITD region from the panel sequencing samples with FLT3-ITD (S01–S23).
Fig. S7. Multiple FLT3-ITD samples from panel sequencing data and fragment analysis. (A) Multiple FLT3-ITD regions from IGV capture (S05, S07, S19). (B) Detailed information of FLT3-ITD from NGS-based detecting Tools. (C) Analysis peaks of fragment analysis of multiple FLT3-ITD.

## Slide 3
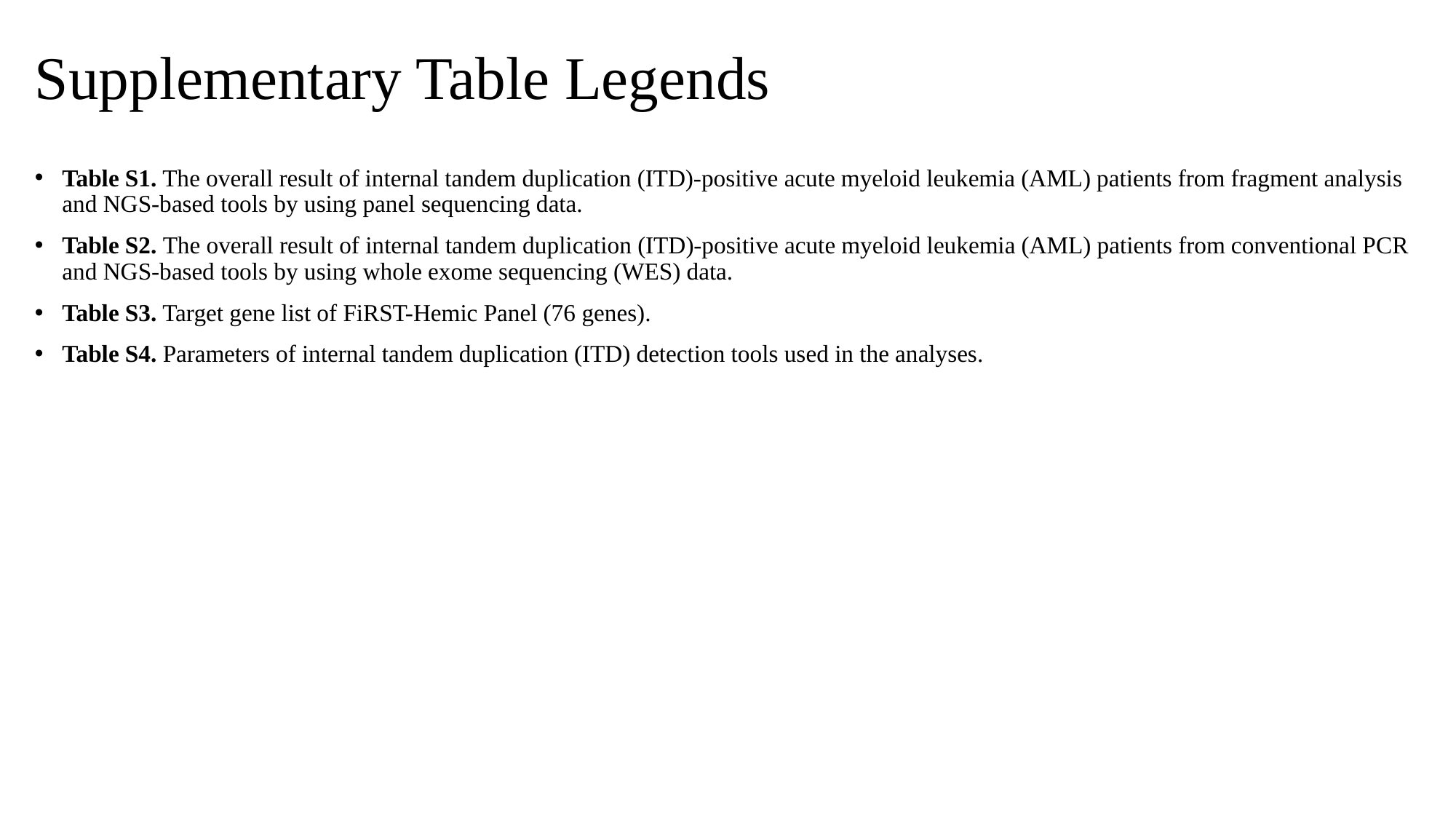

# Supplementary Table Legends
Table S1. The overall result of internal tandem duplication (ITD)-positive acute myeloid leukemia (AML) patients from fragment analysis and NGS-based tools by using panel sequencing data.
Table S2. The overall result of internal tandem duplication (ITD)-positive acute myeloid leukemia (AML) patients from conventional PCR and NGS-based tools by using whole exome sequencing (WES) data.
Table S3. Target gene list of FiRST-Hemic Panel (76 genes).
Table S4. Parameters of internal tandem duplication (ITD) detection tools used in the analyses.

## Slide 4
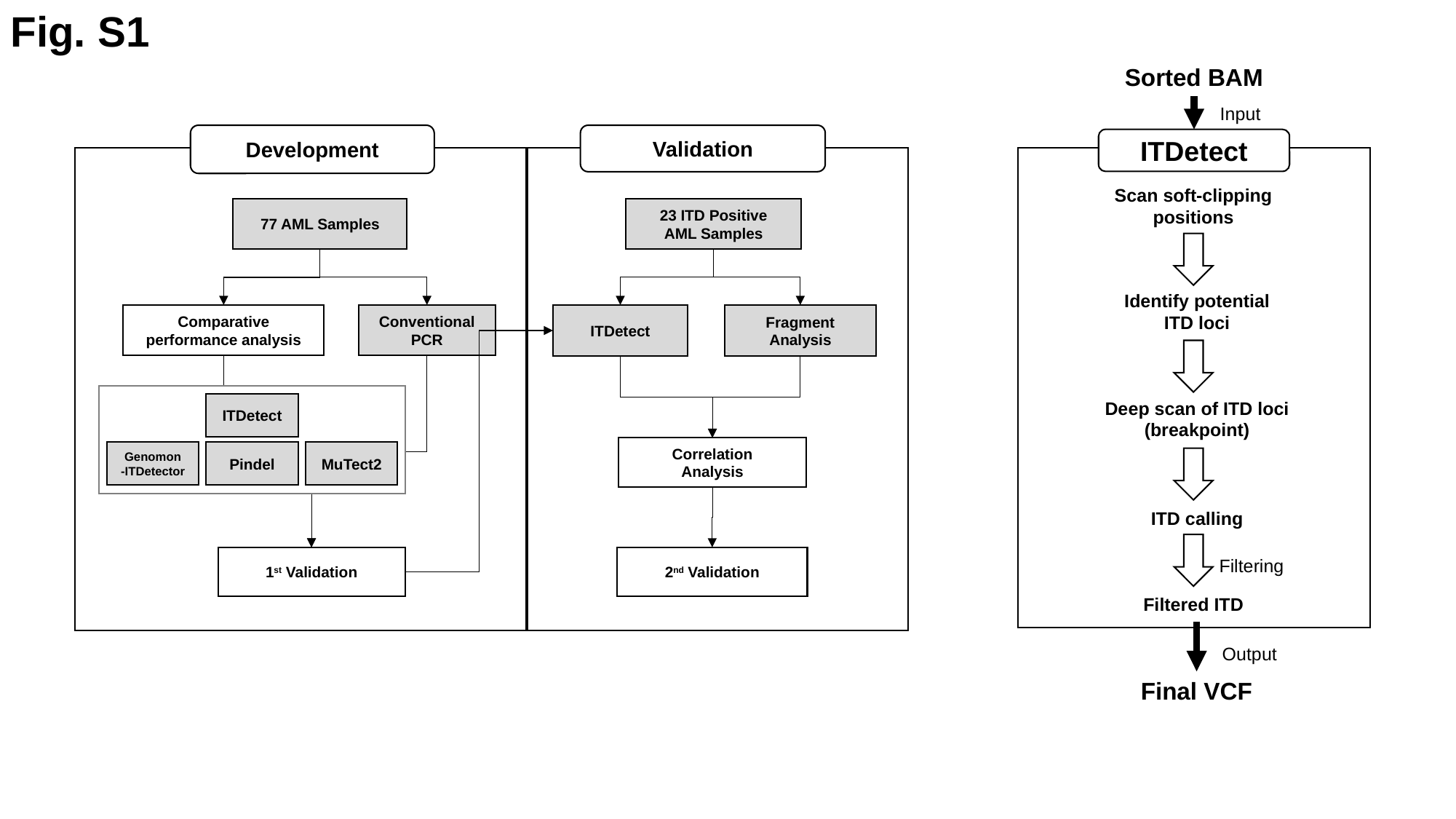

Fig. S1
Sorted BAM
Input
Development
Validation
ITDetect
Scan soft-clipping
positions
23 ITD Positive
AML Samples
77 AML Samples
Identify potential
ITD loci
Conventional
PCR
Comparative performance analysis
ITDetect
Fragment Analysis
Deep scan of ITD loci
(breakpoint)
ITDetect
Correlation
Analysis
Genomon
-ITDetector
Pindel
MuTect2
ITD calling
1st Validation
2nd Validation
Filtering
Filtered ITD
Output
Final VCF

## Slide 5
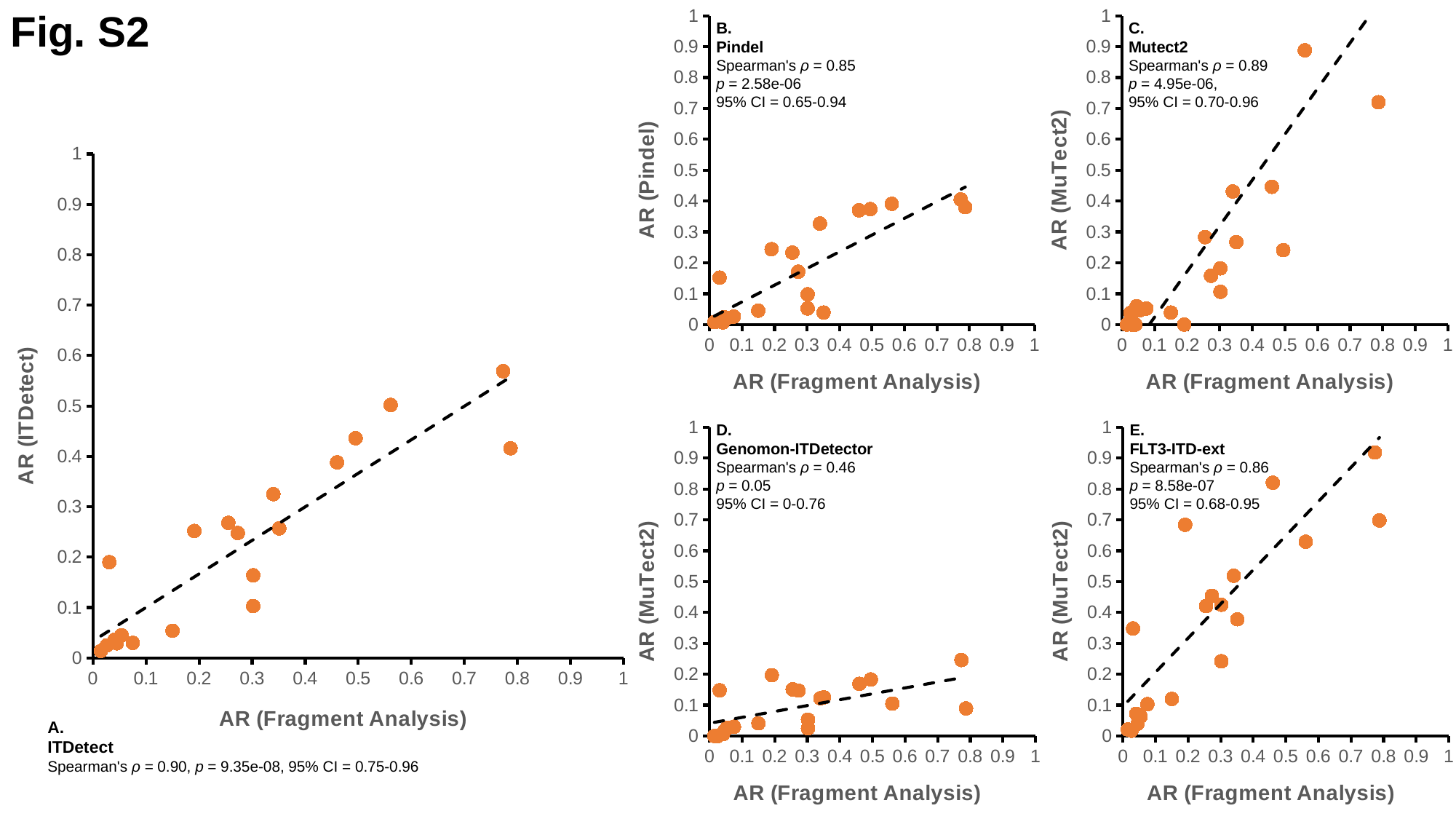

### Chart
| Category | |
|---|---|
### Chart
| Category | |
|---|---|
### Chart
| Category | |
|---|---|
### Chart
| Category | |
|---|---|
### Chart
| Category | | |
|---|---|---|Fig. S2
B.
Pindel
Spearman's ρ = 0.85
p = 2.58e-06
95% CI = 0.65-0.94
C.
Mutect2
Spearman's ρ = 0.89
p = 4.95e-06,
95% CI = 0.70-0.96
D.
Genomon-ITDetector
Spearman's ρ = 0.46
p = 0.05
95% CI = 0-0.76
E.
FLT3-ITD-ext
Spearman's ρ = 0.86
p = 8.58e-07
95% CI = 0.68-0.95
A.
ITDetect
Spearman's ρ = 0.90, p = 9.35e-08, 95% CI = 0.75-0.96

## Slide 6
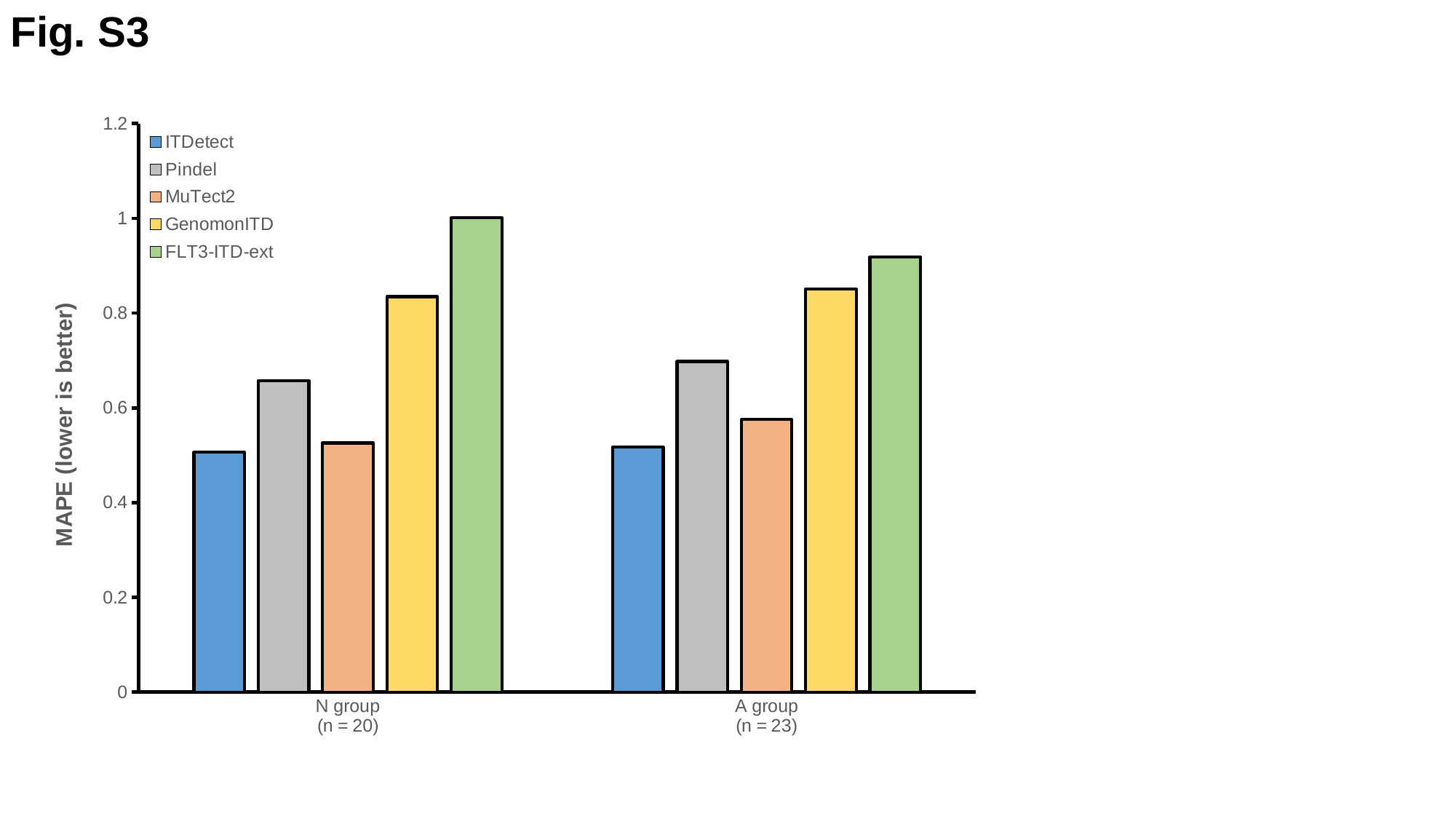

Fig. S3
### Chart
| Category | ITDetect | Pindel | MuTect2 | GenomonITD | FLT3-ITD-ext |
|---|---|---|---|---|---|
| N group
(n = 20) | 0.5062 | 0.6575 | 0.5259 | 0.8347 | 1.001 |
| A group
(n = 23) | 0.5168 | 0.6979 | 0.5755 | 0.8509 | 0.9188 |

## Slide 7
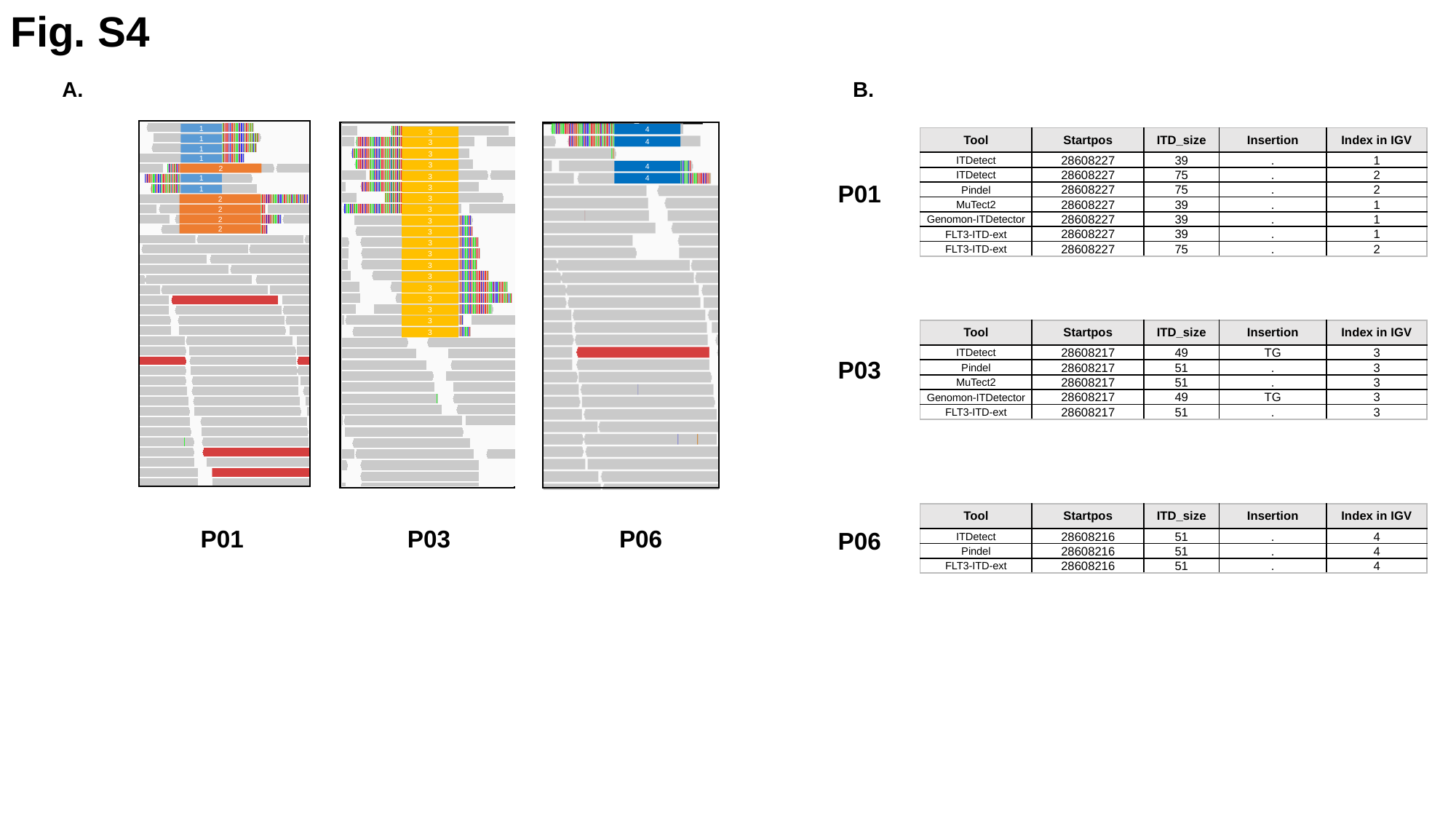

Fig. S4
A.
B.
1
4
3
1
4
3
1
3
1
3
4
2
3
4
1
3
1
3
2
3
2
2
3
2
3
3
3
3
3
3
3
3
3
3
| Tool | Startpos | ITD\_size | Insertion | Index in IGV |
| --- | --- | --- | --- | --- |
| ITDetect | 28608227 | 39 | . | 1 |
| ITDetect | 28608227 | 75 | . | 2 |
| Pindel | 28608227 | 75 | . | 2 |
| MuTect2 | 28608227 | 39 | . | 1 |
| Genomon-ITDetector | 28608227 | 39 | . | 1 |
| FLT3-ITD-ext | 28608227 | 39 | . | 1 |
| FLT3-ITD-ext | 28608227 | 75 | . | 2 |
P01
| Tool | Startpos | ITD\_size | Insertion | Index in IGV |
| --- | --- | --- | --- | --- |
| ITDetect | 28608217 | 49 | TG | 3 |
| Pindel | 28608217 | 51 | . | 3 |
| MuTect2 | 28608217 | 51 | . | 3 |
| Genomon-ITDetector | 28608217 | 49 | TG | 3 |
| FLT3-ITD-ext | 28608217 | 51 | . | 3 |
P03
| Tool | Startpos | ITD\_size | Insertion | Index in IGV |
| --- | --- | --- | --- | --- |
| ITDetect | 28608216 | 51 | . | 4 |
| Pindel | 28608216 | 51 | . | 4 |
| FLT3-ITD-ext | 28608216 | 51 | . | 4 |
P01
P03
P06
P06

## Slide 8
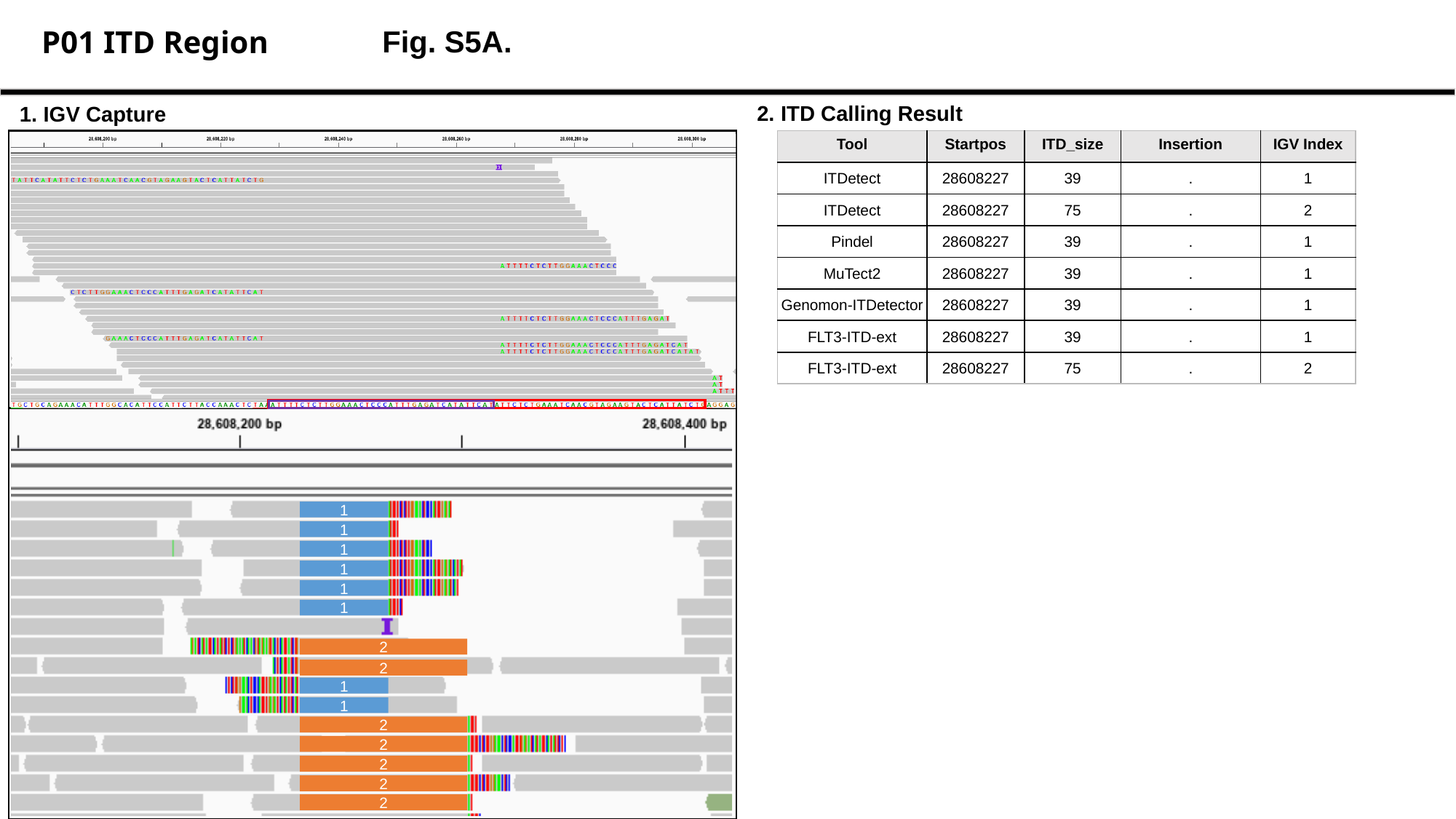

Fig. S5A.
P01 ITD Region
2. ITD Calling Result
1. IGV Capture
| Tool | Startpos | ITD\_size | Insertion | IGV Index |
| --- | --- | --- | --- | --- |
| ITDetect | 28608227 | 39 | . | 1 |
| ITDetect | 28608227 | 75 | . | 2 |
| Pindel | 28608227 | 39 | . | 1 |
| MuTect2 | 28608227 | 39 | . | 1 |
| Genomon-ITDetector | 28608227 | 39 | . | 1 |
| FLT3-ITD-ext | 28608227 | 39 | . | 1 |
| FLT3-ITD-ext | 28608227 | 75 | . | 2 |
1
1
1
1
1
1
1
1
2
2
2
2
2
2
2

## Slide 9
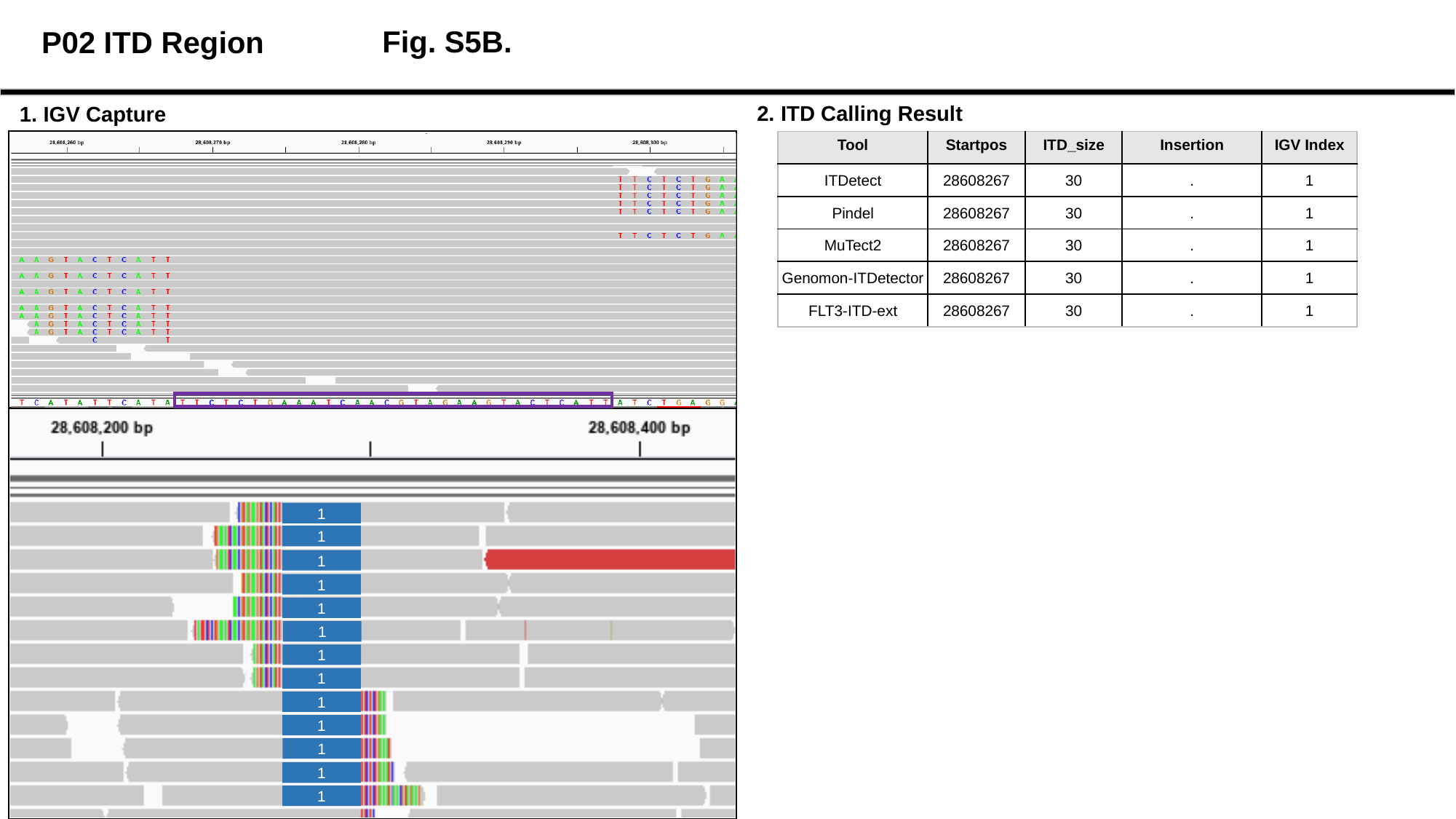

Fig. S5B.
P02 ITD Region
2. ITD Calling Result
1. IGV Capture
| Tool | Startpos | ITD\_size | Insertion | IGV Index |
| --- | --- | --- | --- | --- |
| ITDetect | 28608267 | 30 | . | 1 |
| Pindel | 28608267 | 30 | . | 1 |
| MuTect2 | 28608267 | 30 | . | 1 |
| Genomon-ITDetector | 28608267 | 30 | . | 1 |
| FLT3-ITD-ext | 28608267 | 30 | . | 1 |
1
1
1
1
1
1
1
1
1
1
1
1
1

## Slide 10
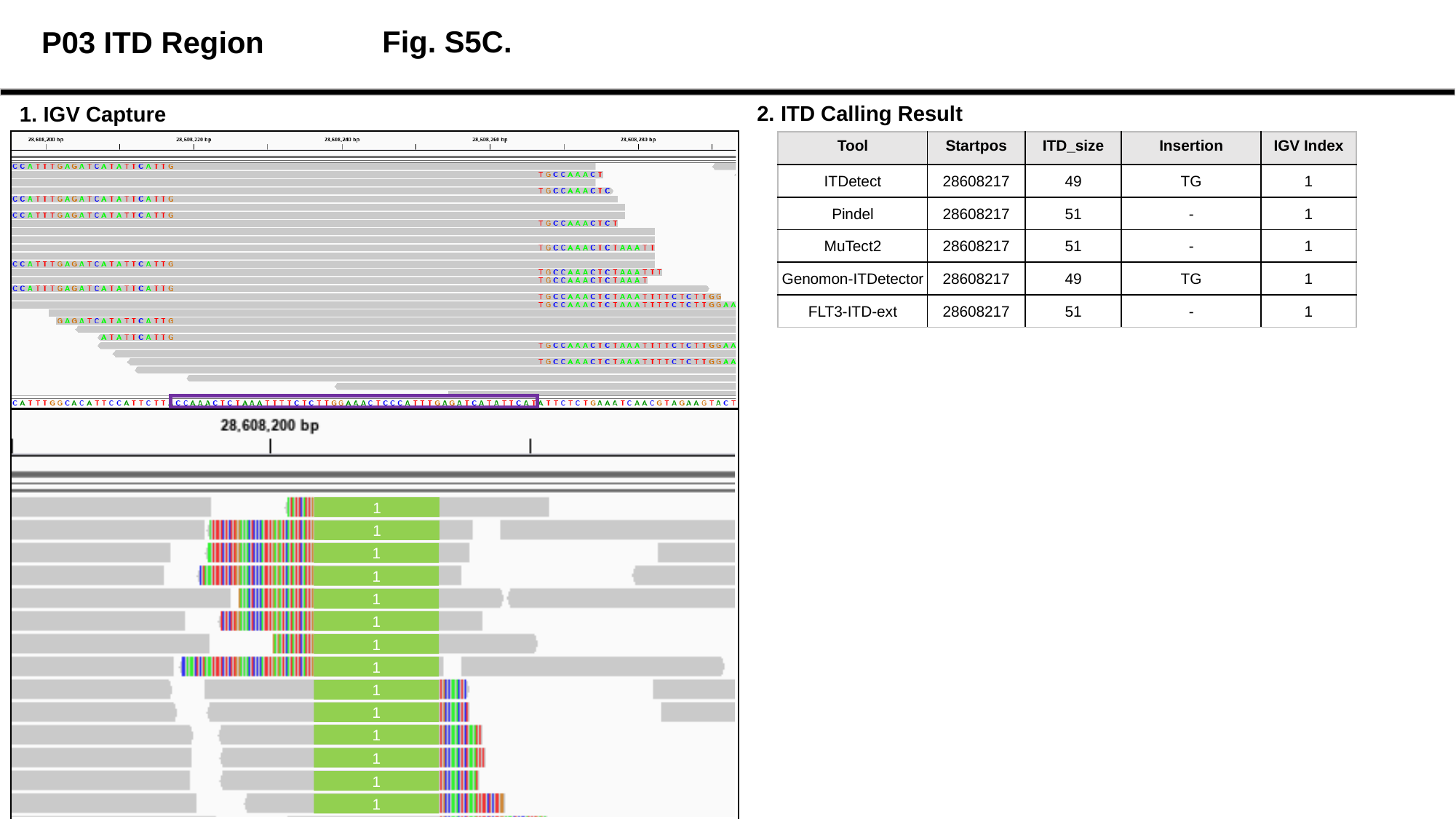

Fig. S5C.
P03 ITD Region
2. ITD Calling Result
1. IGV Capture
| Tool | Startpos | ITD\_size | Insertion | IGV Index |
| --- | --- | --- | --- | --- |
| ITDetect | 28608217 | 49 | TG | 1 |
| Pindel | 28608217 | 51 | - | 1 |
| MuTect2 | 28608217 | 51 | - | 1 |
| Genomon-ITDetector | 28608217 | 49 | TG | 1 |
| FLT3-ITD-ext | 28608217 | 51 | - | 1 |
1
1
1
1
1
1
1
1
1
1
1
1
1
1

## Slide 11
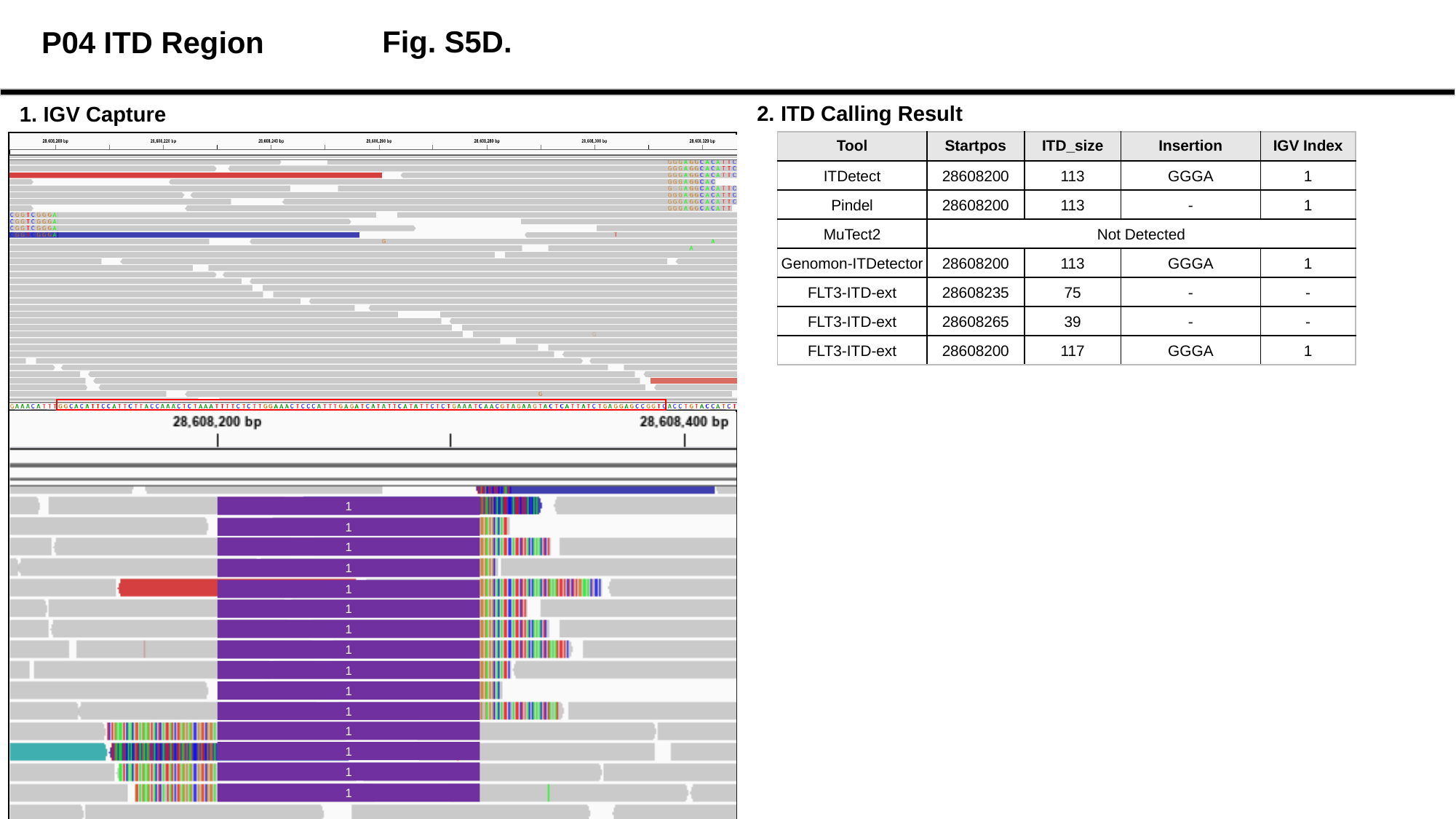

Fig. S5D.
P04 ITD Region
2. ITD Calling Result
1. IGV Capture
| Tool | Startpos | ITD\_size | Insertion | IGV Index |
| --- | --- | --- | --- | --- |
| ITDetect | 28608200 | 113 | GGGA | 1 |
| Pindel | 28608200 | 113 | - | 1 |
| MuTect2 | Not Detected | | | |
| Genomon-ITDetector | 28608200 | 113 | GGGA | 1 |
| FLT3-ITD-ext | 28608235 | 75 | - | - |
| FLT3-ITD-ext | 28608265 | 39 | - | - |
| FLT3-ITD-ext | 28608200 | 117 | GGGA | 1 |
1
1
1
1
1
1
1
1
1
1
1
1
1
1
1

## Slide 12
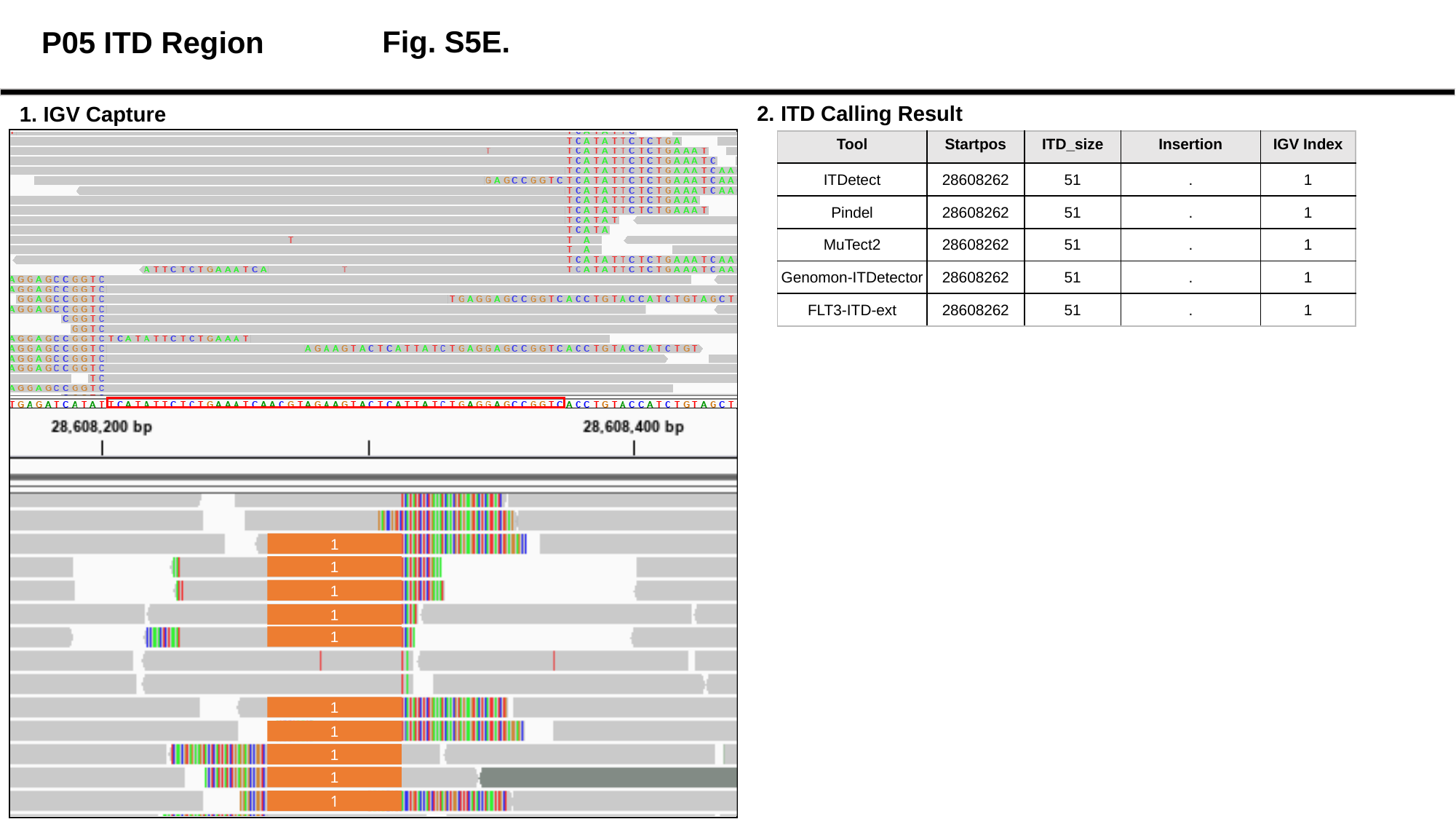

Fig. S5E.
P05 ITD Region
2. ITD Calling Result
1. IGV Capture
| Tool | Startpos | ITD\_size | Insertion | IGV Index |
| --- | --- | --- | --- | --- |
| ITDetect | 28608262 | 51 | . | 1 |
| Pindel | 28608262 | 51 | . | 1 |
| MuTect2 | 28608262 | 51 | . | 1 |
| Genomon-ITDetector | 28608262 | 51 | . | 1 |
| FLT3-ITD-ext | 28608262 | 51 | . | 1 |
1
1
1
1
1
1
1
1
1
1

## Slide 13
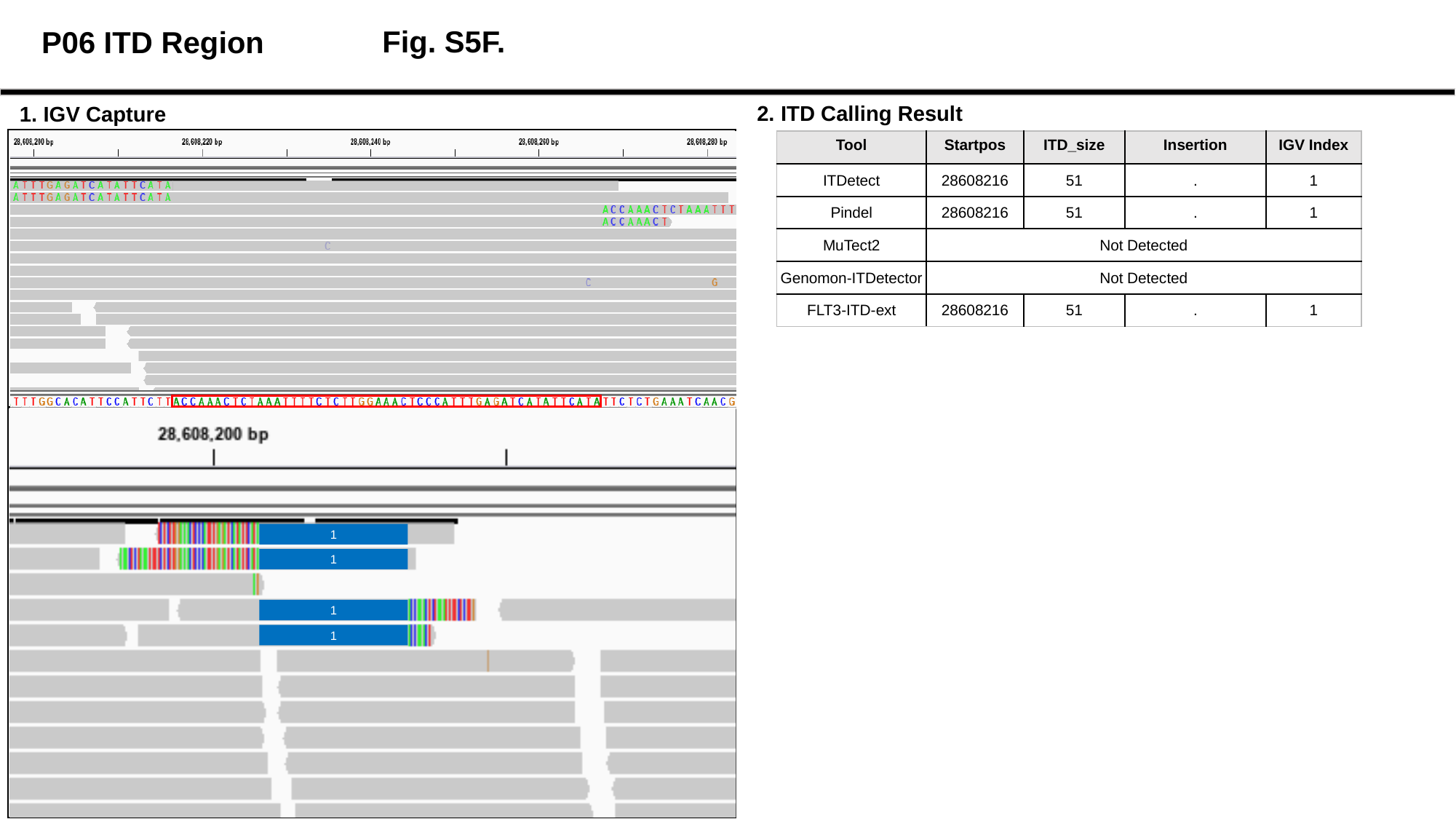

Fig. S5F.
P06 ITD Region
2. ITD Calling Result
1. IGV Capture
| Tool | Startpos | ITD\_size | Insertion | IGV Index |
| --- | --- | --- | --- | --- |
| ITDetect | 28608216 | 51 | . | 1 |
| Pindel | 28608216 | 51 | . | 1 |
| MuTect2 | Not Detected | | | |
| Genomon-ITDetector | Not Detected | | | |
| FLT3-ITD-ext | 28608216 | 51 | . | 1 |
1
1
1
1

## Slide 14
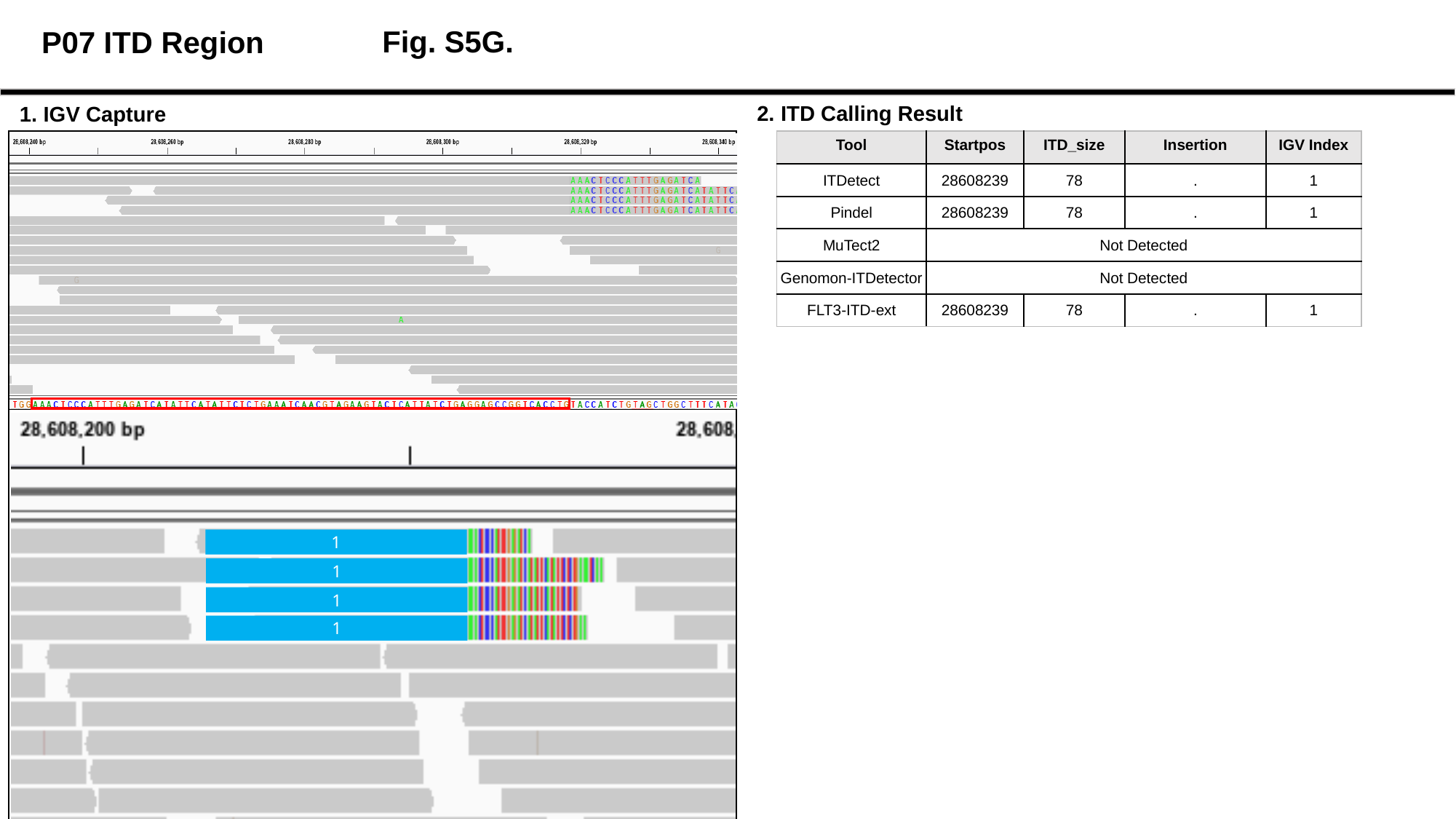

Fig. S5G.
P07 ITD Region
2. ITD Calling Result
1. IGV Capture
1
1
1
1
| Tool | Startpos | ITD\_size | Insertion | IGV Index |
| --- | --- | --- | --- | --- |
| ITDetect | 28608239 | 78 | . | 1 |
| Pindel | 28608239 | 78 | . | 1 |
| MuTect2 | Not Detected | | | |
| Genomon-ITDetector | Not Detected | | | |
| FLT3-ITD-ext | 28608239 | 78 | . | 1 |

## Slide 15
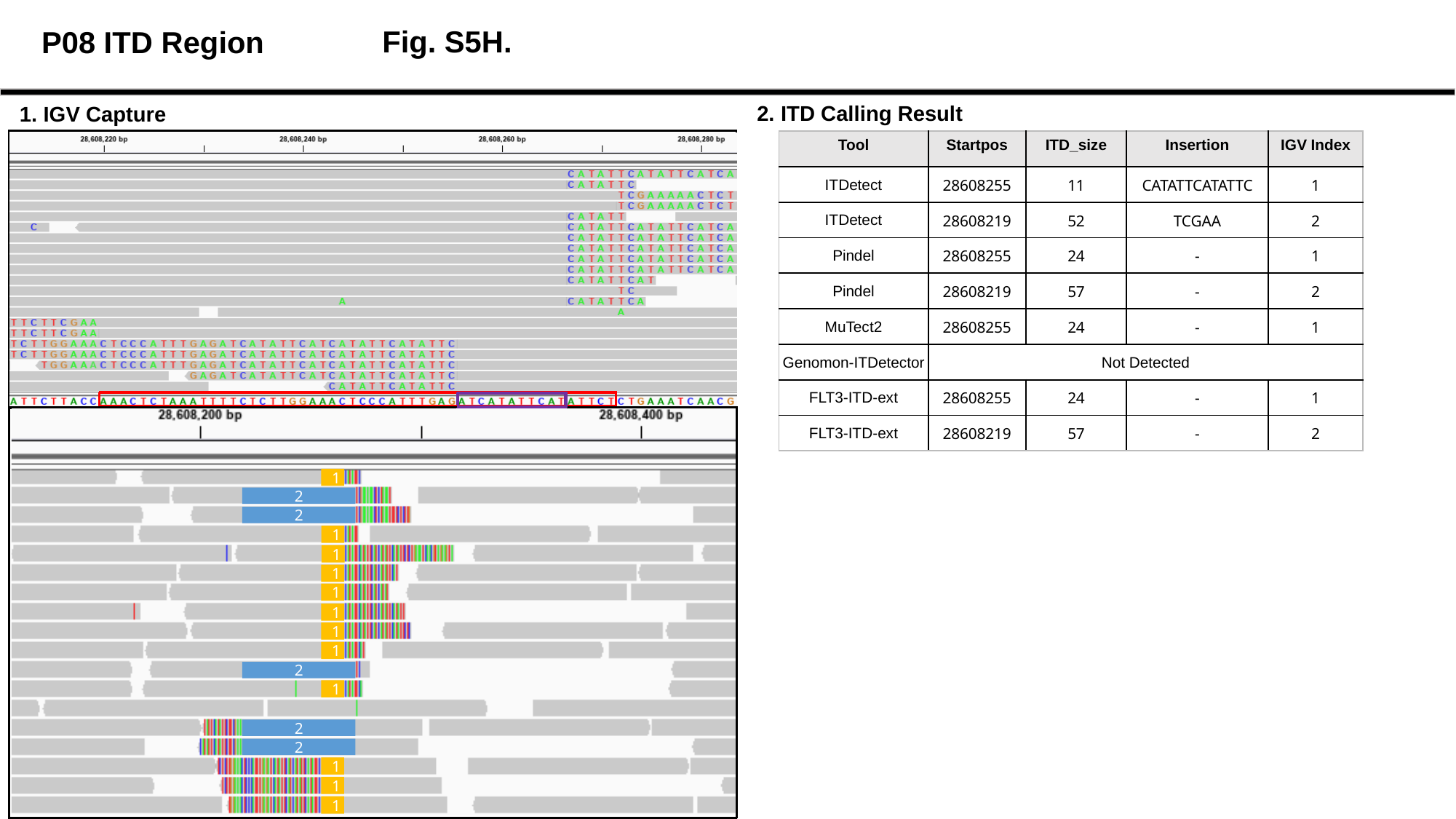

Fig. S5H.
P08 ITD Region
2. ITD Calling Result
1. IGV Capture
| Tool | Startpos | ITD\_size | Insertion | IGV Index |
| --- | --- | --- | --- | --- |
| ITDetect | 28608255 | 11 | CATATTCATATTC | 1 |
| ITDetect | 28608219 | 52 | TCGAA | 2 |
| Pindel | 28608255 | 24 | - | 1 |
| Pindel | 28608219 | 57 | - | 2 |
| MuTect2 | 28608255 | 24 | - | 1 |
| Genomon-ITDetector | Not Detected | | | |
| FLT3-ITD-ext | 28608255 | 24 | - | 1 |
| FLT3-ITD-ext | 28608219 | 57 | - | 2 |
1
2
2
1
1
1
1
1
1
1
2
1
2
2
1
1
1

## Slide 16
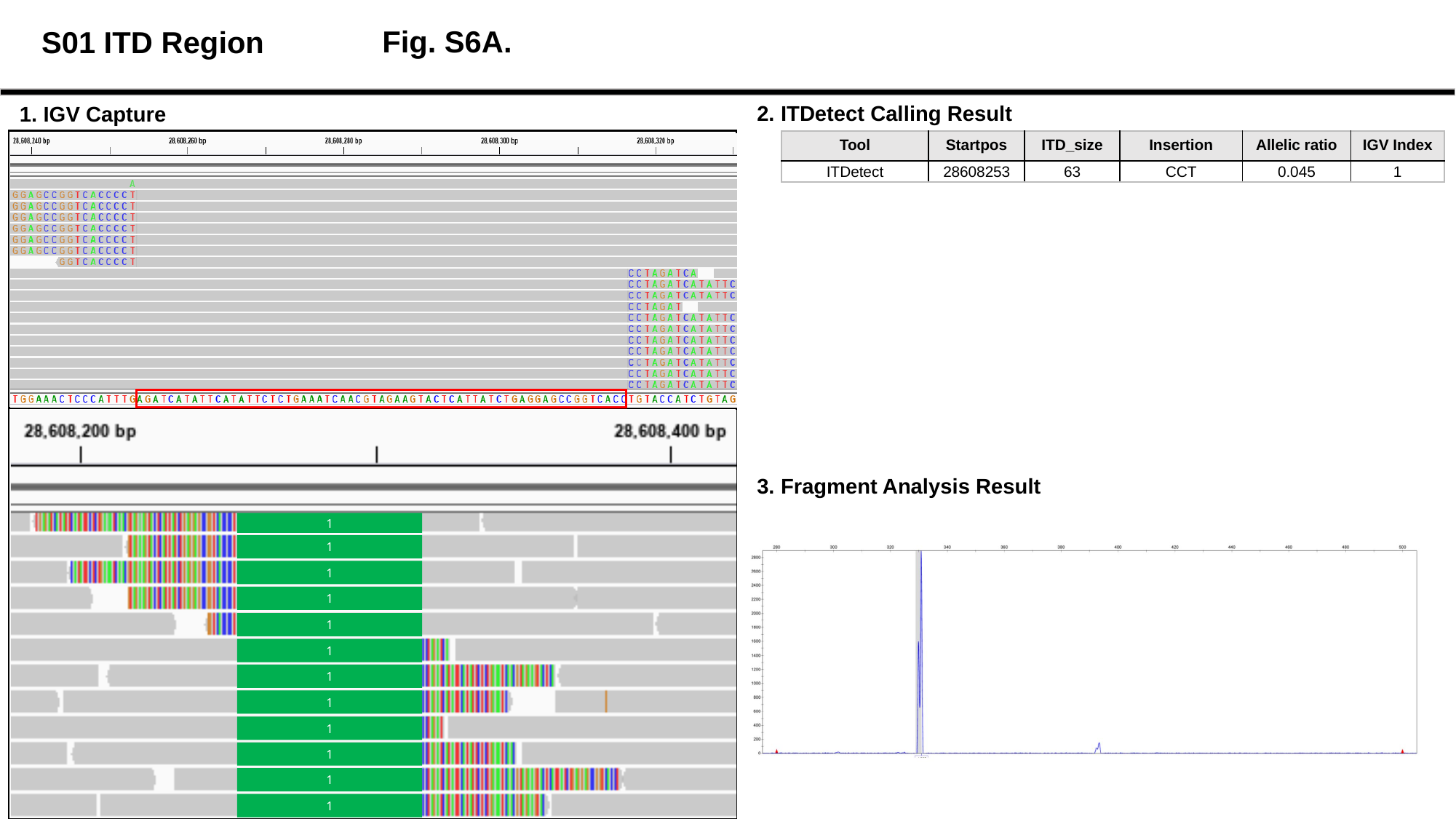

Fig. S6A.
S01 ITD Region
2. ITDetect Calling Result
1. IGV Capture
| Tool | Startpos | ITD\_size | Insertion | Allelic ratio | IGV Index |
| --- | --- | --- | --- | --- | --- |
| ITDetect | 28608253 | 63 | CCT | 0.045 | 1 |
3. Fragment Analysis Result
1
1
1
1
1
1
1
1
1
1
1
1

## Slide 17
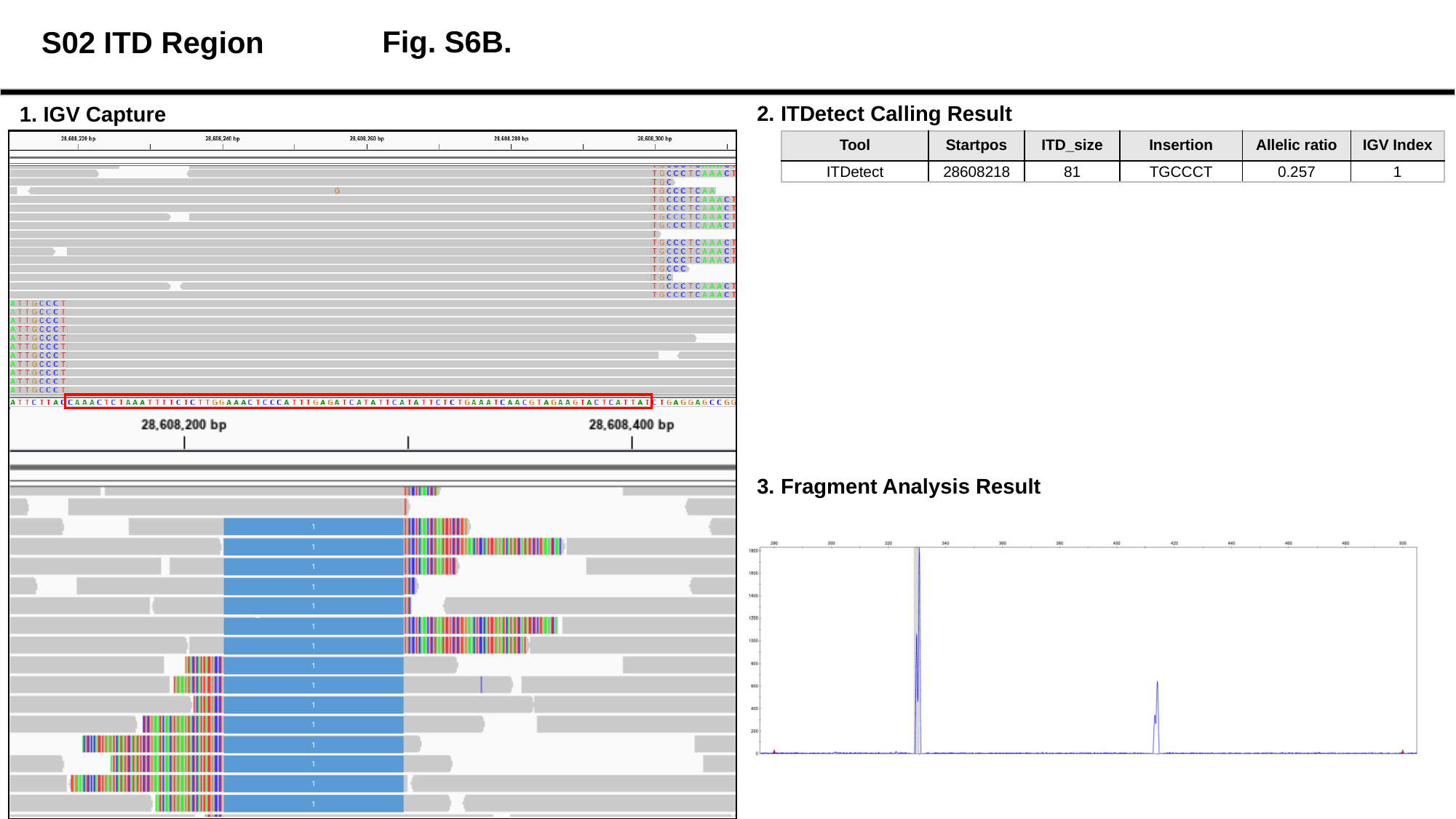

Fig. S6B.
S02 ITD Region
2. ITDetect Calling Result
1. IGV Capture
| Tool | Startpos | ITD\_size | Insertion | Allelic ratio | IGV Index |
| --- | --- | --- | --- | --- | --- |
| ITDetect | 28608218 | 81 | TGCCCT | 0.257 | 1 |
3. Fragment Analysis Result
1
1
1
1
1
1
1
1
1
1
1
1
1
1
1

## Slide 18
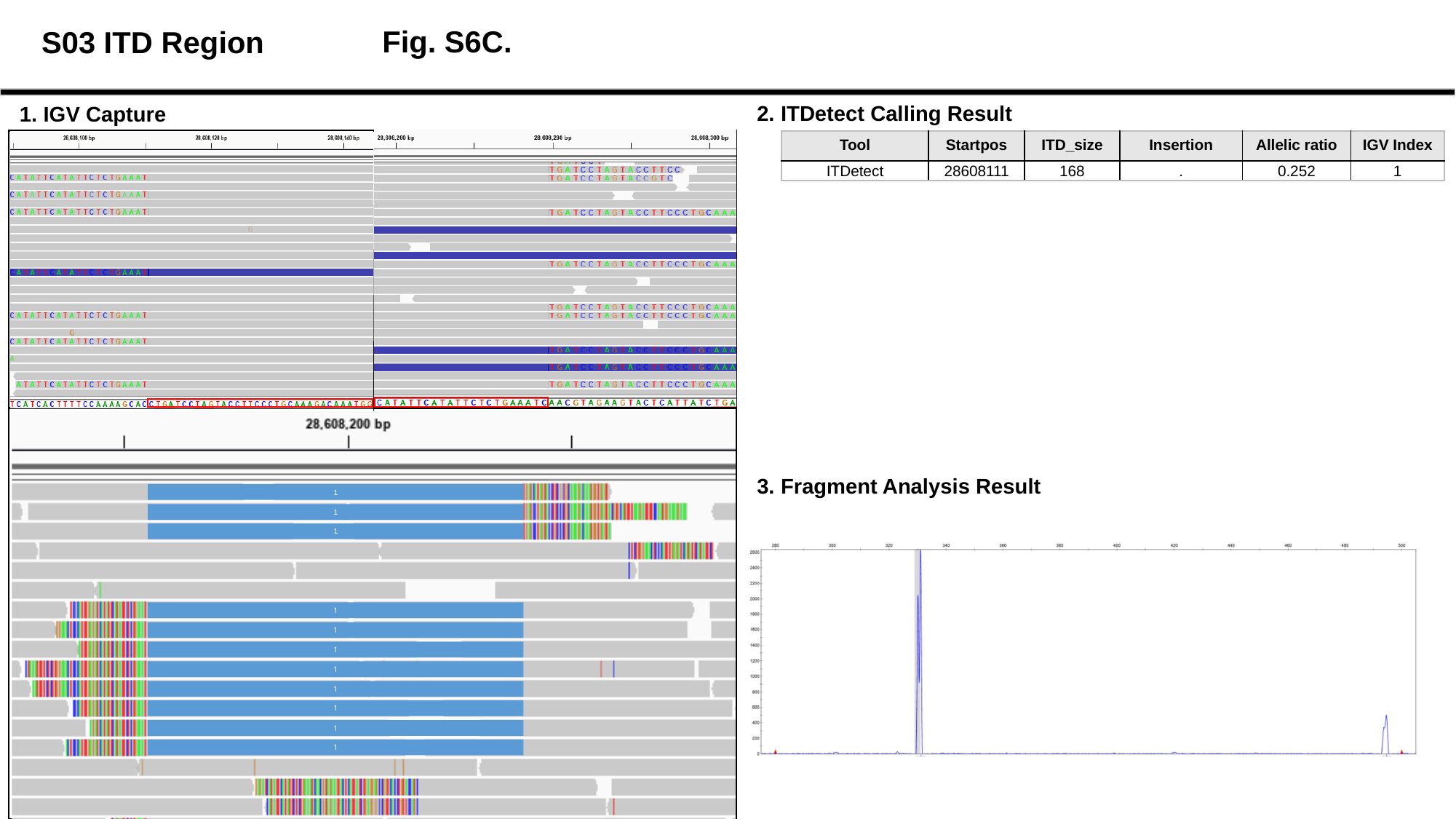

Fig. S6C.
S03 ITD Region
2. ITDetect Calling Result
1. IGV Capture
| Tool | Startpos | ITD\_size | Insertion | Allelic ratio | IGV Index |
| --- | --- | --- | --- | --- | --- |
| ITDetect | 28608111 | 168 | . | 0.252 | 1 |
3. Fragment Analysis Result
1
1
1
1
1
1
1
1
1
1
1

## Slide 19
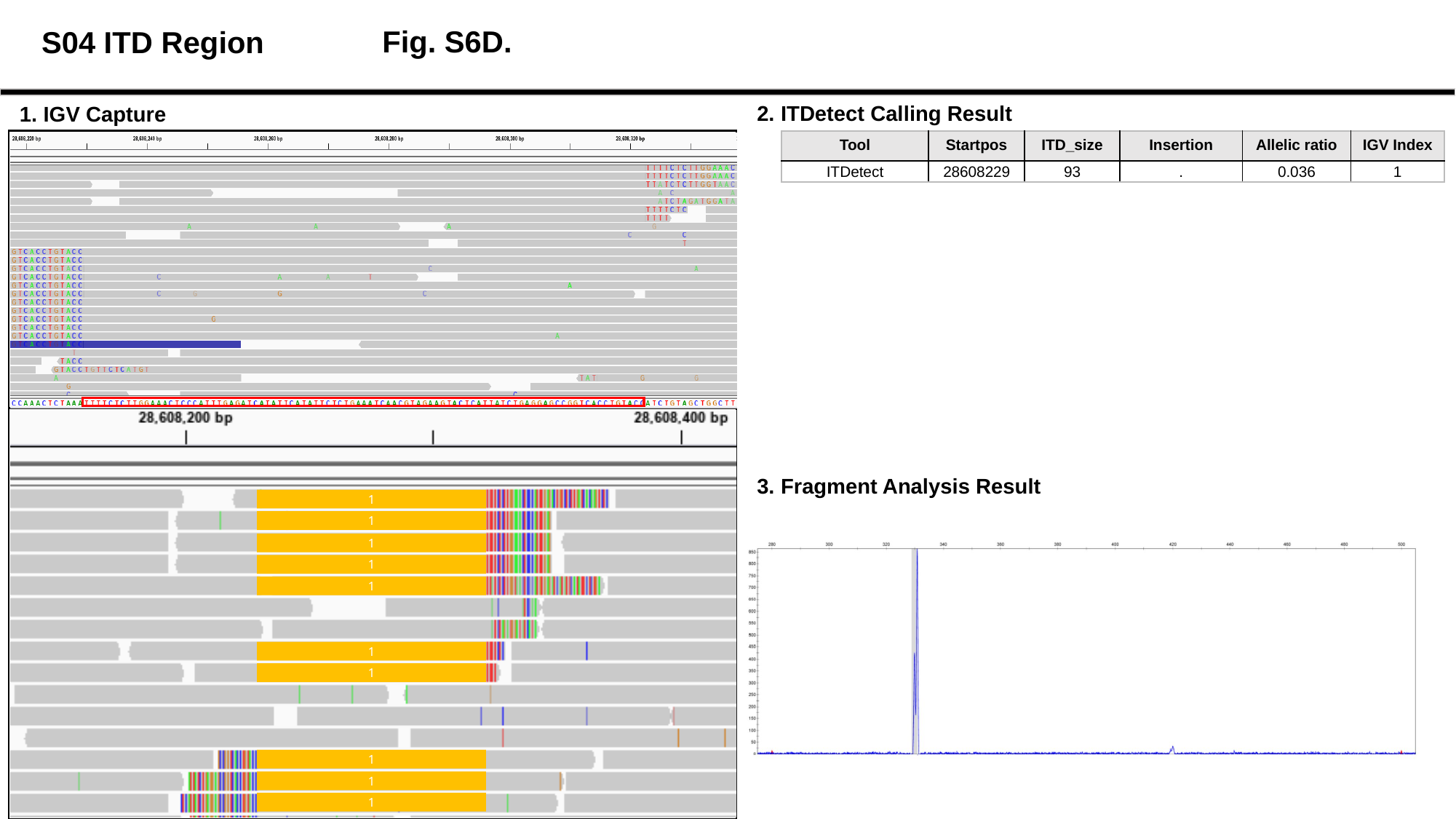

Fig. S6D.
S04 ITD Region
2. ITDetect Calling Result
1. IGV Capture
| Tool | Startpos | ITD\_size | Insertion | Allelic ratio | IGV Index |
| --- | --- | --- | --- | --- | --- |
| ITDetect | 28608229 | 93 | . | 0.036 | 1 |
3. Fragment Analysis Result
1
1
1
1
1
1
1
1
1
1

## Slide 20
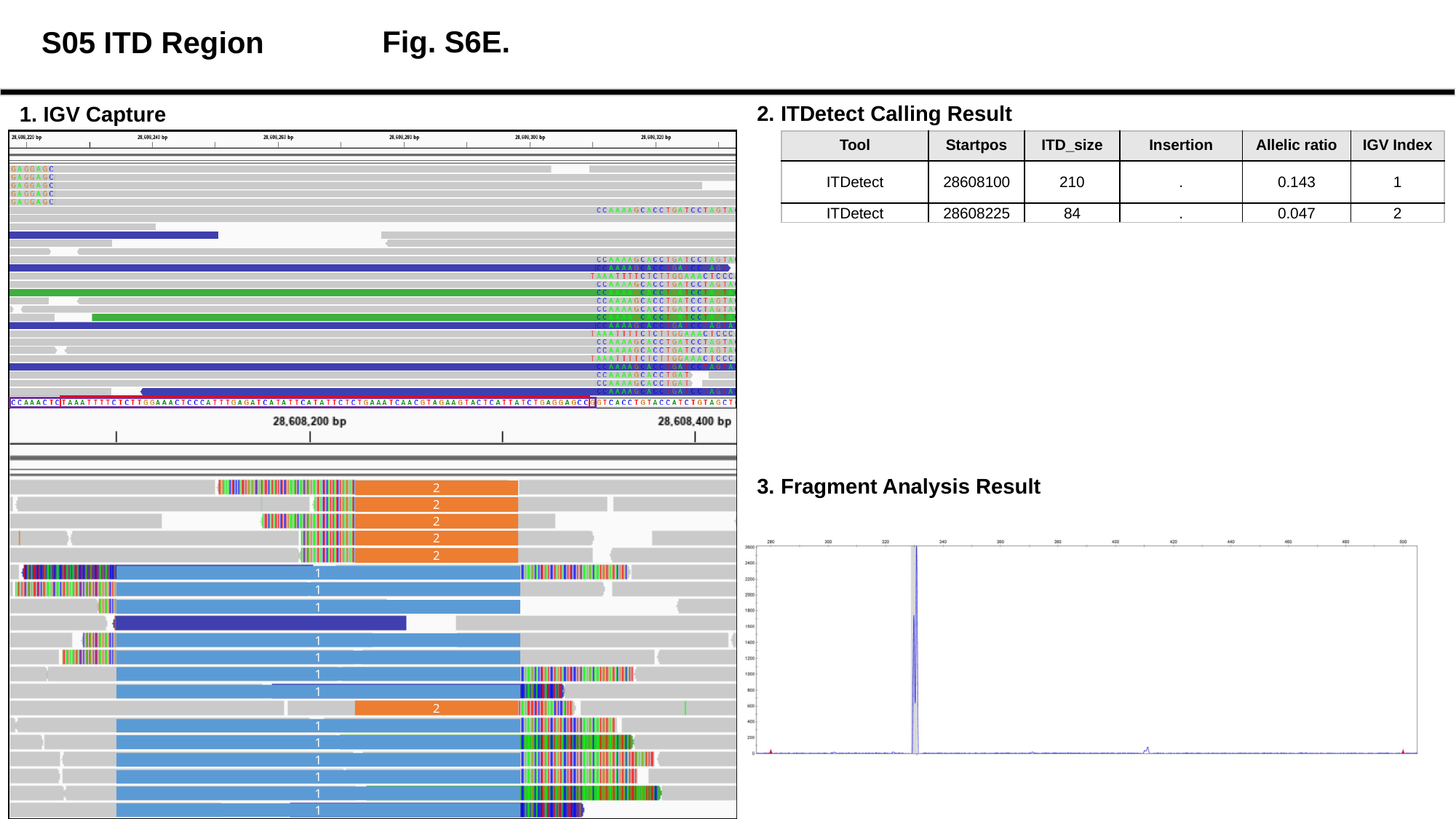

Fig. S6E.
S05 ITD Region
2. ITDetect Calling Result
1. IGV Capture
| Tool | Startpos | ITD\_size | Insertion | Allelic ratio | IGV Index |
| --- | --- | --- | --- | --- | --- |
| ITDetect | 28608100 | 210 | . | 0.143 | 1 |
| ITDetect | 28608225 | 84 | . | 0.047 | 2 |
3. Fragment Analysis Result
2
2
2
2
2
1
1
1
1
1
1
1
2
1
1
1
1
1
1

## Slide 21
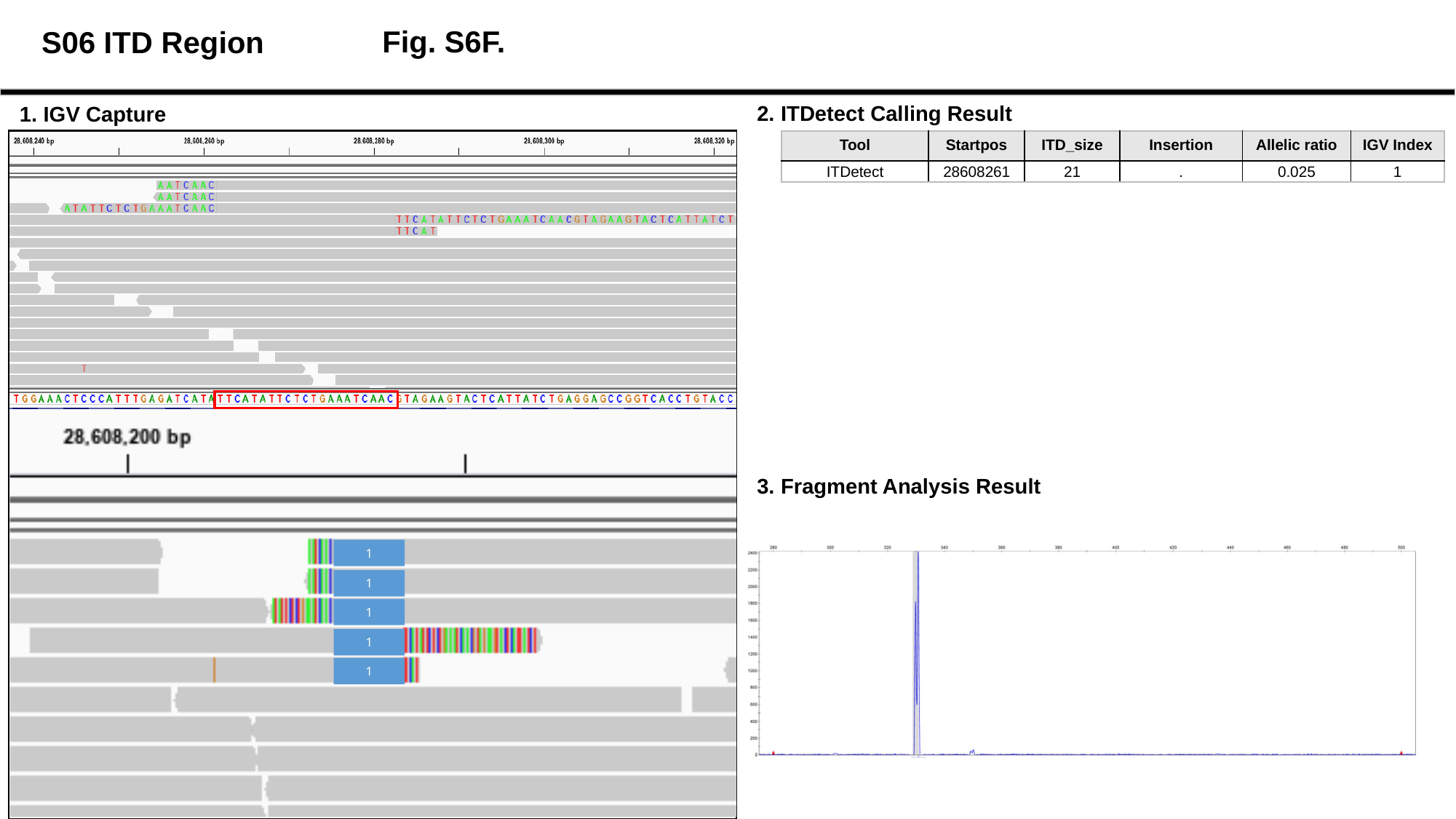

Fig. S6F.
S06 ITD Region
2. ITDetect Calling Result
1. IGV Capture
| Tool | Startpos | ITD\_size | Insertion | Allelic ratio | IGV Index |
| --- | --- | --- | --- | --- | --- |
| ITDetect | 28608261 | 21 | . | 0.025 | 1 |
3. Fragment Analysis Result
1
1
1
1
1

## Slide 22
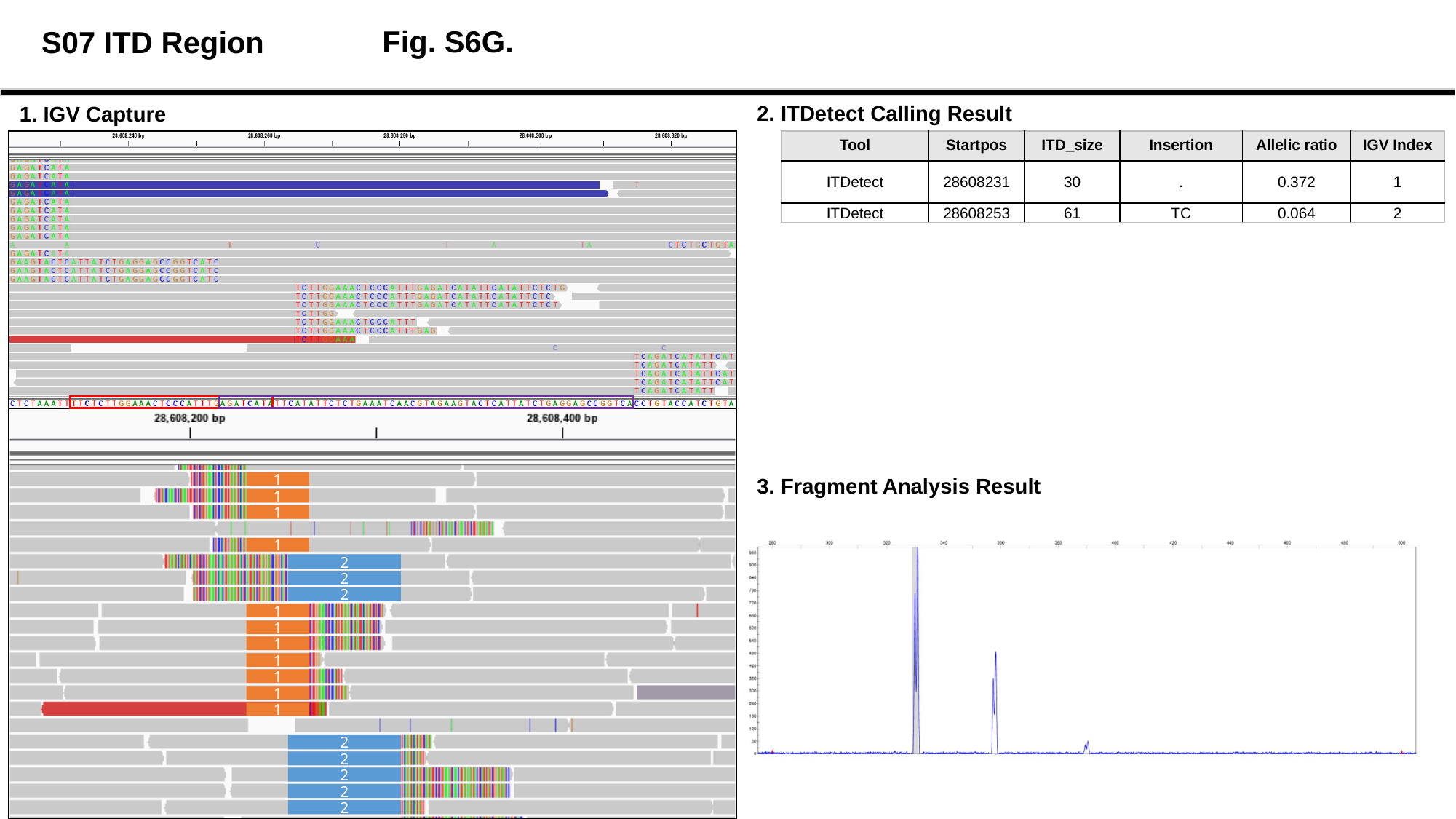

Fig. S6G.
S07 ITD Region
2. ITDetect Calling Result
1. IGV Capture
| Tool | Startpos | ITD\_size | Insertion | Allelic ratio | IGV Index |
| --- | --- | --- | --- | --- | --- |
| ITDetect | 28608231 | 30 | . | 0.372 | 1 |
| ITDetect | 28608253 | 61 | TC | 0.064 | 2 |
3. Fragment Analysis Result
1
1
1
1
2
2
2
1
1
1
1
1
1
1
2
2
2
2
2

## Slide 23
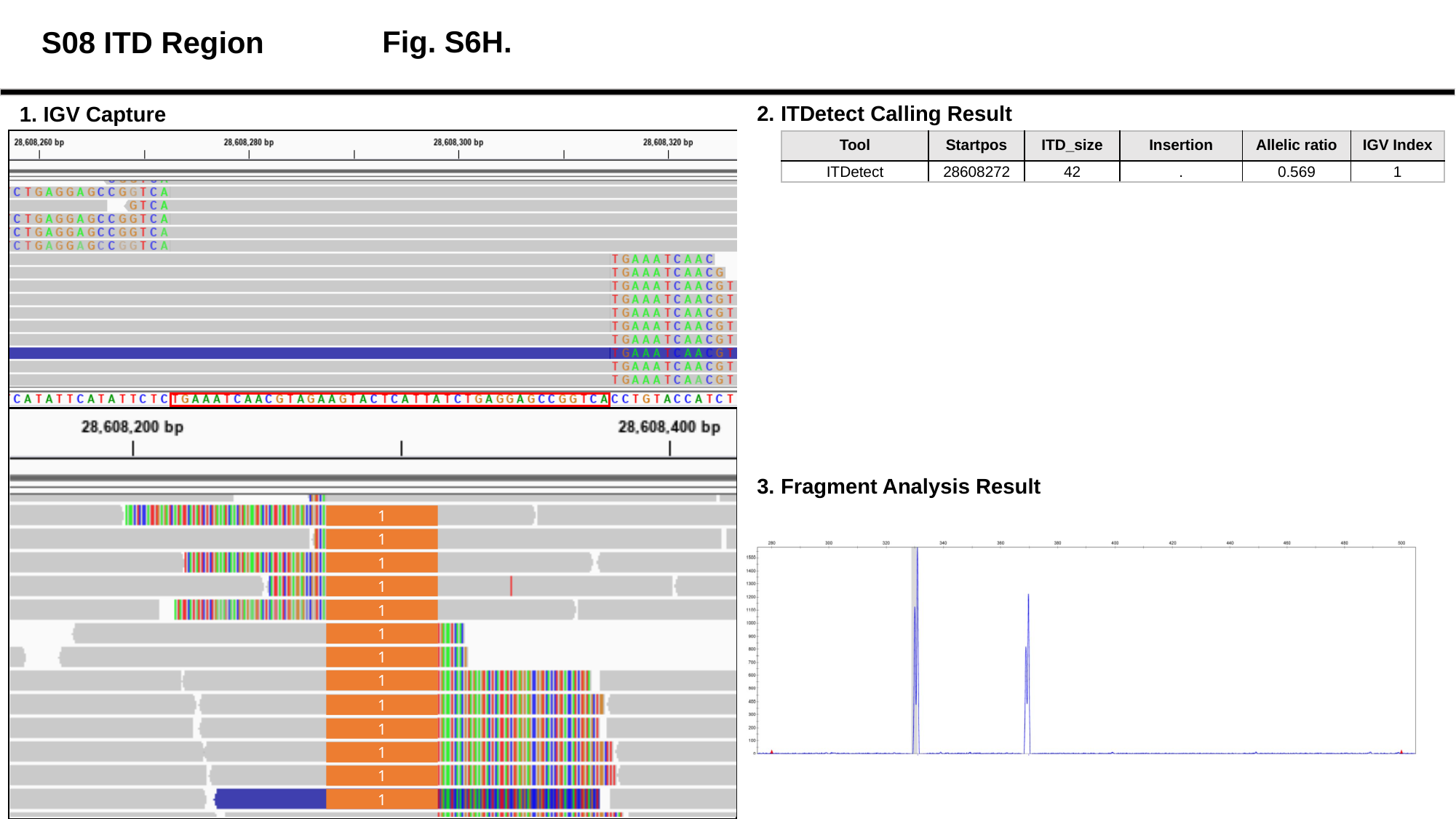

Fig. S6H.
S08 ITD Region
2. ITDetect Calling Result
1. IGV Capture
| Tool | Startpos | ITD\_size | Insertion | Allelic ratio | IGV Index |
| --- | --- | --- | --- | --- | --- |
| ITDetect | 28608272 | 42 | . | 0.569 | 1 |
3. Fragment Analysis Result
1
1
1
1
1
1
1
1
1
1
1
1
1

## Slide 24
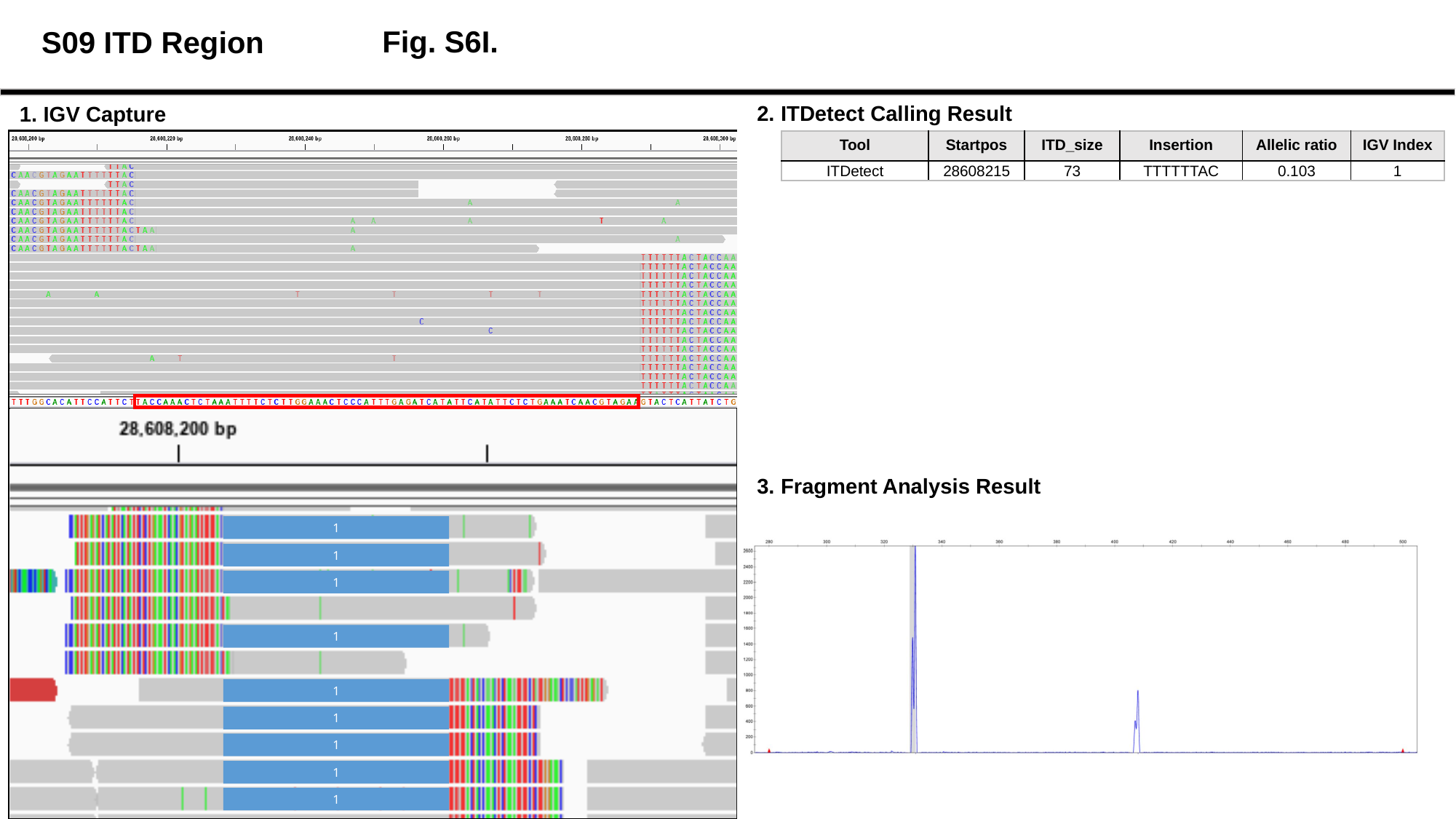

Fig. S6I.
S09 ITD Region
2. ITDetect Calling Result
1. IGV Capture
| Tool | Startpos | ITD\_size | Insertion | Allelic ratio | IGV Index |
| --- | --- | --- | --- | --- | --- |
| ITDetect | 28608215 | 73 | TTTTTTAC | 0.103 | 1 |
3. Fragment Analysis Result
1
1
1
1
1
1
1
1
1

## Slide 25
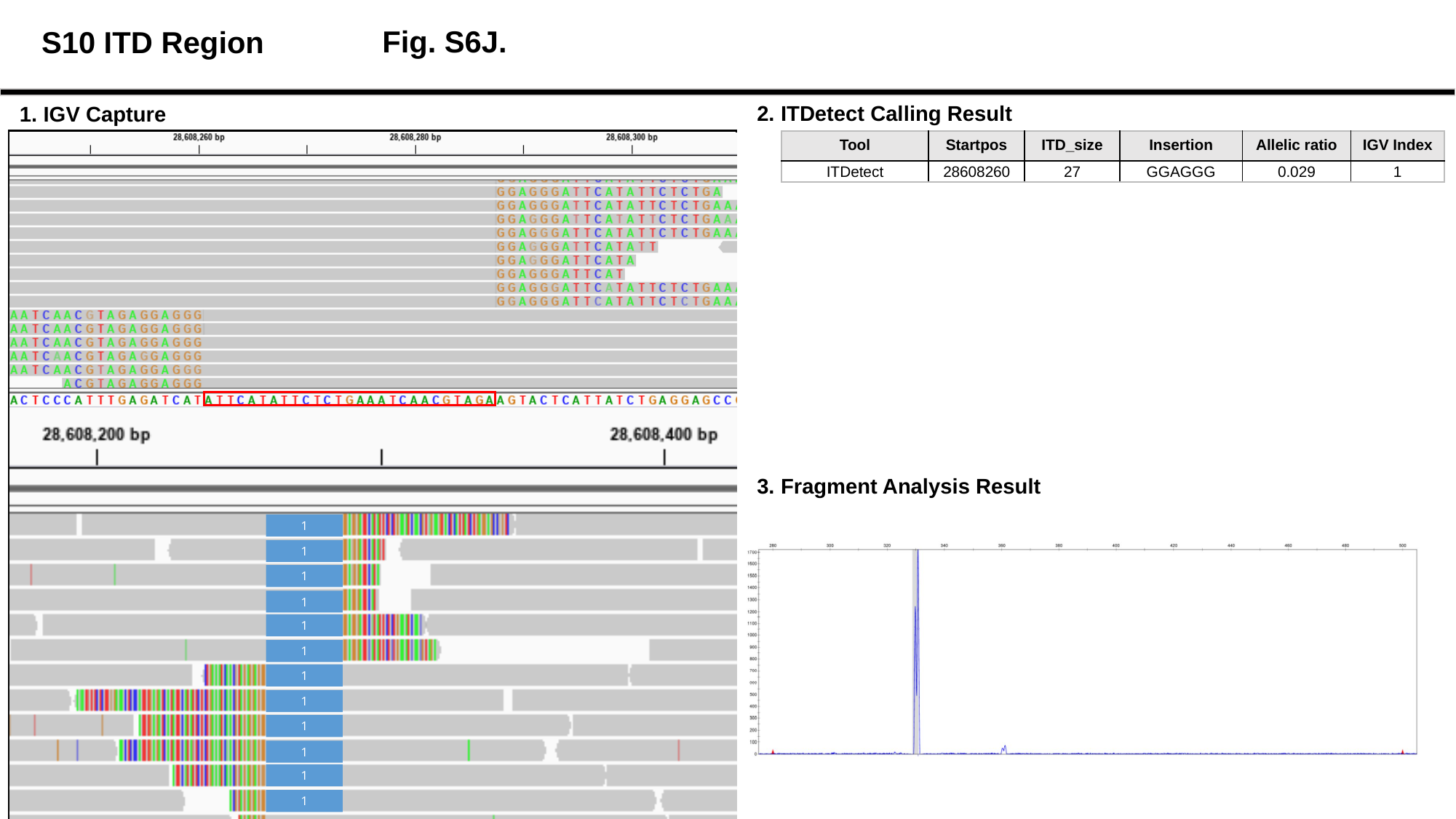

Fig. S6J.
S10 ITD Region
2. ITDetect Calling Result
1. IGV Capture
| Tool | Startpos | ITD\_size | Insertion | Allelic ratio | IGV Index |
| --- | --- | --- | --- | --- | --- |
| ITDetect | 28608260 | 27 | GGAGGG | 0.029 | 1 |
3. Fragment Analysis Result
1
1
1
1
1
1
1
1
1
1
1
1

## Slide 26
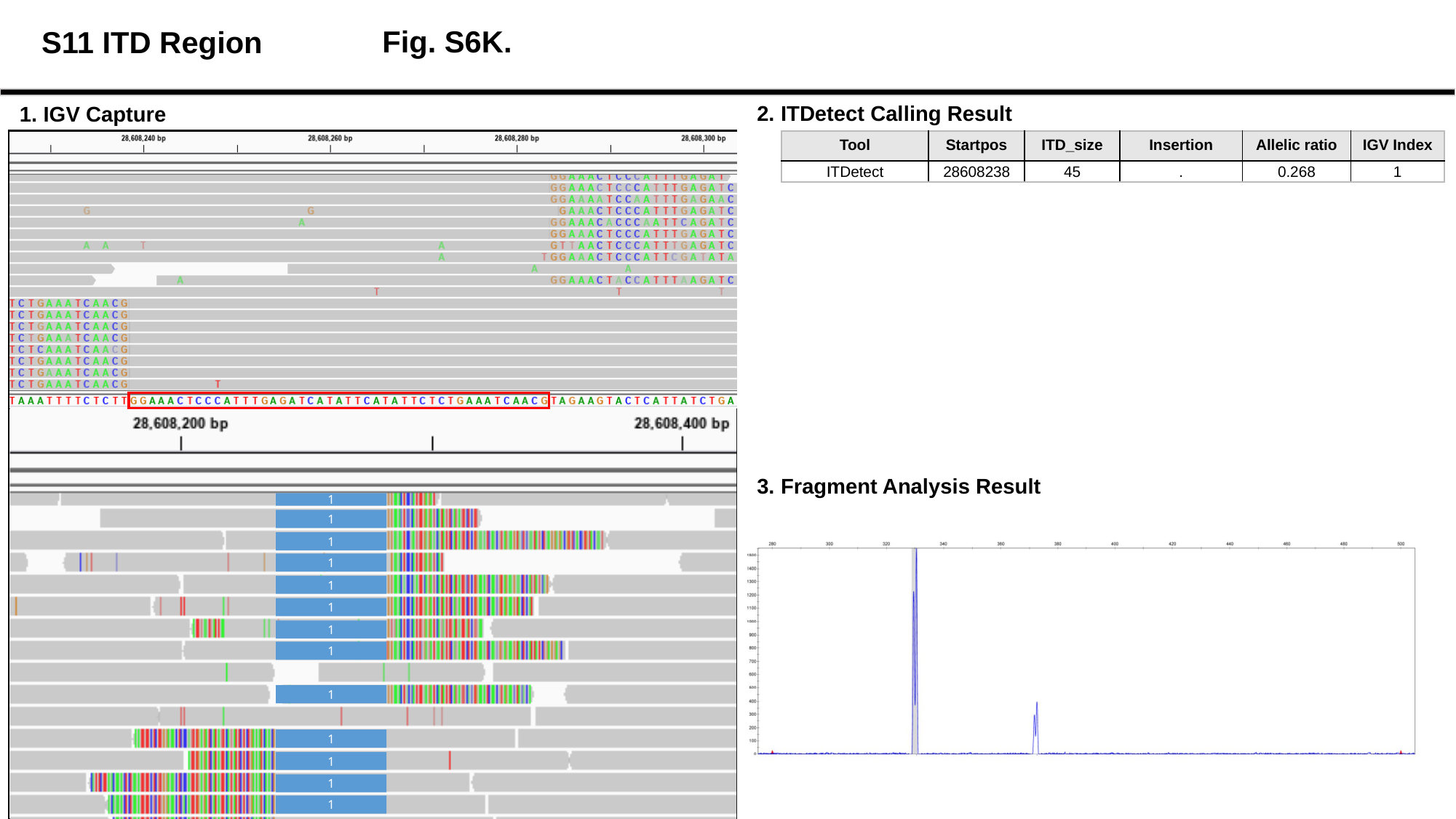

Fig. S6K.
S11 ITD Region
2. ITDetect Calling Result
1. IGV Capture
| Tool | Startpos | ITD\_size | Insertion | Allelic ratio | IGV Index |
| --- | --- | --- | --- | --- | --- |
| ITDetect | 28608238 | 45 | . | 0.268 | 1 |
3. Fragment Analysis Result
1
1
1
1
1
1
1
1
1
1
1
1
1

## Slide 27
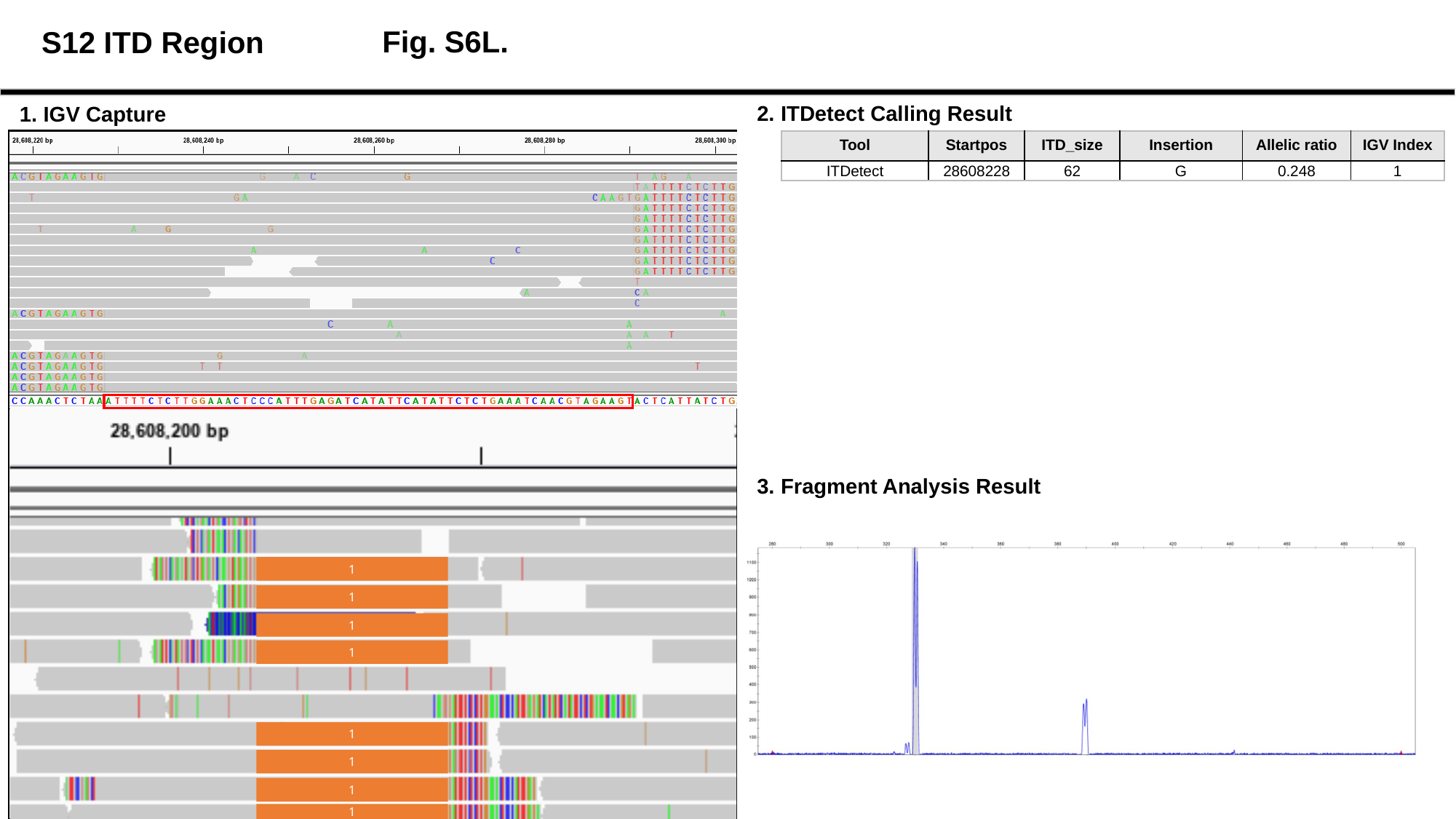

Fig. S6L.
S12 ITD Region
2. ITDetect Calling Result
1. IGV Capture
| Tool | Startpos | ITD\_size | Insertion | Allelic ratio | IGV Index |
| --- | --- | --- | --- | --- | --- |
| ITDetect | 28608228 | 62 | G | 0.248 | 1 |
3. Fragment Analysis Result
1
1
1
1
1
1
1
1

## Slide 28
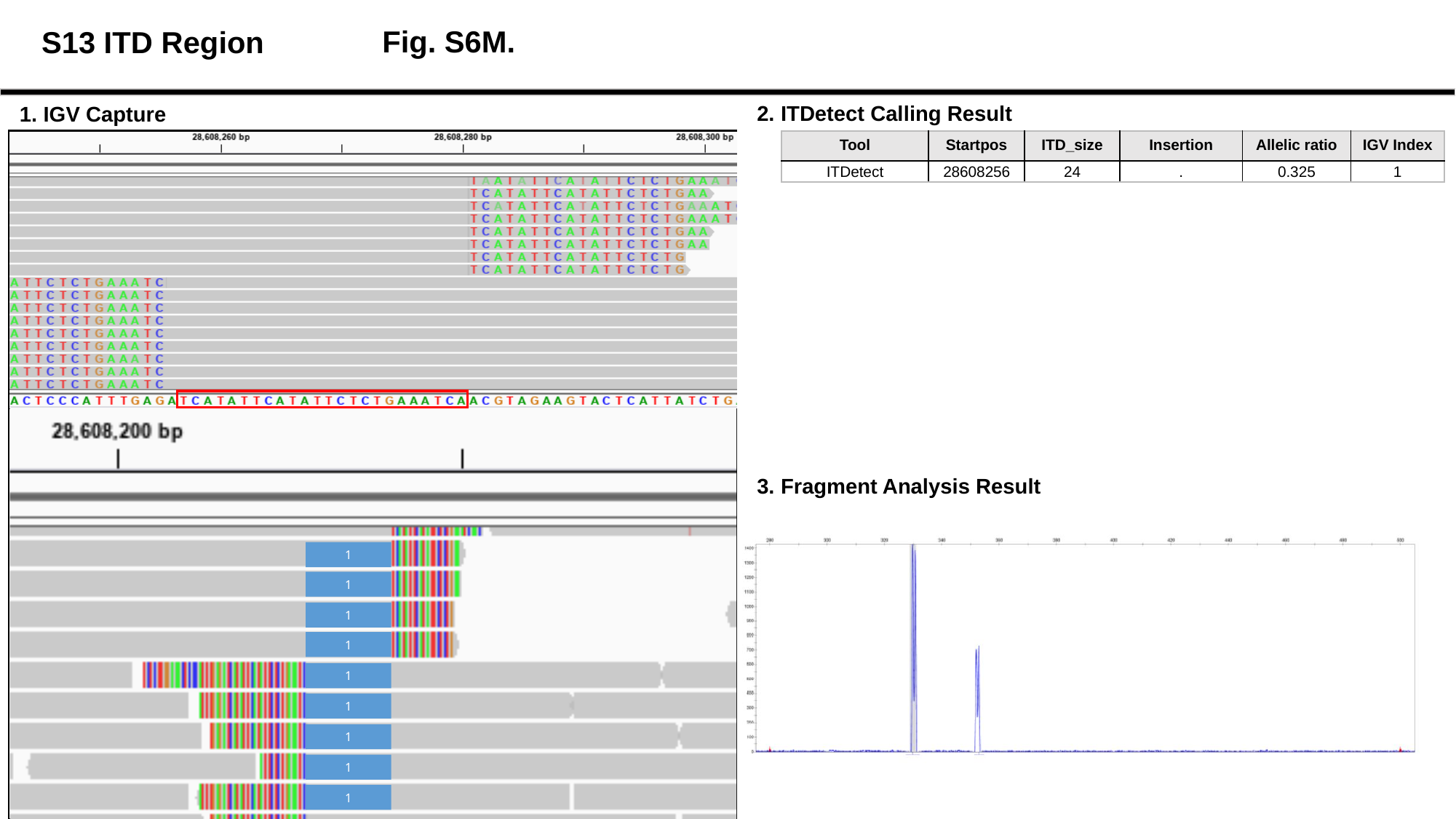

Fig. S6M.
S13 ITD Region
2. ITDetect Calling Result
1. IGV Capture
| Tool | Startpos | ITD\_size | Insertion | Allelic ratio | IGV Index |
| --- | --- | --- | --- | --- | --- |
| ITDetect | 28608256 | 24 | . | 0.325 | 1 |
3. Fragment Analysis Result
1
1
1
1
1
1
1
1
1

## Slide 29
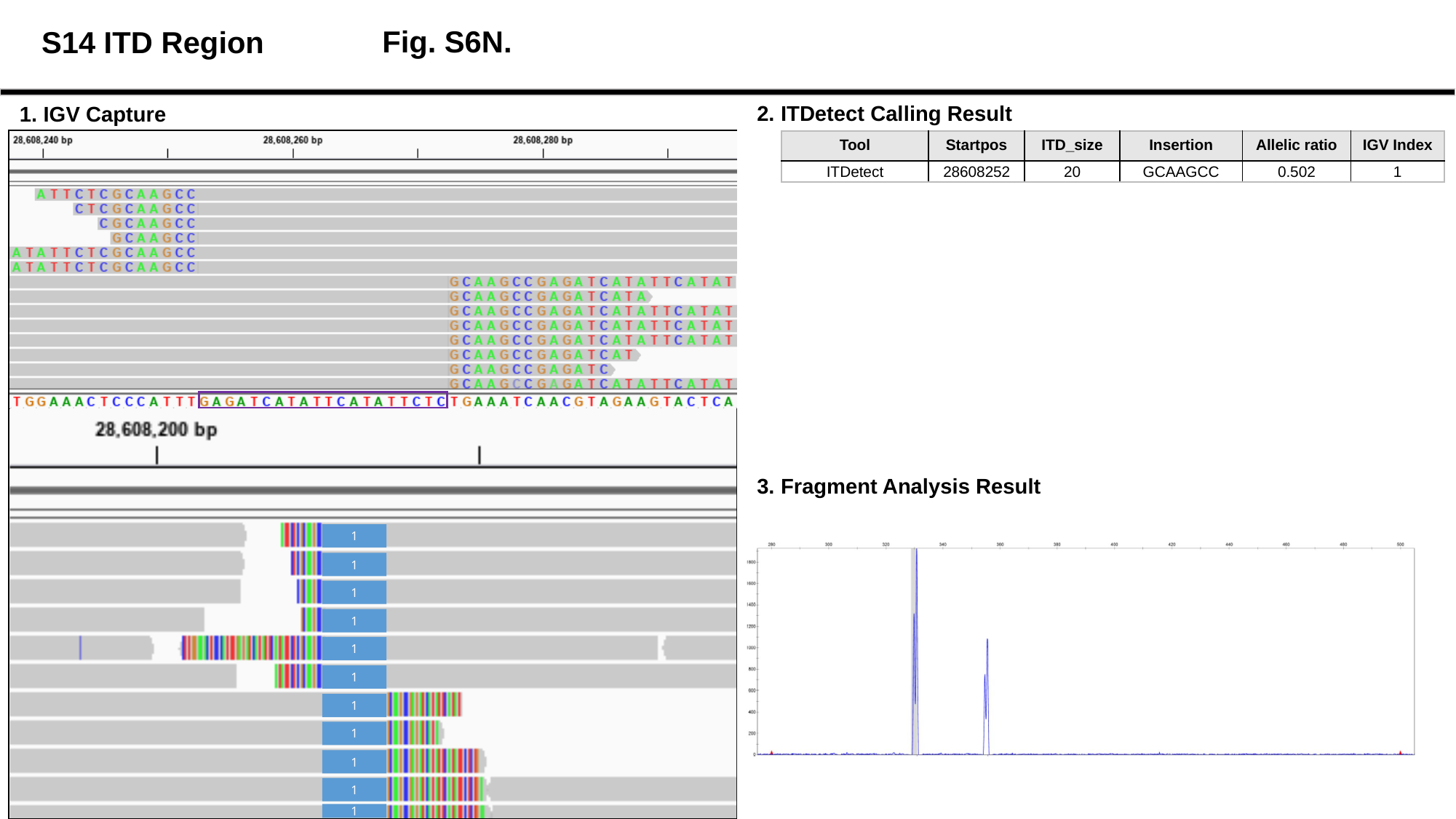

Fig. S6N.
S14 ITD Region
2. ITDetect Calling Result
1. IGV Capture
| Tool | Startpos | ITD\_size | Insertion | Allelic ratio | IGV Index |
| --- | --- | --- | --- | --- | --- |
| ITDetect | 28608252 | 20 | GCAAGCC | 0.502 | 1 |
3. Fragment Analysis Result
1
1
1
1
1
1
1
1
1
1
1

## Slide 30
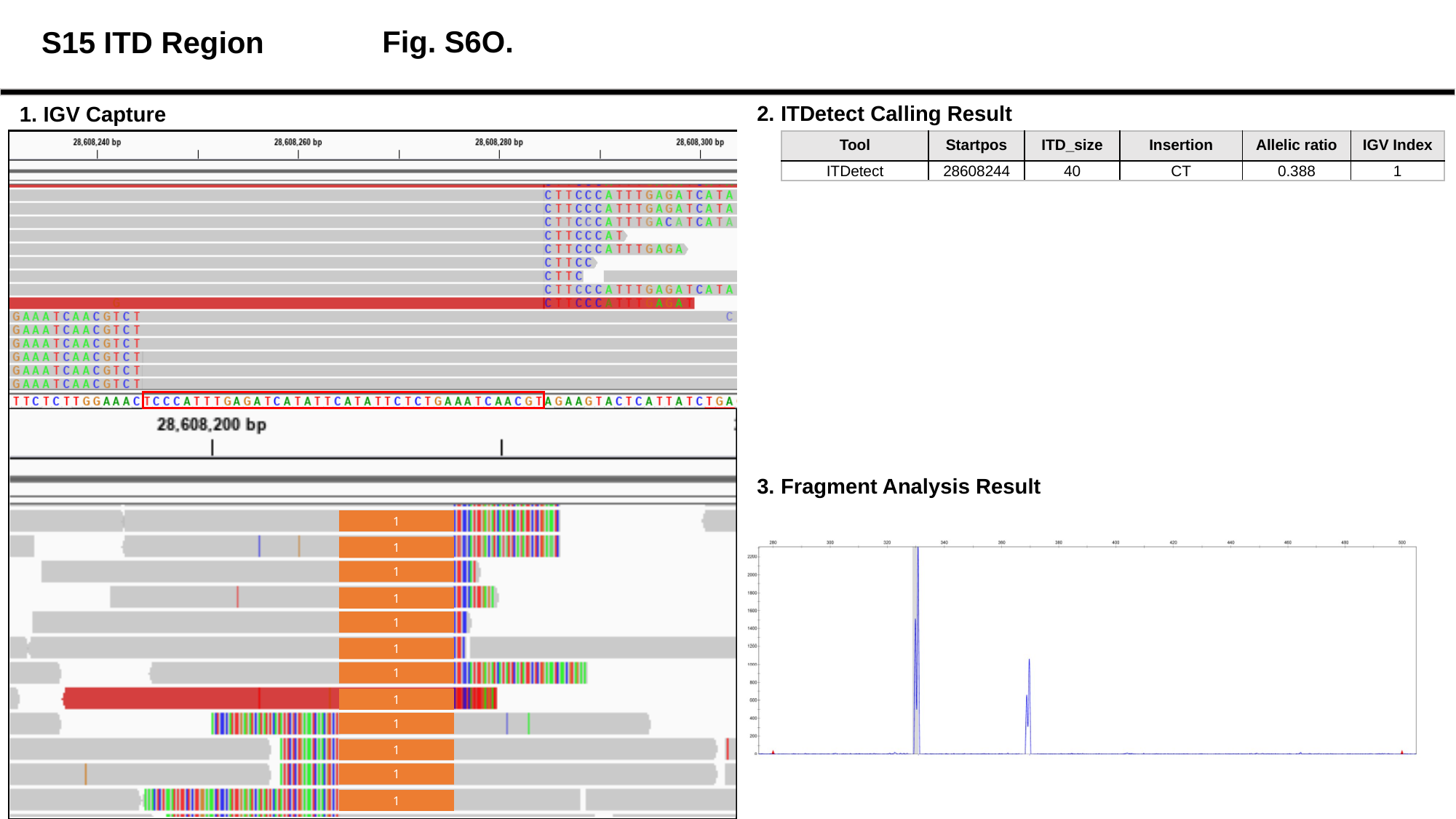

Fig. S6O.
S15 ITD Region
2. ITDetect Calling Result
1. IGV Capture
| Tool | Startpos | ITD\_size | Insertion | Allelic ratio | IGV Index |
| --- | --- | --- | --- | --- | --- |
| ITDetect | 28608244 | 40 | CT | 0.388 | 1 |
3. Fragment Analysis Result
1
1
1
1
1
1
1
1
1
1
1
1

## Slide 31
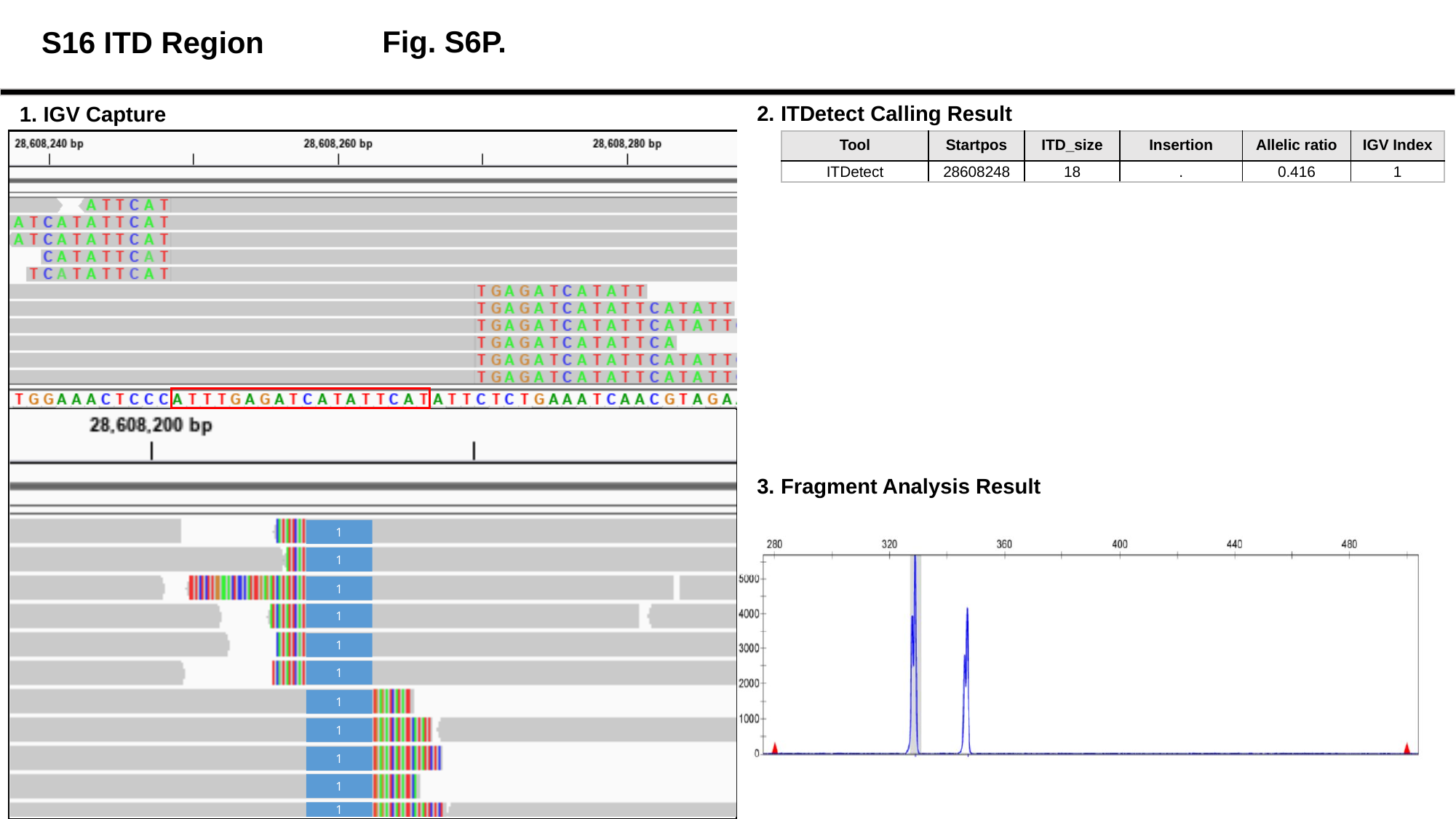

Fig. S6P.
S16 ITD Region
2. ITDetect Calling Result
1. IGV Capture
| Tool | Startpos | ITD\_size | Insertion | Allelic ratio | IGV Index |
| --- | --- | --- | --- | --- | --- |
| ITDetect | 28608248 | 18 | . | 0.416 | 1 |
3. Fragment Analysis Result
1
1
1
1
1
1
1
1
1
1
1

## Slide 32
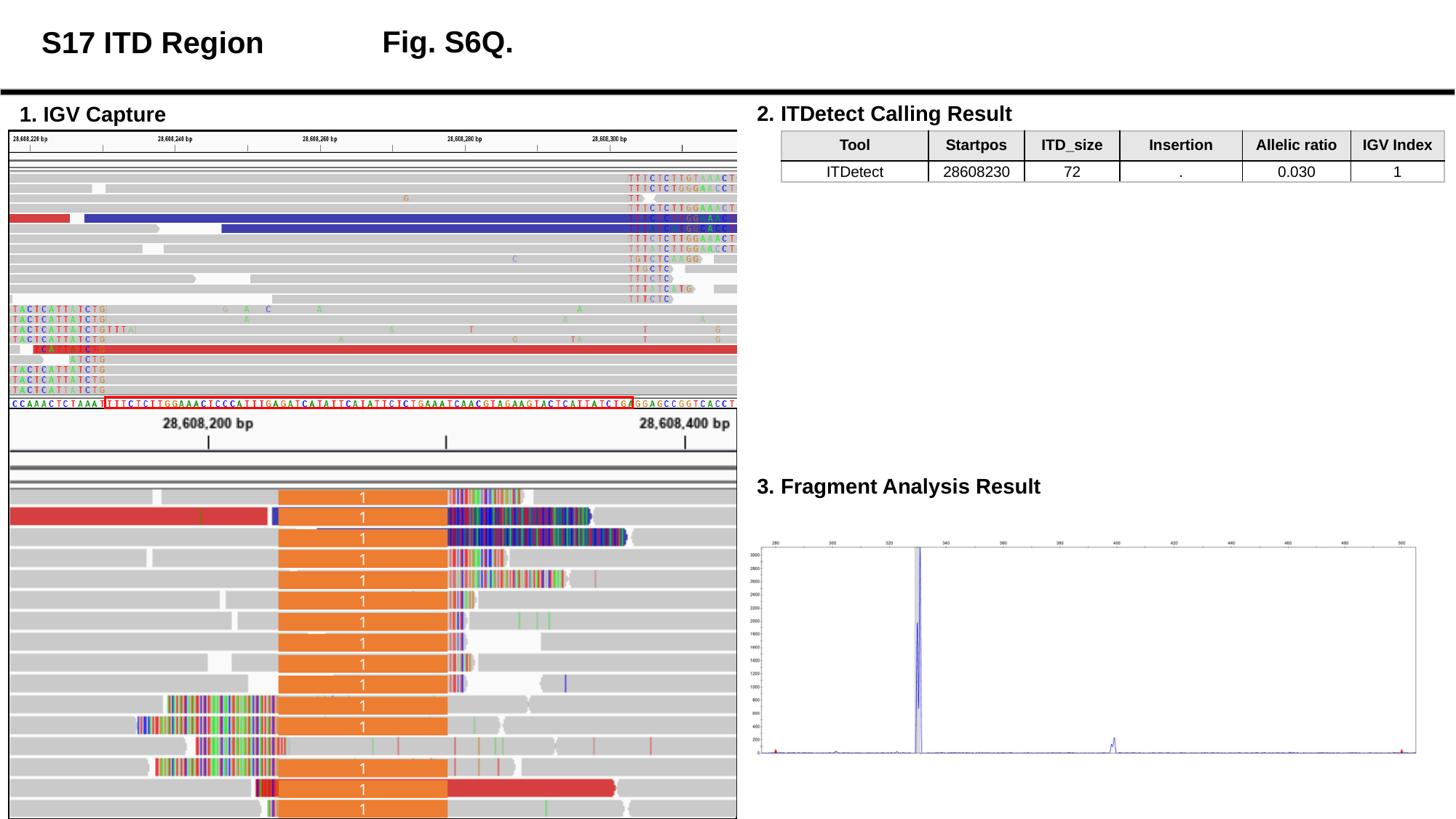

Fig. S6Q.
S17 ITD Region
2. ITDetect Calling Result
1. IGV Capture
| Tool | Startpos | ITD\_size | Insertion | Allelic ratio | IGV Index |
| --- | --- | --- | --- | --- | --- |
| ITDetect | 28608230 | 72 | . | 0.030 | 1 |
3. Fragment Analysis Result
1
1
1
1
1
1
1
1
1
1
1
1
1
1
1

## Slide 33
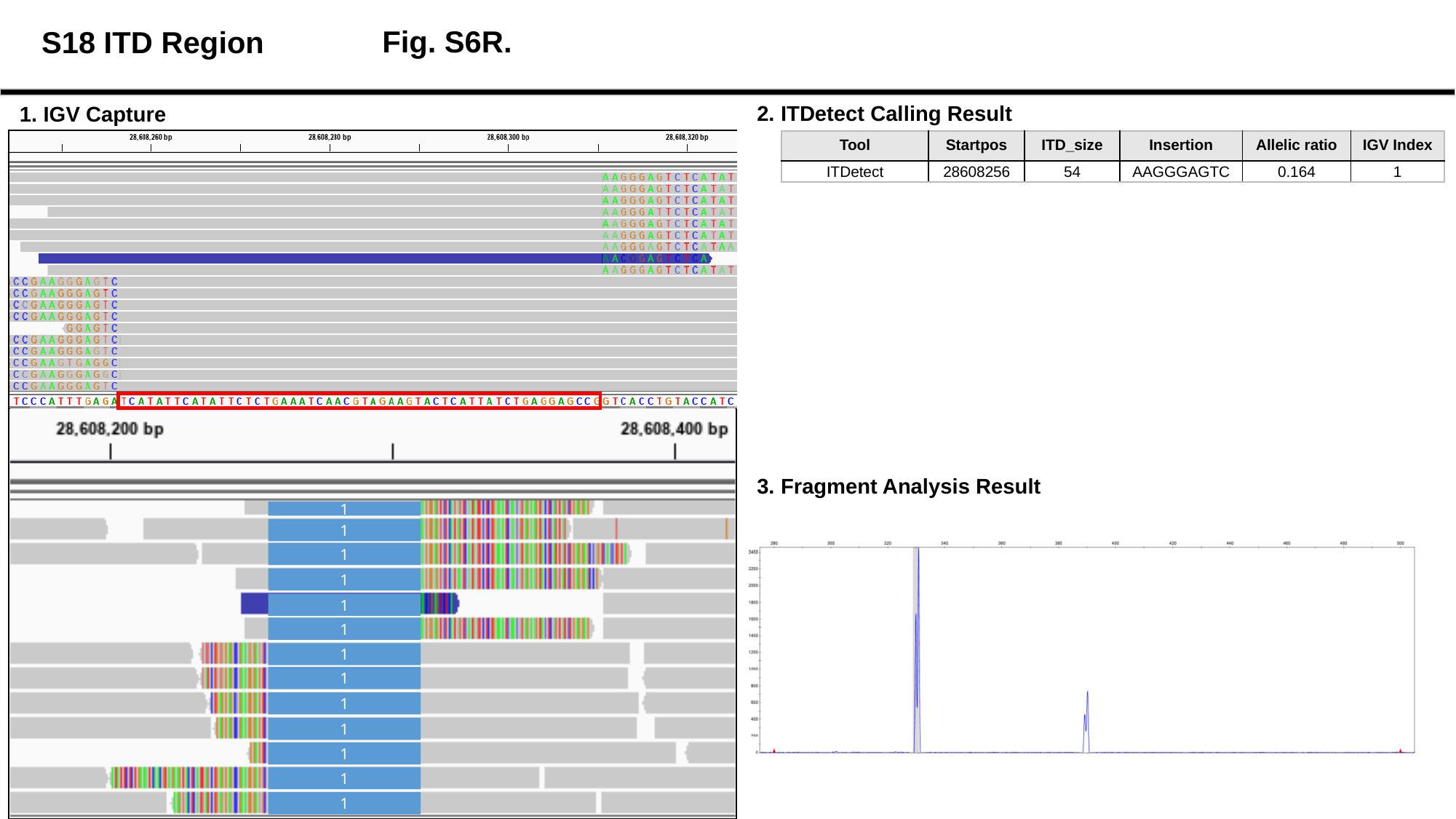

Fig. S6R.
S18 ITD Region
2. ITDetect Calling Result
1. IGV Capture
| Tool | Startpos | ITD\_size | Insertion | Allelic ratio | IGV Index |
| --- | --- | --- | --- | --- | --- |
| ITDetect | 28608256 | 54 | AAGGGAGTC | 0.164 | 1 |
3. Fragment Analysis Result
1
1
1
1
1
1
1
1
1
1
1
1
1

## Slide 34
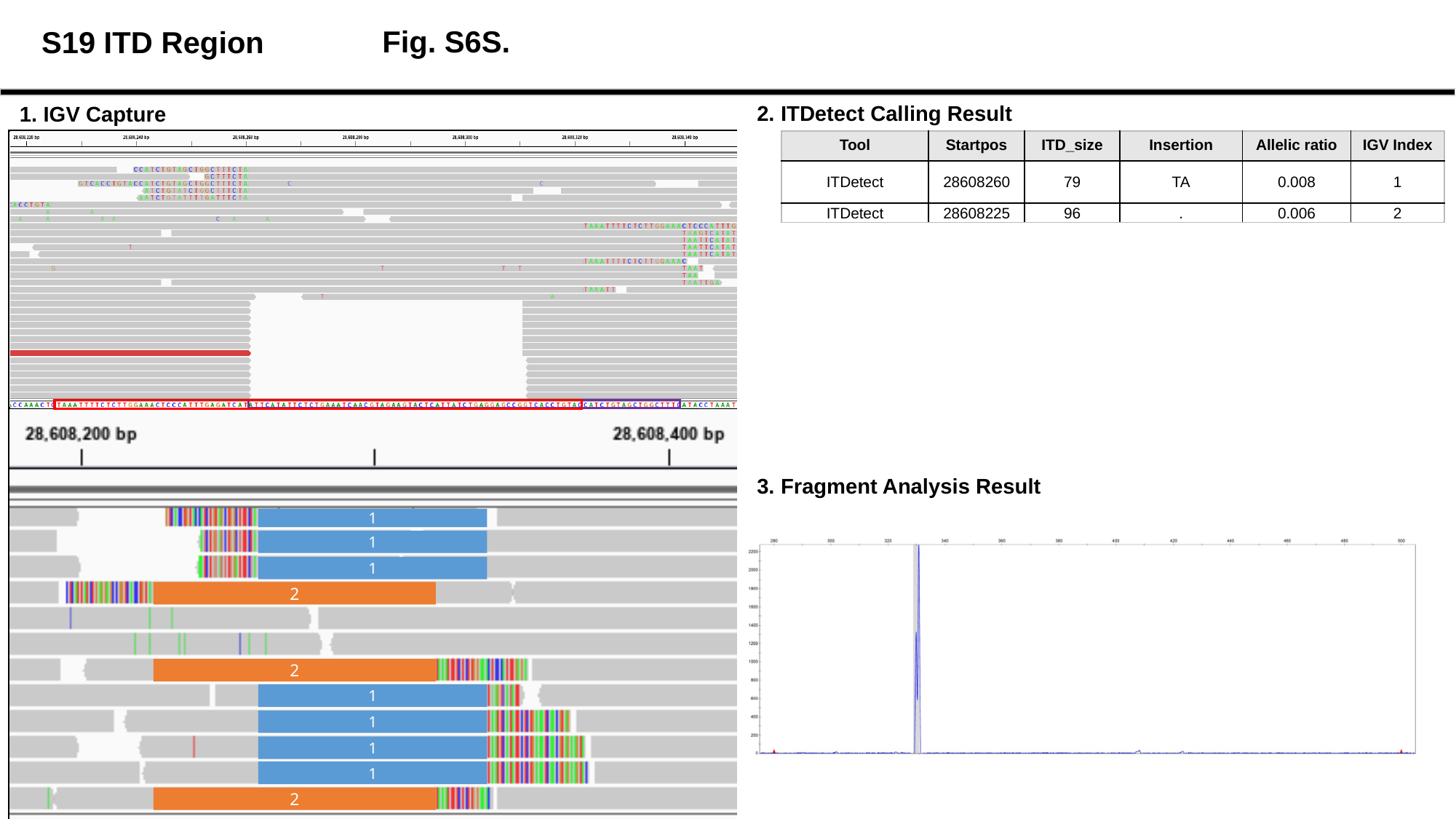

Fig. S6S.
S19 ITD Region
2. ITDetect Calling Result
1. IGV Capture
| Tool | Startpos | ITD\_size | Insertion | Allelic ratio | IGV Index |
| --- | --- | --- | --- | --- | --- |
| ITDetect | 28608260 | 79 | TA | 0.008 | 1 |
| ITDetect | 28608225 | 96 | . | 0.006 | 2 |
3. Fragment Analysis Result
1
1
1
2
2
1
1
1
1
2

## Slide 35
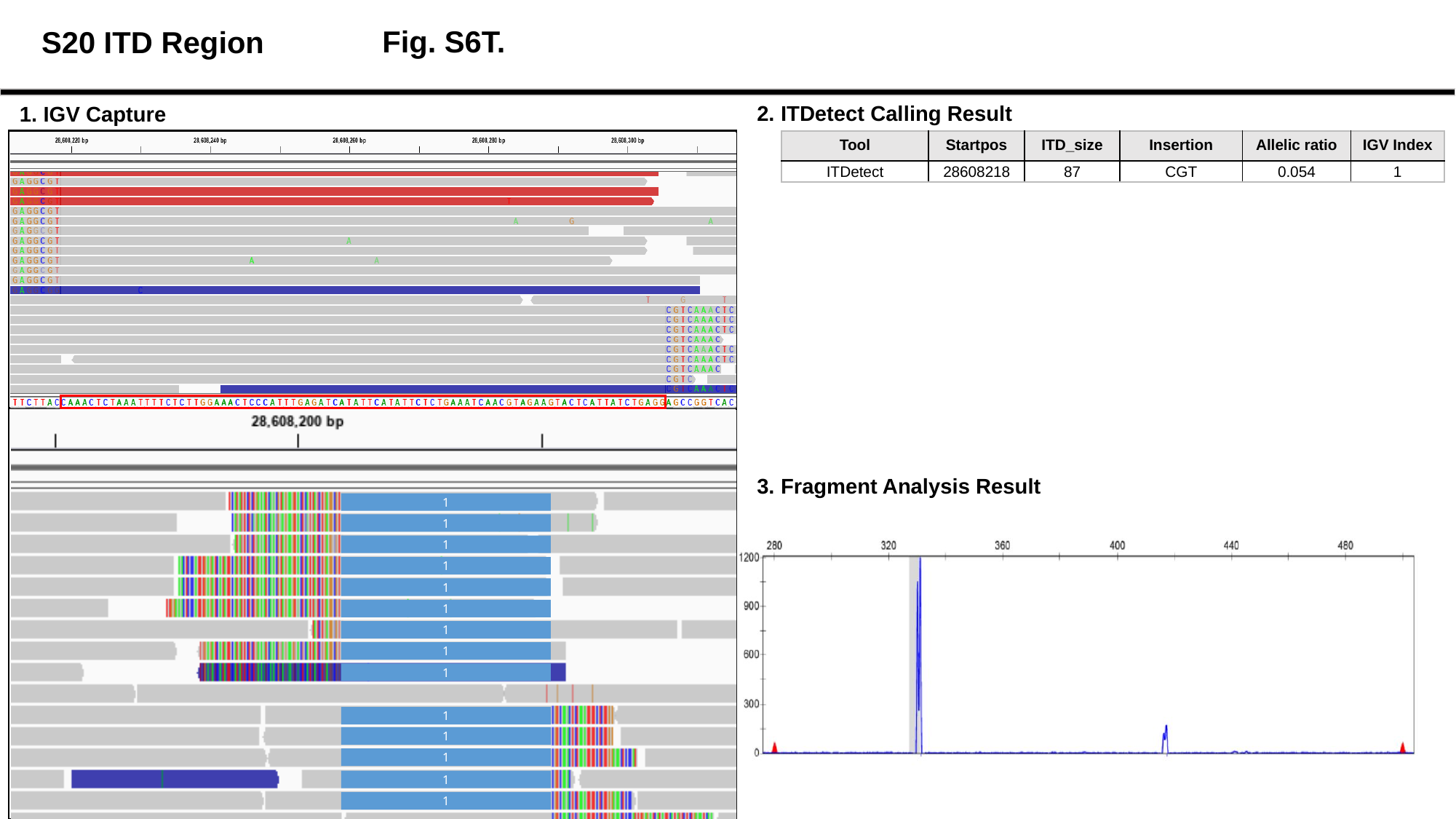

Fig. S6T.
S20 ITD Region
2. ITDetect Calling Result
1. IGV Capture
| Tool | Startpos | ITD\_size | Insertion | Allelic ratio | IGV Index |
| --- | --- | --- | --- | --- | --- |
| ITDetect | 28608218 | 87 | CGT | 0.054 | 1 |
3. Fragment Analysis Result
1
1
1
1
1
1
1
1
1
1
1
1
1
1

## Slide 36
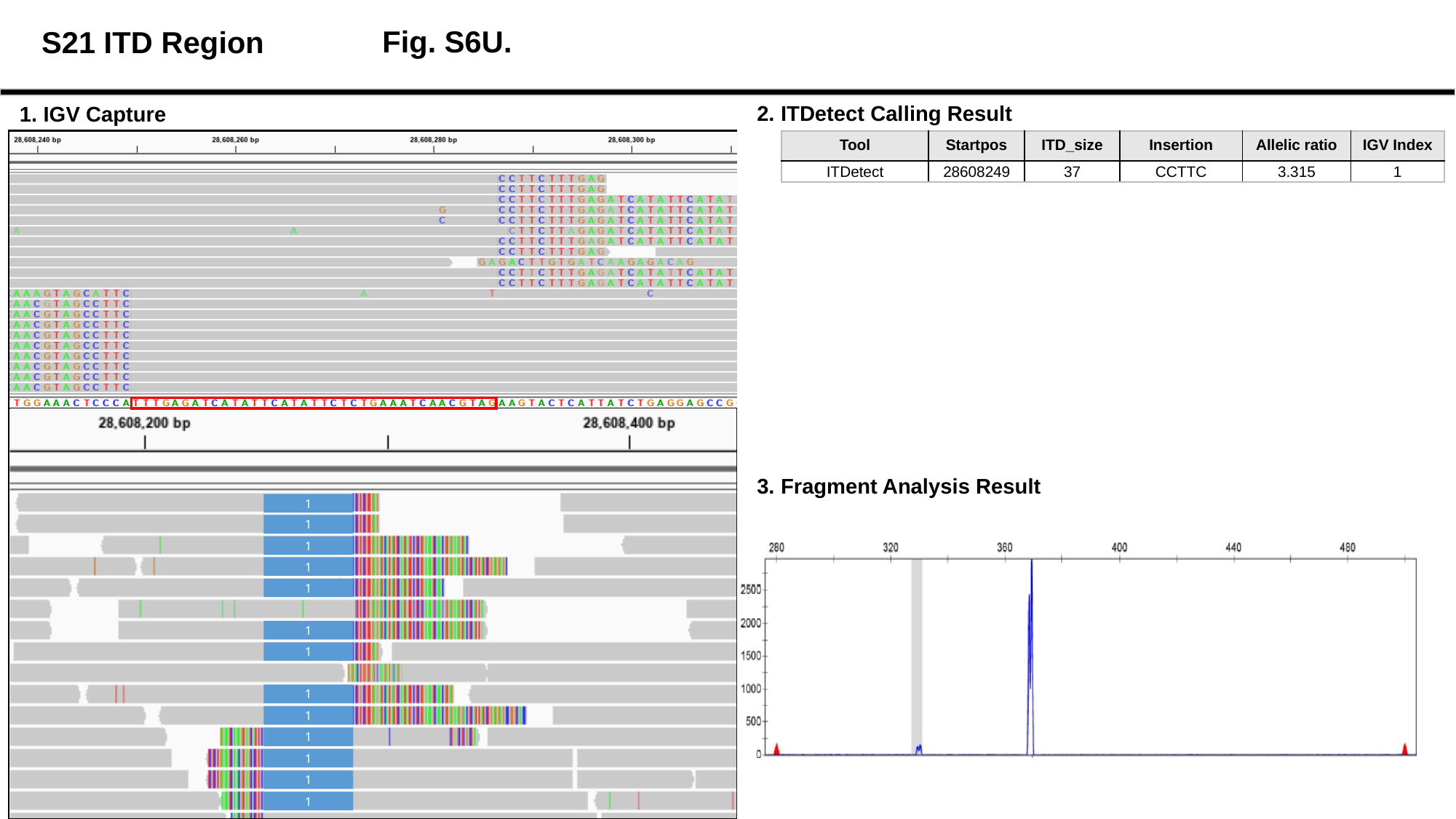

Fig. S6U.
S21 ITD Region
2. ITDetect Calling Result
1. IGV Capture
| Tool | Startpos | ITD\_size | Insertion | Allelic ratio | IGV Index |
| --- | --- | --- | --- | --- | --- |
| ITDetect | 28608249 | 37 | CCTTC | 3.315 | 1 |
3. Fragment Analysis Result
1
1
1
1
1
1
1
1
1
1
1
1
1

## Slide 37
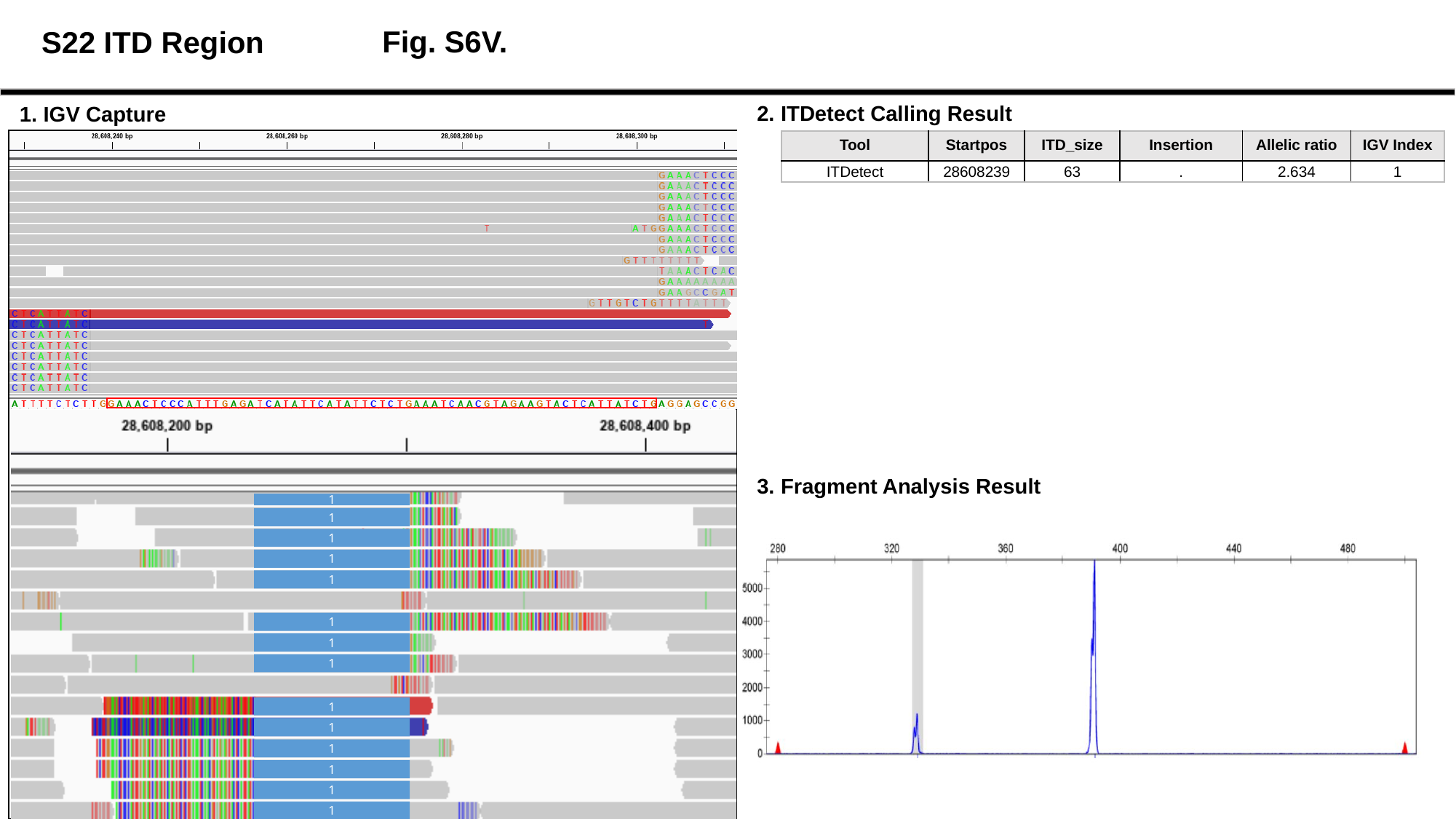

Fig. S6V.
S22 ITD Region
2. ITDetect Calling Result
1. IGV Capture
| Tool | Startpos | ITD\_size | Insertion | Allelic ratio | IGV Index |
| --- | --- | --- | --- | --- | --- |
| ITDetect | 28608239 | 63 | . | 2.634 | 1 |
3. Fragment Analysis Result
1
1
1
1
1
1
1
1
1
1
1
1
1
1

## Slide 38
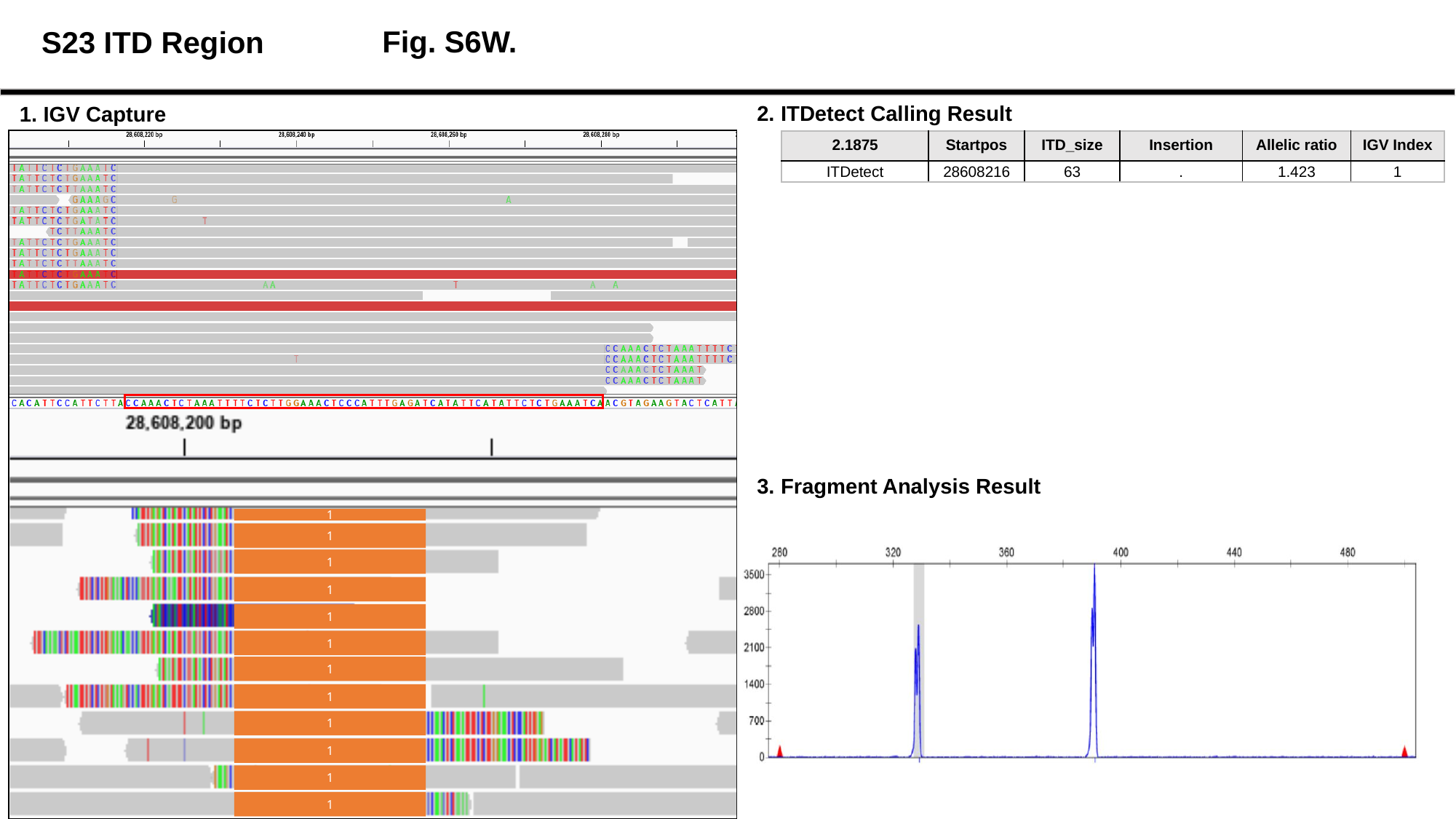

Fig. S6W.
S23 ITD Region
2. ITDetect Calling Result
1. IGV Capture
| 2.1875 | Startpos | ITD\_size | Insertion | Allelic ratio | IGV Index |
| --- | --- | --- | --- | --- | --- |
| ITDetect | 28608216 | 63 | . | 1.423 | 1 |
3. Fragment Analysis Result
1
1
1
1
1
1
1
1
1
1
1
1

## Slide 39
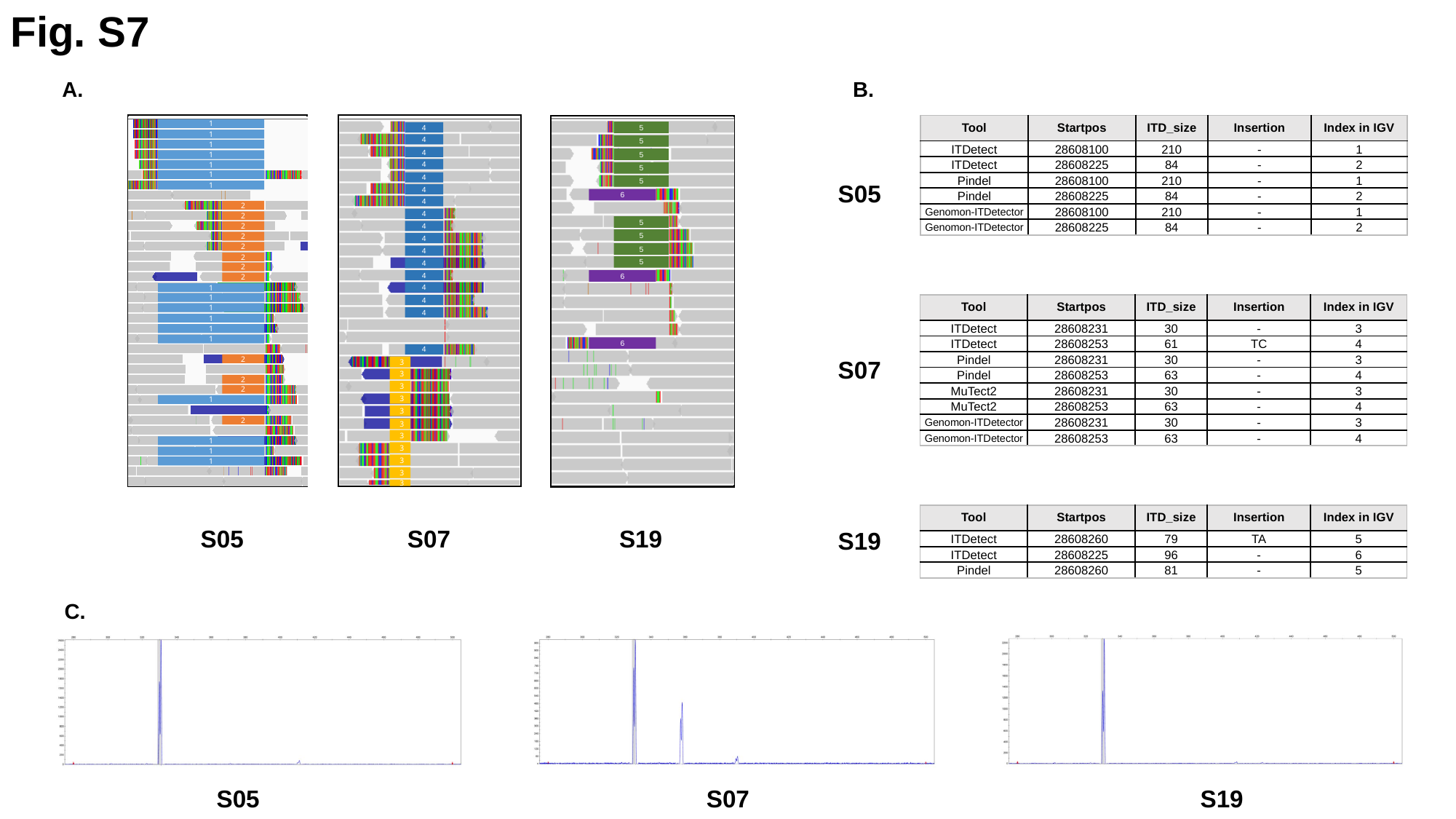

Fig. S7
A.
B.
1
5
4
1
4
5
1
4
5
1
4
1
5
1
4
5
1
4
6
4
2
4
2
5
4
2
5
2
4
2
5
4
2
5
4
2
6
4
2
4
1
1
4
1
4
1
1
1
6
4
2
3
3
2
3
2
3
1
3
2
3
3
1
3
1
3
1
3
3
| Tool | Startpos | ITD\_size | Insertion | Index in IGV |
| --- | --- | --- | --- | --- |
| ITDetect | 28608100 | 210 | - | 1 |
| ITDetect | 28608225 | 84 | - | 2 |
| Pindel | 28608100 | 210 | - | 1 |
| Pindel | 28608225 | 84 | - | 2 |
| Genomon-ITDetector | 28608100 | 210 | - | 1 |
| Genomon-ITDetector | 28608225 | 84 | - | 2 |
S05
| Tool | Startpos | ITD\_size | Insertion | Index in IGV |
| --- | --- | --- | --- | --- |
| ITDetect | 28608231 | 30 | - | 3 |
| ITDetect | 28608253 | 61 | TC | 4 |
| Pindel | 28608231 | 30 | - | 3 |
| Pindel | 28608253 | 63 | - | 4 |
| MuTect2 | 28608231 | 30 | - | 3 |
| MuTect2 | 28608253 | 63 | - | 4 |
| Genomon-ITDetector | 28608231 | 30 | - | 3 |
| Genomon-ITDetector | 28608253 | 63 | - | 4 |
S07
| Tool | Startpos | ITD\_size | Insertion | Index in IGV |
| --- | --- | --- | --- | --- |
| ITDetect | 28608260 | 79 | TA | 5 |
| ITDetect | 28608225 | 96 | - | 6 |
| Pindel | 28608260 | 81 | - | 5 |
S05
S07
S19
S19
C.
S05
S07
S19

## Slide 40
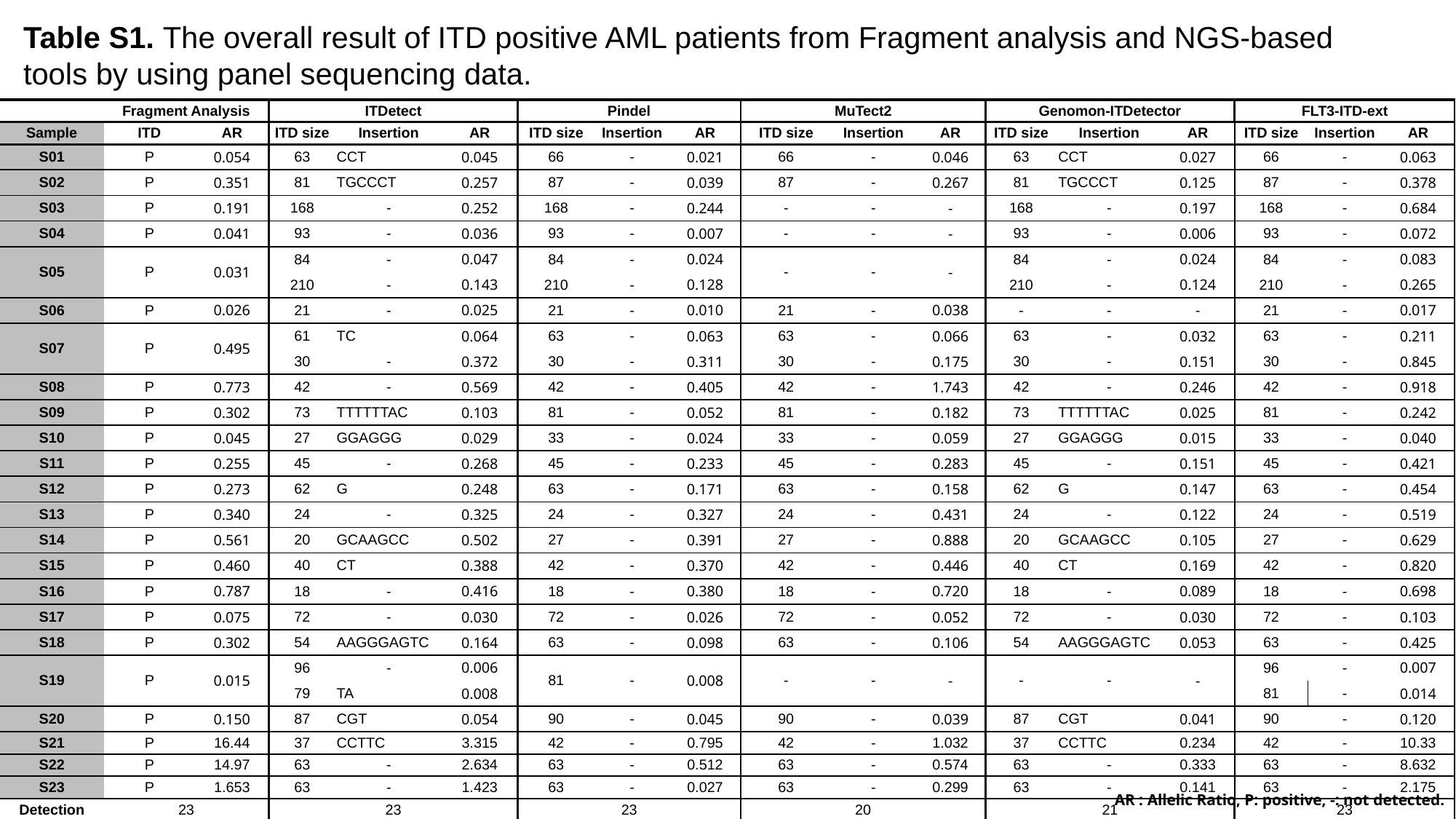

Table S1. The overall result of ITD positive AML patients from Fragment analysis and NGS-based tools by using panel sequencing data.
| | Fragment Analysis | | ITDetect | | | Pindel | | | MuTect2 | | | Genomon-ITDetector | | | FLT3-ITD-ext | | |
| --- | --- | --- | --- | --- | --- | --- | --- | --- | --- | --- | --- | --- | --- | --- | --- | --- | --- |
| Sample | ITD | AR | ITD size | Insertion | AR | ITD size | Insertion | AR | ITD size | Insertion | AR | ITD size | Insertion | AR | ITD size | Insertion | AR |
| S01 | P | 0.054 | 63 | CCT | 0.045 | 66 | - | 0.021 | 66 | - | 0.046 | 63 | CCT | 0.027 | 66 | - | 0.063 |
| S02 | P | 0.351 | 81 | TGCCCT | 0.257 | 87 | - | 0.039 | 87 | - | 0.267 | 81 | TGCCCT | 0.125 | 87 | - | 0.378 |
| S03 | P | 0.191 | 168 | - | 0.252 | 168 | - | 0.244 | - | - | - | 168 | - | 0.197 | 168 | - | 0.684 |
| S04 | P | 0.041 | 93 | - | 0.036 | 93 | - | 0.007 | - | - | - | 93 | - | 0.006 | 93 | - | 0.072 |
| S05 | P | 0.031 | 84 | - | 0.047 | 84 | - | 0.024 | - | - | - | 84 | - | 0.024 | 84 | - | 0.083 |
| | | | 210 | - | 0.143 | 210 | - | 0.128 | | | | 210 | - | 0.124 | 210 | - | 0.265 |
| S06 | P | 0.026 | 21 | - | 0.025 | 21 | - | 0.010 | 21 | - | 0.038 | - | - | - | 21 | - | 0.017 |
| S07 | P | 0.495 | 61 | TC | 0.064 | 63 | - | 0.063 | 63 | - | 0.066 | 63 | - | 0.032 | 63 | - | 0.211 |
| | | | 30 | - | 0.372 | 30 | - | 0.311 | 30 | - | 0.175 | 30 | - | 0.151 | 30 | - | 0.845 |
| S08 | P | 0.773 | 42 | - | 0.569 | 42 | - | 0.405 | 42 | - | 1.743 | 42 | - | 0.246 | 42 | - | 0.918 |
| S09 | P | 0.302 | 73 | TTTTTTAC | 0.103 | 81 | - | 0.052 | 81 | - | 0.182 | 73 | TTTTTTAC | 0.025 | 81 | - | 0.242 |
| S10 | P | 0.045 | 27 | GGAGGG | 0.029 | 33 | - | 0.024 | 33 | - | 0.059 | 27 | GGAGGG | 0.015 | 33 | - | 0.040 |
| S11 | P | 0.255 | 45 | - | 0.268 | 45 | - | 0.233 | 45 | - | 0.283 | 45 | - | 0.151 | 45 | - | 0.421 |
| S12 | P | 0.273 | 62 | G | 0.248 | 63 | - | 0.171 | 63 | - | 0.158 | 62 | G | 0.147 | 63 | - | 0.454 |
| S13 | P | 0.340 | 24 | - | 0.325 | 24 | - | 0.327 | 24 | - | 0.431 | 24 | - | 0.122 | 24 | - | 0.519 |
| S14 | P | 0.561 | 20 | GCAAGCC | 0.502 | 27 | - | 0.391 | 27 | - | 0.888 | 20 | GCAAGCC | 0.105 | 27 | - | 0.629 |
| S15 | P | 0.460 | 40 | CT | 0.388 | 42 | - | 0.370 | 42 | - | 0.446 | 40 | CT | 0.169 | 42 | - | 0.820 |
| S16 | P | 0.787 | 18 | - | 0.416 | 18 | - | 0.380 | 18 | - | 0.720 | 18 | - | 0.089 | 18 | - | 0.698 |
| S17 | P | 0.075 | 72 | - | 0.030 | 72 | - | 0.026 | 72 | - | 0.052 | 72 | - | 0.030 | 72 | - | 0.103 |
| S18 | P | 0.302 | 54 | AAGGGAGTC | 0.164 | 63 | - | 0.098 | 63 | - | 0.106 | 54 | AAGGGAGTC | 0.053 | 63 | - | 0.425 |
| S19 | P | 0.015 | 96 | - | 0.006 | 81 | - | 0.008 | - | - | - | - | - | - | 96 | - | 0.007 |
| | | | 79 | TA | 0.008 | | | | | | | | | | 81 | - | 0.014 |
| S20 | P | 0.150 | 87 | CGT | 0.054 | 90 | - | 0.045 | 90 | - | 0.039 | 87 | CGT | 0.041 | 90 | - | 0.120 |
| S21 | P | 16.44 | 37 | CCTTC | 3.315 | 42 | - | 0.795 | 42 | - | 1.032 | 37 | CCTTC | 0.234 | 42 | - | 10.33 |
| S22 | P | 14.97 | 63 | - | 2.634 | 63 | - | 0.512 | 63 | - | 0.574 | 63 | - | 0.333 | 63 | - | 8.632 |
| S23 | P | 1.653 | 63 | - | 1.423 | 63 | - | 0.027 | 63 | - | 0.299 | 63 | - | 0.141 | 63 | - | 2.175 |
| Detection | 23 | | 23 | | | 23 | | | 20 | | | 21 | | | 23 | | |
AR : Allelic Ratio, P: positive, -: not detected.

## Slide 41
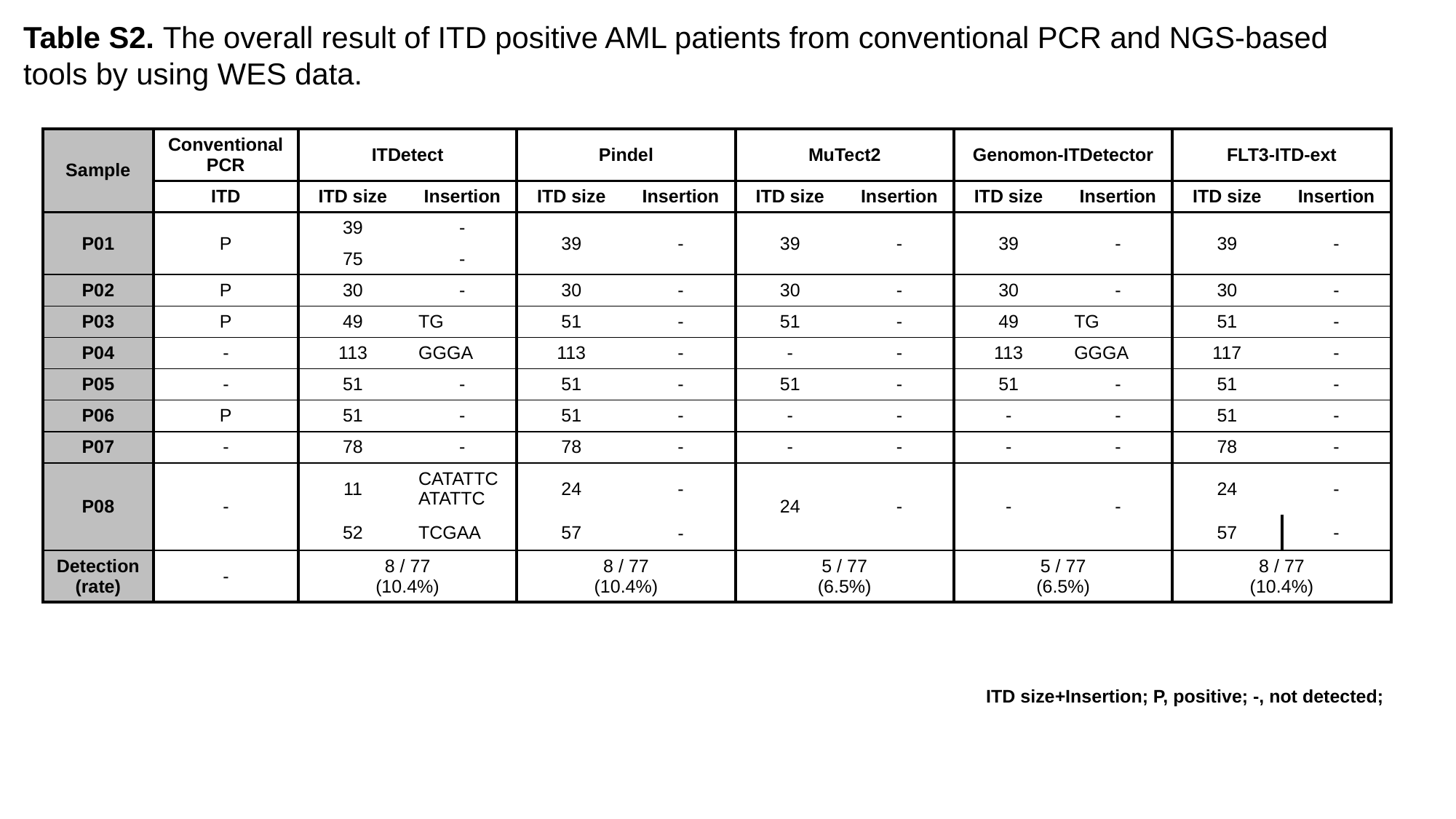

Table S2. The overall result of ITD positive AML patients from conventional PCR and NGS-based tools by using WES data.
| Sample | Conventional PCR | ITDetect | | Pindel | | MuTect2 | | Genomon-ITDetector | | FLT3-ITD-ext | |
| --- | --- | --- | --- | --- | --- | --- | --- | --- | --- | --- | --- |
| | ITD | ITD size | Insertion | ITD size | Insertion | ITD size | Insertion | ITD size | Insertion | ITD size | Insertion |
| P01 | P | 39 | - | 39 | - | 39 | - | 39 | - | 39 | - |
| | | 75 | - | | | | | | | | |
| P02 | P | 30 | - | 30 | - | 30 | - | 30 | - | 30 | - |
| P03 | P | 49 | TG | 51 | - | 51 | - | 49 | TG | 51 | - |
| P04 | - | 113 | GGGA | 113 | - | - | - | 113 | GGGA | 117 | - |
| P05 | - | 51 | - | 51 | - | 51 | - | 51 | - | 51 | - |
| P06 | P | 51 | - | 51 | - | - | - | - | - | 51 | - |
| P07 | - | 78 | - | 78 | - | - | - | - | - | 78 | - |
| P08 | - | 11 | CATATTCATATTC | 24 | - | 24 | - | - | - | 24 | - |
| | | 52 | TCGAA | 57 | - | | | | | 57 | - |
| Detection (rate) | - | 8 / 77 (10.4%) | | 8 / 77 (10.4%) | | 5 / 77 (6.5%) | | 5 / 77 (6.5%) | | 8 / 77 (10.4%) | |
ITD size+Insertion; P, positive; -, not detected;

## Slide 42
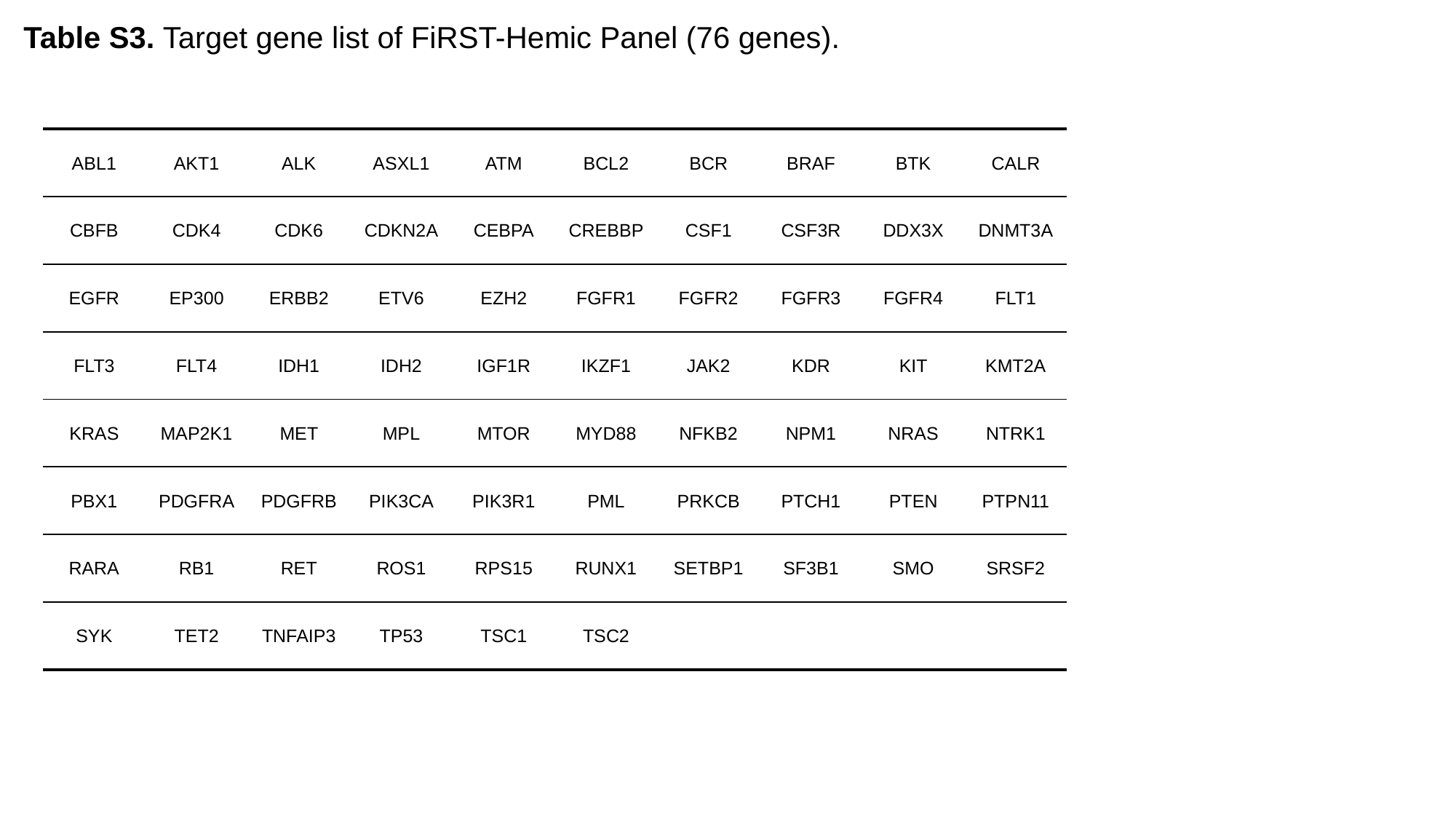

Table S3. Target gene list of FiRST-Hemic Panel (76 genes).
| ABL1 | AKT1 | ALK | ASXL1 | ATM | BCL2 | BCR | BRAF | BTK | CALR |
| --- | --- | --- | --- | --- | --- | --- | --- | --- | --- |
| CBFB | CDK4 | CDK6 | CDKN2A | CEBPA | CREBBP | CSF1 | CSF3R | DDX3X | DNMT3A |
| EGFR | EP300 | ERBB2 | ETV6 | EZH2 | FGFR1 | FGFR2 | FGFR3 | FGFR4 | FLT1 |
| FLT3 | FLT4 | IDH1 | IDH2 | IGF1R | IKZF1 | JAK2 | KDR | KIT | KMT2A |
| KRAS | MAP2K1 | MET | MPL | MTOR | MYD88 | NFKB2 | NPM1 | NRAS | NTRK1 |
| PBX1 | PDGFRA | PDGFRB | PIK3CA | PIK3R1 | PML | PRKCB | PTCH1 | PTEN | PTPN11 |
| RARA | RB1 | RET | ROS1 | RPS15 | RUNX1 | SETBP1 | SF3B1 | SMO | SRSF2 |
| SYK | TET2 | TNFAIP3 | TP53 | TSC1 | TSC2 | | | | |

## Slide 43
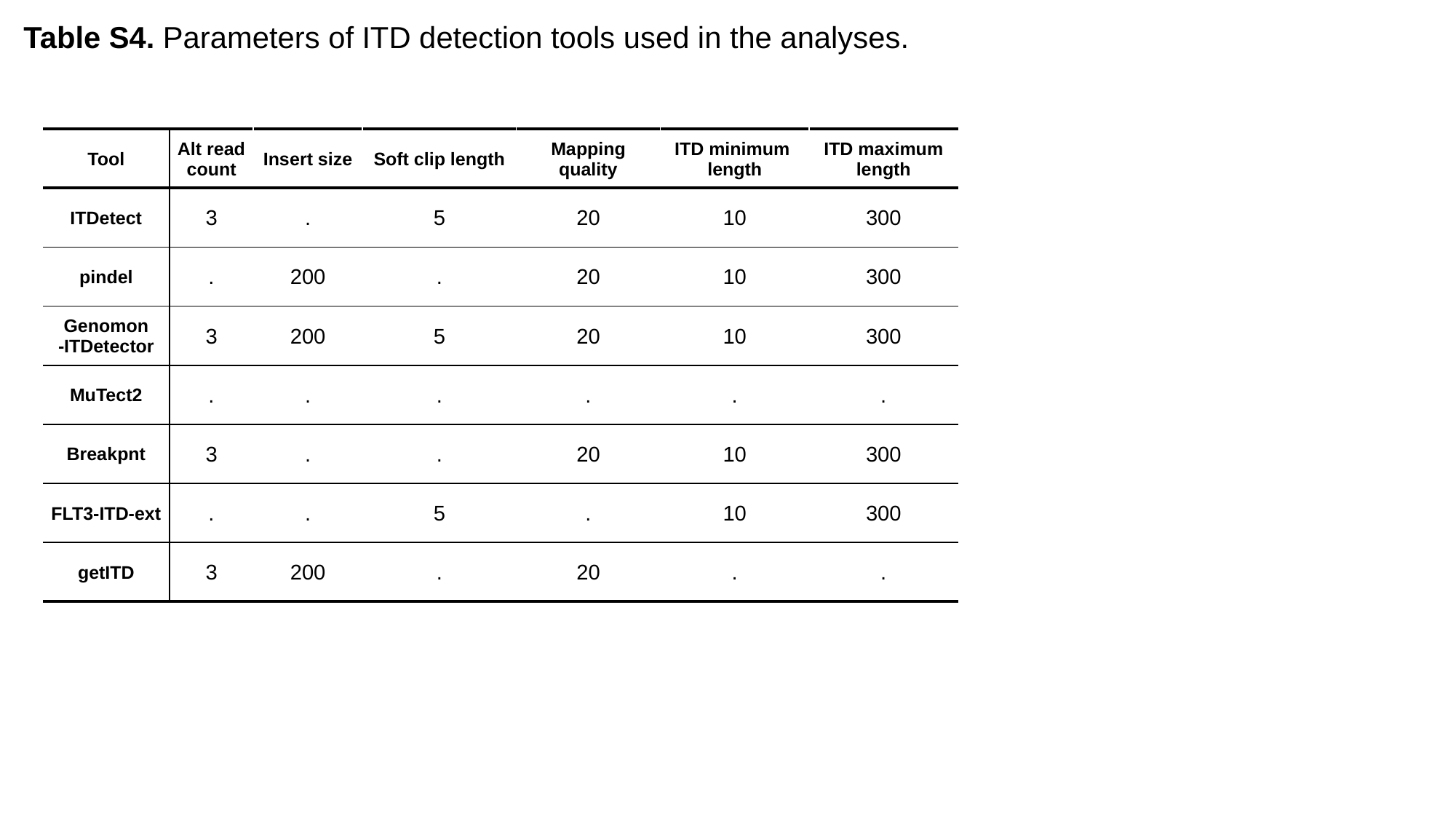

Table S4. Parameters of ITD detection tools used in the analyses.
| Tool | Alt read count | Insert size | Soft clip length | Mapping quality | ITD minimum length | ITD maximum length |
| --- | --- | --- | --- | --- | --- | --- |
| ITDetect | 3 | . | 5 | 20 | 10 | 300 |
| pindel | . | 200 | . | 20 | 10 | 300 |
| Genomon -ITDetector | 3 | 200 | 5 | 20 | 10 | 300 |
| MuTect2 | . | . | . | . | . | . |
| Breakpnt | 3 | . | . | 20 | 10 | 300 |
| FLT3-ITD-ext | . | . | 5 | . | 10 | 300 |
| getITD | 3 | 200 | . | 20 | . | . |
